# *In vitro* expression and analysis of the 826 human G protein-coupled receptors

**DOI:** 10.1007/s13238-016-0263-8

**Published:** 2016-04-16

**Authors:** Xuechen Lv, Junlin Liu, Qiaoyun Shi, Qiwen Tan, Dong Wu, John J. Skinner, Angela L. Walker, Lixia Zhao, Xiangxiang Gu, Na Chen, Lu Xue, Pei Si, Lu Zhang, Zeshi Wang, Vsevolod Katritch, Zhi-jie Liu, Raymond C. Stevens

**Affiliations:** iHuman Institute, ShanghaiTech University, Shanghai, 201210 China; Department of Biological Sciences, Bridge Institute, University of Southern California, Los Angeles, CA 90089 USA

**Keywords:** G protein-coupled receptors, insect, protein expression, surface expression analysis, fusion construct

## Abstract

G protein-coupled receptors (GPCRs) are involved in all human physiological systems where they are responsible for transducing extracellular signals into cells. GPCRs signal in response to a diverse array of stimuli including light, hormones, and lipids, where these signals affect downstream cascades to impact both health and disease states. Yet, despite their importance as therapeutic targets, detailed molecular structures of only 30 GPCRs have been determined to date. A key challenge to their structure determination is adequate protein expression. Here we report the quantification of protein expression in an insect cell expression system for all 826 human GPCRs using two different fusion constructs. Expression characteristics are analyzed in aggregate and among each of the five distinct subfamilies. These data can be used to identify trends related to GPCR expression between different fusion constructs and between different GPCR families, and to prioritize lead candidates for future structure determination feasibility.

**Electronic supplementary material:**

The online version of this article (doi:10.1007/s13238-016-0263-8) contains supplementary material, which is available to authorized users.

## INTRODUCTION

**Figure 1:**
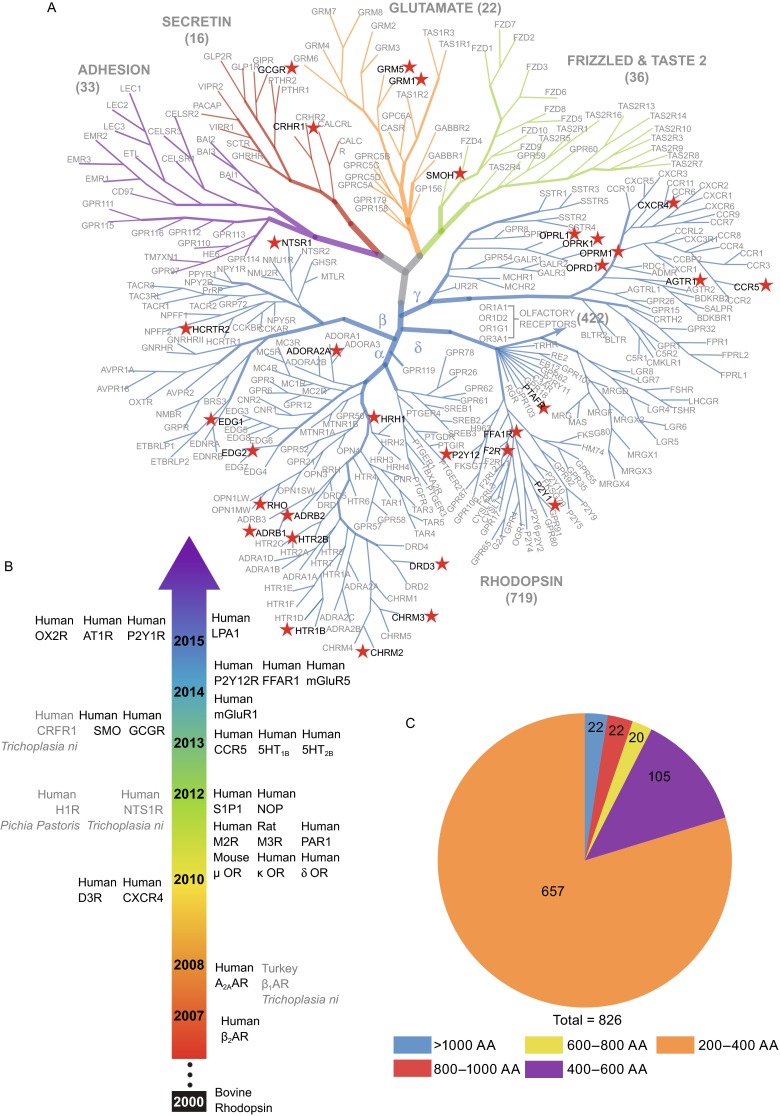
Overview of human GPCRs. **(**A) A Family tree of the 826 human GPCRs. (B) Including Rhodopsin in 2000, structures have been determined for 30 distinct GPCRs to date. Of these, the vast majority were expressed in *Sf9* cells and GPCRs expressed in other kinds of cell lines are marked in *gray*. (C) The majority of GPCRs consist of 200–400 amino acids, with GPR98 from the Adhesion family the single largest at 6,306 amino acids

G protein-coupled receptors (GPCRs) are of great importance for physiological function and constitute the largest family of human membrane proteins, with 826 members (Fig. 1A and Table 1). Also termed seven transmembrane (7TM) receptors because of their conserved core architecture of seven transmembrane alpha-helices, GPCRs can recognize and bind many diverse signaling molecules including odorants, neurotransmitters and hormones (Stevens et al., 2013). Drugs targeting GPCRs comprise as much as 40% of all marketed drugs, and the receptors are implicated in many medical conditions such as heart disease, neurological disorders, cancer and obesity (Rask-Andersen et al., 2014).

**Table 1:** The families of 826 GPCRs and their structures

Family	# Of receptors	# Of structures currently available
Rhodopsin	719	25
Secretin	16	2
Glutamate	22	2
Frizzled/Taste 2	36	1
Adhesion	33	0
Total	826	30

 Detailed three-dimensional structural information is of great importance for understanding the physiological functions of GPCRs and for designing new drugs to target them. In recent years, persistent efforts of researchers and implementation of new technologies have contributed to the accelerated development of GPCR structural studies. In 2000, the first mammalian GPCR structure was elucidated (Palczewski et al., 2000). Since then, the structures of 30 different GPCRs (Fig. 1A, 1B and Table 1) have been reported. While this represents real progress, it comprises only a fraction of almost 300 GPCRs that are known to be involved in psychiatric diseases, cancer, and other maladies, and an even smaller fraction of the 826 GPCRs found in humans (Katritch et al., 2013).

Given the challenges in structurally determining GPCRs and the large number of structures that remain to be solved, one approach to maintain the recently developed momentum is to prioritize those GPCRs with the highest likelihood of success. As protein expression is the critical first step in the structure determination process, it makes sense to pursue the receptors with high expression levels first as these are most likely to provide the highest yield after purification. In this study, we applied a comprehensive family-wide approach to express all 826 human GPCRs using two different construct designs. The comprehensive results (Table S1) are provided to facilitate future biochemical, pharmacological, and structural studies.

## APPROACH

**Figure 2:**
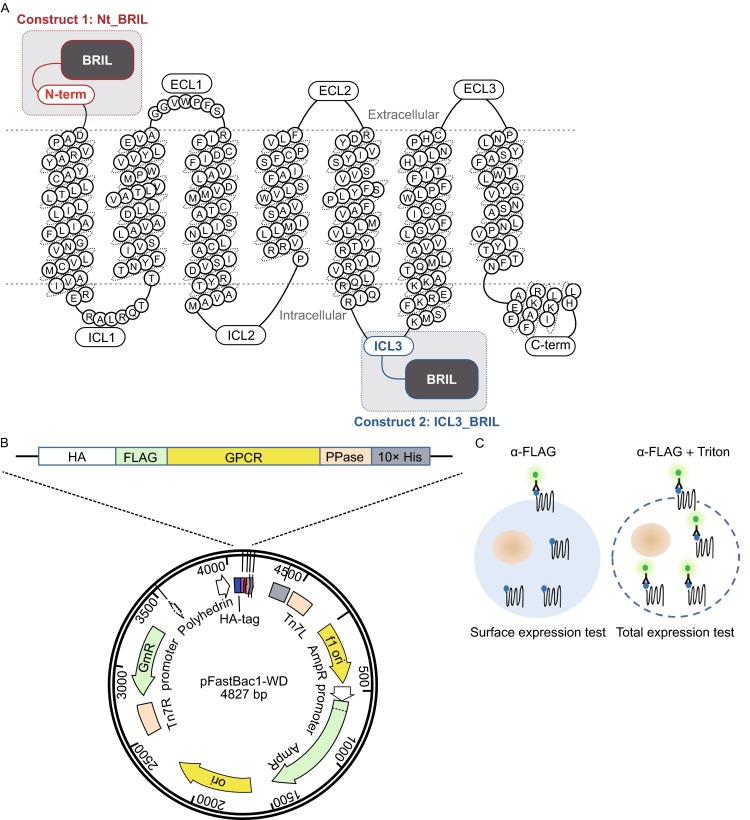
Experimental design. (A) Constructs were designed by truncating the flexible N- and C-termini and inserting BRIL at either the N-terminus (Nt_BRIL) or intracellular loop 3 (ICL3_BRIL). (B) Constructs were then inserted into the pFastBac 1 vector for *Sf9* expression. (C) FITC fluorescence was used to measure the percentage of cells expressing the GPCR, as well as density of GPCRs for those cells. Surface and total expression were measured by adding α-flag FITC with and without Triton, respectively

In order to evaluate the relative expression levels of all 826 human GPCRs, we developed a simple strategy that could be applied uniformly to each receptor involving construct design, expression, and quantification. GPCRs can vary greatly in length, some having more than 1000 residues, but most consist of 200–400 residues (Fig. 1C), primarily constituting the 7TM helices. While the full length protein is undoubtedly important for native *in vivo* function, in these studies we have focused on the receptors’ 7TM domain. Thus, the first step in construct design was to truncate the flexible N- and C-termini based on the computationally predicted 7TM regions (See “MATERIALS AND METHODS”). The second step in construct design was to add a fusion partner. Fusion partners have often been useful for increasing expression and stabilizing membrane proteins (Chun et al., 2012). Here we used Cytochrome b_562_ RIL (BRIL), a soluble alpha-helical protein that has been crystallized and structurally characterized by itself to a resolution of 1.8 Å (PDB ID 1M6T, MW 11.9 kDa) and with the A_2A_ adenosine receptor to a resolution of 1.8 Å (PDB ID 4EIY). Two constructs were designed and generated for each GPCR, one with BRIL attached at the N-terminus truncation site (Nt_BRIL) and one with BRIL inserted in intracellular loop 3 (ICL3_BRIL; Fig. 2A) as described in MATERIALS AND METHODS. Both design approaches have led to crystallographic characterization of several GPCRs, including 1.8 Å resolution structures of the A_2A_ adenosine and delta-opioid receptors (Liu et al., 2012; Fenalti et al., 2014).

Constructs were then cloned into a modified pFastBac1 vector for expression in *Spodoptera frugiperda* (*Sf9*) cells (See “MATERIALS AND METHODS”). *Sf9* cells were selected based on their demonstrated success in other GPCR structural studies. Four types of expression systems have been employed in protein production for structural studies of GPCRs to date: *E. coli*, yeast, mammalian cells and insect cells (Zhao and Wu, 2012). We chose the *Spodoptera frugiperda* (*Sf9*) expression system as it presently has the most established track record, given that 25 of the 30 structurally determined GPCRs were expressed in this system (Fig. 1B).

In this study, expression levels were detected using a fluorescent probe that consists of an α-flag FITC-coupled antibody that specifically recognizes a FLAG sequence inserted at the N-terminus of each construct (Fig. 2). Receptor cell surface expression and total receptor expression was determined by flow cytometry using a fluorescence signal detected from cells pre-incubated with the fluorescent probe in the absence (For surface expression % and surface density values) or presence of a mild detergent (For total expression % and total density values), respectively. This approach allowed us to quantify the percentage of cells expressing GPCRs, as well as the relative receptor expression, at the surface or overall (total).

## RESULTS

### General GPCR expression levels

In this project a total of 1652 constructs, 826 Nt_BRIL constructs, and 826 ICL3_BRIL constructs, were cloned and expressed as summarized individually in Table S1 and collectively in Table 2. Of all these, about 7% (119 of 1,652) show a high level of expression (surface expression >80%; Table 3).

**Table 2:** Statistics of expression levels among the 1,652 GPCR constructs^1^

Construct(s)	% Surface expression^2^	Surface density^3^	% Total expression^4^	Total density^5^
Range	1.85–97.00	11.77–865.22	0.50–98.75	13.80– 812.69
Mean	44.71	74.14	75.84	258.09
Median	42.98	57.04	85.18	145.50
25th percentile	26.74	32.80	61.14	60.50
75th percentile	60.56	95.45	93.60	403.52

^1^Most of indexes obey a skewed distribution except for surface percentage. The median and the quartiles of 1,652 constructs were obtained by the SPSS

^2^Surface expression: The ratio of all the cells expressing the target receptors on the membrane to total cells

^3^Surface density: The ratio of all the fluorescence of membrane proteins to total cells

^4^Total expression: The ratio of cells expressing the target receptors to total cells

^5^Total density: The ratio of all the fluorescence to total cells

**Table 3:** High expressing GPCR constructs by family^1^

Family	Nt_BRIL	ICL3_BRIL	Total^2^	Non-duplicates^3^	No current structure^4^
Rhodopsin	26	45	71	61	53
Secretin	2	5	7	6	6
Glutamate	9	3	12	9	9
Frizzled/Taste 2	7	3	10	8	7
Adhesion	10	8	18	12	12
Total	54	64	118	96	87

^1^High expression defined as >80% surface expression, which is the ratio of all the cells expressing the target receptors on the membrane to total cells

^2^Sum of high expressing Nt_BRIL and ICL3_BRIL constructs

^3^Total of unique GPCRs with high expression (counting the GPCRs that showed high surface expression in both Nt_BRIL and ICL3_BRIL constructs only once)

^4^The number of unique GPCRs with high expression for which no three-dimensional structure is currently available

### Comparison of expression between Nt_BRIL and ICL3_BRIL constructs

**Figure 3:**
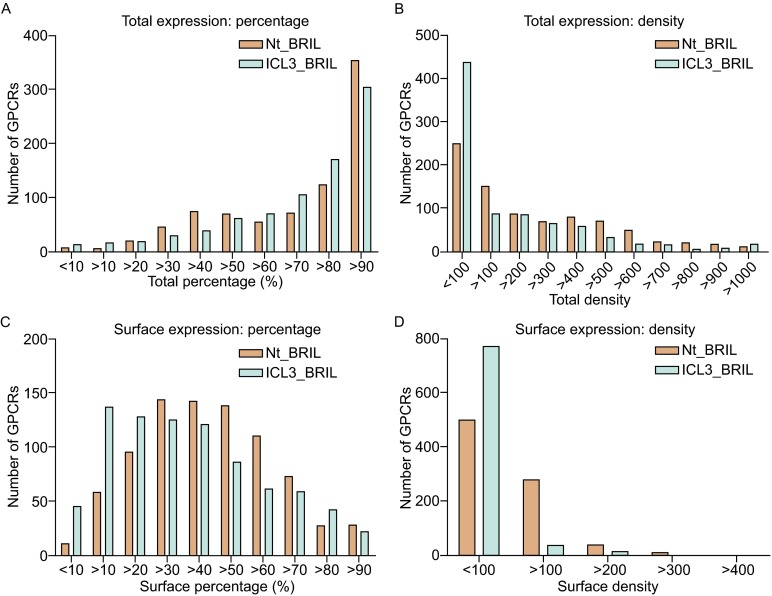
Distribution of total and surface expression among constructs. Comparison of total expression percentage (A), total expression density (B), surface expression percentage (C), and surface expression density (D) between Nt_BRIL and ICL3_BRIL constructs collectively

The BRIL soluble domain was inserted into the GPCR to promote expression by stabilizing the receptor and increasing solubility (Fig. 2A). As expected, expression levels varied between the two different constructs of each receptor. In this study, the Nt_BRIL construct was generally more effective than ICL3_BRIL construct at promoting both total expression and surface expression (Fig. 3 and Table 4). Twenty-eight of the Nt_BRIL constructs displayed surface expression >90% versus 22 of the ICL3_BRIL constructs; 325 of the Nt_BRIL constructs had a surface density above 100 MFU (mean fluorescence units) versus 53 of the ICL3_BRIL constructs. The majority of Nt_BRIL constructs (424) had surface expression levels between 30%–60%, while most of ICL3_BRIL constructs (390) had surface expression levels between 10%–40%. For the Nt_BRIL constructs, the number of GPCRs with total percent expression >90% is larger (358) than for ICL3_BRIL constructs (306).

**Table 4:** Statistics of GPCR expression levels by construct

Construct(s)	% Surface expression^1^	Surface density^2^	% Total expression^3^	Total density^4^
Nt_BRIL construct				
Range	1.85–97.00	18.51–481.97	1.25–98.75	18.82–1812.69
Mean	48.12	100.83	75.94	306.91
Median	46.63	85.64	86.95	210.55
25th percentile	32.95	62.73	58.26	86.12
75th percentile	62.85	124.80	94.45	480.28
ICL3_BRIL construct				
Range	3.45–96.65	11.77–865.22	0.50–98.50	13.80–1478.02
Mean	41.29	47.44	75.74	209.27
Median	38.35	33.91	84.05	88.51
25th percentile	21.59	24.13	63.51	37.85
75th percentile	56.58	50.45	92.74	306.69

^1^Surface expression: The ratio of all the cells expressing the target receptors on the membrane to total cells

^2^Surface density: The ratio of all the fluorescence of membrane proteins to total cells

^3^Total expression: The ratio of cells expressing the target receptors to total cells

^4^Total density: The ratio of all the fluorescence to total cells

High expression for the Nt_BRIL construct of a receptor did not always correspond to high expression for the ICL3_BRIL construct. For example, 54 Nt_BRIL and 65 ICL3_BRIL constructs had surface expression levels >80%, yet only 22 receptors expressed at this level for both constructs (Tables S2–S4). Similarly, 164 Nt_BRIL and 309 ICL3_BRIL constructs had surface expression <30%, compared to 94 receptors with low expression for both constructs.

**Figure 4:**
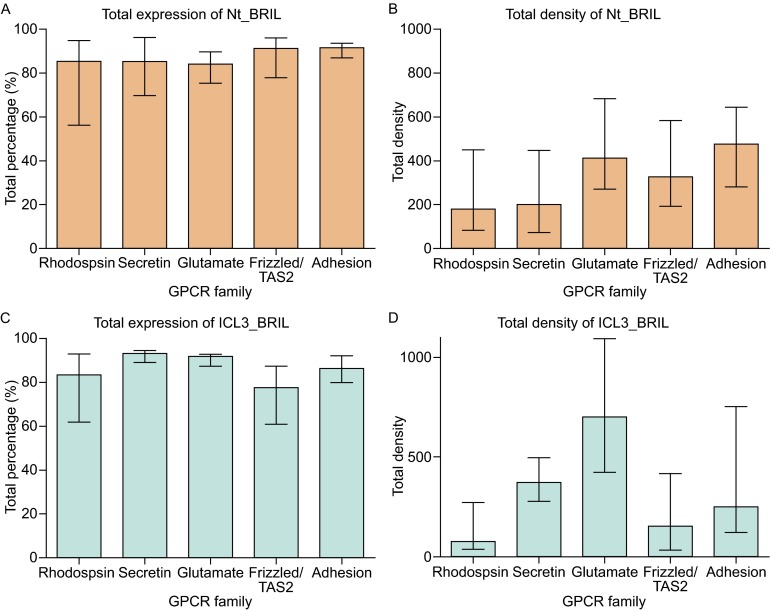
Total expression characteristics by GPCR family and construct. Distributions among GPCR families are shown for total percentage of cells expressing GPCRs (A and C) and total density of GPCRs (B and D) for Nt_BRIL (A and B) and ICL3_BRIL (C and D) constructs. Graphs are plotted as median values for the Rhodopsin, Secretin, Glutamate, Frizzled/Taste2, and Adhesion families with 719, 16, 22, 36, and 33 members, respectively. Error bars indicate first quartile from the median

In an attempt to determine a pattern in receptor preference for Nt_BRIL versus ICL3_BRIL, we grouped and analyzed expression data according to receptor family (Fig. 4). Few differences were found between GPCR families in terms of the percentage of cells that expressed either construct (Fig. 4A and 4C). Total receptor expression density varied more from family to family, with Glutamate and Adhesion family receptors exhibiting the highest expression density for Nt_BRIL constructs (Fig. 4B). In general, Nt_BRIL constructs had higher expression density than ICL3_BRIL constructs for Rhodopsin, Frizzled/Taste2, and Adhesion family receptors, while ICL3_BRIL constructs performed better for Glutamate family receptors.

**Figure 5:**
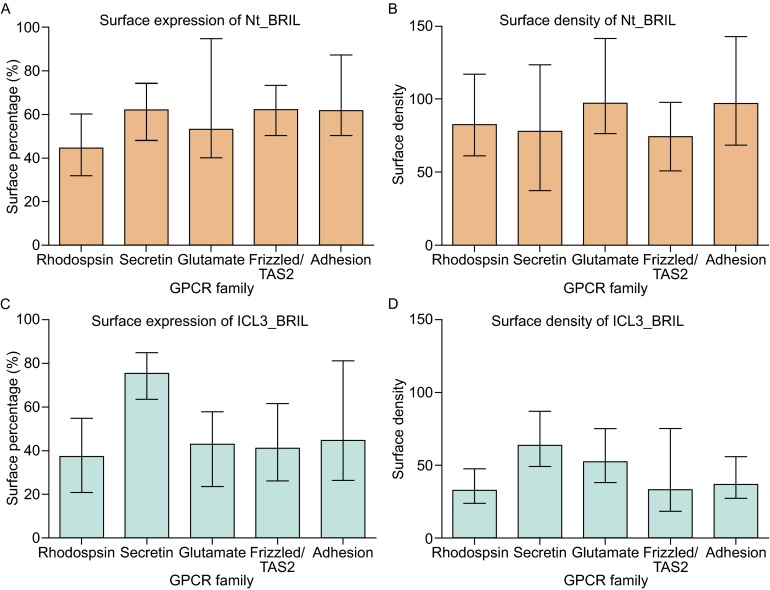
Surface expression by GPCR family. Distributions among GPCR families are shown for percentage of cells expressing GPCRs on their surface (A and C) and density of GPCRs on the surface (B and D) for Nt_BRIL (A and B) and ICL3_BRIL (C and D) constructs. Error bars indicate first quartile from the median. Graphs are plotted as median values for the Rhodopsin, Secretin, Glutamate, Frizzled/Taste2, and Adhesion families with 719, 16, 22, 36, and 33 members, respectively. Error bars indicate first quartile from the median

The average percentage of cells expressing receptors on their surface was fairly constant across the families for Nt_BRIL constructs (Fig. 5A). ICL3_BRIL constructs, on the other hand, exhibited much higher surface expression percentages for Secretin family receptors than for other families (Fig. 5C). Similarly, Secretin family receptors exhibited the highest surface density for ICL3_BRIL constructs (Fig. 5D).

### Expression between the GPCR families

The expression levels are summarized in Table 5 from the data analysis of the different GPCR families. The surface density represents the mean fluorescence intensity of all cells expressing the target receptors, and the surface percentage represents the ratio of cells expressing the target receptor from the total number of cells. Here we compared the surface expression percentage, surface density, total expression percentage and total density of different families. For the Nt_BRIL constructs, the surface expression level order between families is: Frizzled/Taste2 = Secretin = Adhesion > Glutamate > Rhodopsin. With the BRIL fusion at the N-termini, the Secretin family showed the best expression levels in these *Sf9* cultures. For the ICL3_BRIL constructs, the Secretin family constructs had the highest surface percentage and surface density. On the contrary, the ICL3_BRIL constructs of the Rhodopsin family showed the lowest surface percentage and lowest expression level.

**Table 5:** Median expression levels by family and construct^1^

Construct(s)	% Surface expression^2^	Surface density^3^	% Total expression^4^	Total density^5^
Rhodopsin				
Nt_BRIL	44.35	84.62	85.45	179.45
ICL3_BRIL	37.15	33.01	83.10	73.38
Secretin				
Nt_BRIL	62.28	86.23	85.30	200.18
ICL3_BRIL	75.43	63.79	93.13	371.82
Glutamate				
Nt_BRIL	53.03	124.81	84.00	413.80
ICL3_BRIL	42.83	53.27	91.70	700.13
Frizzled/Taste 2				
Nt_BRIL	62.18	76.18	91.28	327.45
ICL3_BRIL	41.13	35.99	77.63	151.70
Adhesion				
Nt_BRIL	62.15	99.97	91.90	476.01
ICL3_BRIL	44.65	42.38	86.30	250.56

^1^Values are given as medians from the Rhodopsin, Secretin, Glutamate, Frizzled/Taste2, and Adhesion families with 719, 16, 22, 36, and 33 members, respectively

^2^Surface expression: The ratio of all the cells expressing the target receptors on the membrane to total cells

^3^Surface density: The ratio of all the fluorescence of membrane proteins to total cells

^4^Total expression: The ratio of cells expressing the target receptors to total cells

^5^Total density: The ratio of all the fluorescence to total cells

In Nt_BRIL constructs, Glutamate and Adhesion families showed the highest surface density. For the total density of ICL3_BRIL constructs, there are notable differences between the different families with expression ranking as: Glutamate > Secretin > Adhesion > Frizzled/Taste2 > Rhodopsin family (Fig. 4). When the fusion partner BRIL is inserted at ICL3, Glutamate family receptors collectively produced the best expression levels, although Rhodopsin family receptors constitute the majority of the receptors whose surface expression levels exceed 80% (Fig. 4).

For some receptors, the Nt_BRIL construct showed a high expression level, while the ICL3_BRIL construct showed low expression (Table 6). This pattern is observed in Rhodopsin, Secretin, Glutamate, and Frizzled/Taste2 receptor families, but potentially for different reasons (discussed below). For example, for the receptors CML1, CELR3, and FZD9—Frizzled/Taste2 family receptors—both the total density and surface density values of the Nt_BRIL constructs are higher than the ICL3_BRIL constructs, indicating the Nt_BRIL construct is not only expressed, but also secreted better than the ICL3_BRIL construct. For the GRM2 receptor—a Glutamate family member—although the surface density of its Nt_BRIL construct is higher than its ICL3_BRIL construct, for the total density, the result is just the opposite. This means that for the GRM2 receptor, the ICL3_BRIL construct was expressed much better than the Nt_BRIL construct inside the cell, so the receptor had some issue in terms of trafficking to the cell membrane in the ICL3_BRIL construct (Table 6).

**Table 6:** Representative receptors within GPCR families with high expressing Nt_BRIL constructs and low expressing ICL3_BRIL constructs

Construct(s)	% Surface expression^1^	Surface density^2^	% Total expression^3^	Total density^4^
CML1 (Rhodopsin)				
Nt_BRIL	82.80	50.80	93.25	88.44
ICL3_BRIL	37.35	32.94	45.35	35.94
CELR3 (Secretin)				
Nt_BRIL	94.40	163.13	92.55	812.34
ICL3_BRIL	3.50	137.70	0.50	108.65
GRM2 (Glutamate)				
Nt_BRIL	92.30	100.26	85.40	313.66
ICL3_BRIL	43.70	38.69	91.60	1404.69
FZD9 (Frizzled/Taste2)				
Nt_BRIL	94.15	197.92	91.20	592.72
ICL3_BRIL	31.35	99.11	71.50	437.76

^1^Surface expression: The ratio of all the cells expressing the target receptors on the membrane to total cells

^2^Surface density: The ratio of all the fluorescence of membrane proteins to total cells

^3^Total expression: The ratio of cells expressing the target receptors to total cells

^4^Total density: The ratio of all the fluorescence to total cells

Another interesting finding is that individual receptors in some subfamilies with high sequence conservation displayed expression levels with high variability between subfamily members and constructs. For example, the β-adrenergic receptors subfamily within the rhodopsin family is composed of three members. The sequence identity of the 7TM helices and loop regions between the β2- and β3-adrenergic receptors is very high (65%), but the receptors displayed different expression levels with Nt_BRIL and ICL3_BRIL constructs (Table 7 and Fig. S1). The same is true for adenosine subfamily receptors AA2AR and AA2BR, the sequence identity between these members is 61%, but the expression level of AA2BR is much lower than the AA2AR, regardless of construct in this study (Table 7). In the metabotropic glutamate and lysophosphatidic acid receptor subfamilies, mGluR7 and LPAR1 also show very low expression levels.

**Table 7:** Example of variance in expression despite high sequence similarity in the adrenergic and adensosine receptors within the Rhodopsin family

Receptors	Surface expression (%)		Surface density		Total expression (%)		Total density		Sequence identity	
Adrenergic	NB^1^	ICB^2^	NB	ICB	NB	ICB	NB	ICB	ADRB2 (%)	ADRB3 (%)
ADRB1	79.35	90.30	55.61	70.93	97.95	94.90	456.69	299.99	54	56
ADRB2	42.50	71.10	199.68	47.83	97.45	95.35	480.55	380.84	–	65
ADRB3	40.02	80.60	54.57	50.44	56.60	94.50	58.21	515.67	65	–

Receptors	Surface expression (%)		Surface density		Total expression (%)		Total density		Sequence identity
Adenosine	NB^1^	ICB^2^	NB	ICB	NB	ICB	NB	ICB	AA2BR (%)
AA2AR	96.2	91.45	184.82	142.46	81.2	77.9	211.41	286.44	61
AA2BR	31.7	23.80	115.3	30.81	41.15	79.15	483.4	110.84	–

^1^Nt_BRIL

^2^ICL3_BRIL

Finally, the Rhodopsin family can be further subdivided into olfactory and non-olfactory receptors with 422 and 297 members, respectively. The olfactory receptors did not express well with either fusion protein (Table 8), but generally did better with Nt_BRIL constructs (median surface percent = 38.6%) than with ICL3_BRIL constructs (median surface percent = 23.6%). Only 2 Nt_BRIL olfactory constructs had a surface percent above 80%, with 72 Nt_BRIL olfactory constructs above 60%; none of the ICL3_BRIL constructs had a surface percent above 80% and only 2 had a surface percent above 60%.

**Table 8:** The expression level of N_BRIL and ICL3-BRIL constructs of olfactory receptors and non-olfactory receptors from the Rhodopsin family

Construct(s)	% Surface expression^1^	Surface density^2^	% Total expression^3^	Total density^4^
Olfactory Rhodopsin (*n* = 422)				
Nt_BRIL	38.60	100.61	85.45	197.77
25th percentile	26.73	71.84	50.08	86.09
75th percentile	54.95	140.72	94.43	518.18
ICL3_BRIL	23.60	25.93	73.95	41.31
25th percentile	15.95	20.21	54.32	27.71
75th percentile	38.00	34.11	88.33	72.02
Non-Olfactory Rhodopsin (*n* = 297)				
Nt_BRIL	52.10	70.07	85.55	164.01
25th percentile	40.65	49.48	67.50	78.03
75th percentile	66.95	95.46	95.30	400.54
ICL3_BRIL	58.15	47.53	91.50	303.00
25th percentile	44.25	36.32	80.10	176.40
75th percentile	74.95	66.41	94.80	458.35

^1^Surface expression: The ratio of all the cells expressing the target receptors on the membrane to total cells

^2^Surface density: The ratio of all the fluorescence of membrane proteins to total cells

^3^Total expression: The ratio of cells expressing the target receptors to total cells

^4^Total density: The ratio of all the fluorescence to total cells

## DISCUSSION

### Identifying trends in the results

The GPCR structures that have been solved with fusion partners did not share the same precise placement location for their fusion partner, as in they did in this study, therefore, a lack of positional optimization can be expected when reviewing these results. Yet, we can define some general trends from the large amount of data collected in this study. Overall, the expression levels of the 826 Nt_BRIL GPCR constructs was higher than at the ICL3_BRIL constructs, it can be concluded that a well-organized N-terminus is helpful for effective trafficking of the post-translational receptor to cell membrane. Another possible conclusion is that the N-terminal fusion partner may make the tertiary structure more stable and less toxic to the cell as a result.

For the adrenergic receptors in the Rhodopsin family, β1 and β2 adrenergic receptors have high sequence identity. However, they displayed very different expression levels in this *Sf9* expression system. This is evident that the expression level or the property of receptors can be affected by very few residues. Just as in the construct optimization process, point mutation screening could identify a more stabilizing version of the protein (Zhang et al., 2014). From the expression data of the Frizzled/Taste2 family, it can be concluded that the expression level is closely related to the protein’s properties. In other words, a good expression level is one of the characteristics of a stable receptor.

The differences between non-olfactory and olfactory receptors within the Rhodopsin family are mainly reflected in longer extracellular loops and the conserved properties of the 7TM domain. After analysis of the receptor’s sequence data from Uniprot, generally, the length of extracellular loop 2 (ECL2) and ECL3 in most olfactory receptors was found to be more than 20 and 35 amino acids, respectively. However, for the non-olfactory receptors, either ECL1 or ECL2 is longer than 20 amino acids, or both loops are shorter than 20 amino acids. This observation is distinct from the trend of GPCRs in general, of which the 7TM helical bundle has been the most conserved component (Katritch et al., 2012), across the over 400 various odorant receptors (Jiang and Matsunami, 2015), the most conserved domains are the intracellular loops and the seventh transmembrane helix (helix VII), while the sequence diversity of helices III, IV, and V to which the odorant molecules bind is very high (Gao et al., 2010; de March et al., 2015). These two characteristics may contribute to the low expression level and instability of the olfactory receptors. From the perspective of function, one odorant can stimulate several kinds of odorant receptors, meanwhile one single odorant receptors can be activated by numerous different odorants (Sanz et al., 2014). Therefore the functional peculiarity of olfactory receptors may reflect their particularity in structure.

Glycosylation is also known to affect the ability of the receptor to reach the cell surface. This fact is especially relevant to some of the Glutamate family receptors, like GABA_B1_ and GPRC6. GABA_B1_ contains five N-glycosylation sites in the extracellular domain; when mutating all five sites, low surface expression was seen 24 h post-transfection (Deriu, 2005; Norskov-Lauritsen and Brauner-Osborne, 2015). GPRC6 was shown to be N-glycosylated at seven different sites *in vitro* in the extracellular domain. Mutation of any two sites was shown to affect the receptor’s surface expression (Norskov-Lauritsen and Brauner-Osborne, 2015; Norskov-Lauritsen et al., 2015). However, not all the Glutamate family receptors require glycosylation to maintain surface expression. For example, the inhibition N-glycosylation of mGlu1R did not change its surface expression level (Mody, 1999; Norskov-Lauritsen and Brauner-Osborne, 2015). In this study, truncation of the extracellular domain which contains most of the glycosylation sites contributed to the low expression levels of both GABA_B1_ and GPRC6A receptors.

Finally, the expression level on the membrane maybe also affected by the exogenous environment. If one receptor is co-expressed or interacts with another receptor in its native physiological environment, the receptor maybe unstable and expressed poorly in the heterologous experimental system.

The expression study of these 1,652 GPCR constructs identified some familial trends, and importantly, identified several high expressing GPCRs for which no structural data currently exists. Based on these findings, future studies can prioritize work on these high expressing receptors and work to further optimize the construct and identify stabilizing ligands to assist with elucidation of the protein’s three dimensional structure.

## MATERIALS AND METHODS

### Construct design

Design of truncations and BRIL fusion sites was based on similarity with previously solved structures of GPCRs from different families. Unique receptor sequences for 826 GPCRs were derived from Uniprot, and 3D structural models were generated for each receptor’s 7TM domain with the automated ICM Build Model tool (Abagyan et al. 2015) using alignment with the closest homology template (Katritch, 2013). Structure-based positional Ballesteros-Weinstein (BW) numbers were assigned from the structural alignments with the templates as described in GPCRDB (Isberg et al., 2015).

The N-terminal truncation sites were designed using predicted structural features in the receptor’s N-termini derived from the corresponding structural templates. For those cases where the N-terminus included important structural elements that were resolved in the 3D template, the truncation site was designed upstream of this structural element. Thus, for Secretin family GPCRs, the N-termini were truncated at the first residues attributed to their 7TM domains (Siu et al., 2013). For chemokine and other Rhodopsin family receptors, which have the N-terminal Cysteine residues predicted to make an important disulfide bond to a Cysteine in ECL3, this prospective disulfide bond was included in the construct (Wu et al., 2010; Hanson et al., 2012). Otherwise, for Rhodopsin family receptors that had a missing or truncated N-terminus in their closest structural template, we used a default truncation upstream of the beginning of helix I at BW position 1.19.

The C-terminal truncation was universally applied at BW position 7.78, which in most receptors corresponds to the site ~10 residues after the end of helix VIII. The constructs thus include potential Cysteine palmitoylation sites in helix VIII residues, when present.

The N-terminal BRIL fusion (Nt_BRIL) constructs placed the BRIL sequence at the truncated position of the receptor N-terminus as described above.

The ICL3 BRIL insertion (ICL3_BRIL) constructs were designed based on truncated sequences using insertion sites in ICL3 as in the construct that was used to solve the crystal structure of 5HT_2B_ (Wacker et al., 2013). According to this design, the BRIL sequence was inserted between BW positions 5.69 and 6.25, replacing ICL3 residues between these positions. In some rare cases when helices V and VI were shorter than in the template, additional residues from ICL3 were added to keep the helical structure in helices V and VI the same as in the 5HT_2B_ construct.

### Plasmid constructs

Gene synthesis and codon optimization was performed by GeneScript. The method of overlap extension PCR cloning was used to subclone the protein gene into the vector which is a simple and reliable way to create recombinant plasmids. The expression vector, designated as pFastBac 1, was a modified vector (Invitrogen) containing an expression cassette with a *Bam*HI flanked HA signal sequence followed by a FLAG tag at the N-terminal and with a 10× His tag at the C-terminal. Once the recombinant donor plasmids were obtained, the cloning core transfected them to the competent DH10Bac *E. coli* cells which contain bacmid and helper to facilitate the combination of the donor and bacmid into a recombinant bacmid.

### Cell culture and transfection

BV (baculovirus) expression is a high throughput platform supporting biomass production for GPCR structure and function studies. The platform transfects the insects cells (*Sf9*) with the recombinant bacmids provided by the cloning core to produce recombinant baculovirus. Recombinant baculoviruses have been widely used as vectors to express heterologous genes in cultured insect cells. High-titer recombinant baculovirus (>10^8^ viral particles per mL) was obtained using the Bac-to-Bac Baculovirus Expression System (Invitrogen). Forty mL cells were harvested by centrifugation and stored at −80°C until use.

### Quantitation of protein expression

The monoclonal ANTI-FLAG^®^M2-FITC (Sigma-Aldrich: F4049), which is a monoclonal antibody covalently conjugated to fluorescein isothiocyanate (FITC), can recognize the FLAG sequence at the N-terminus (Hanson et al., 2007). Therefore, α-flag FITC (2.5 µg/mL) was added to cells to quantify the percentage of cells with surface-expressing GPCRs and the density (mean fluorescence intensity; MFI) of GPCRs on the surface of those cells. α-Flag FITC (2.5 µg/mL) with 1.5% Triton was added to cells to quantify the total expression levels which includes total percentage and total density. For total and surface FITC expression assay, we used 10 µL FITC with and without Triton working solution plus 10 µL of cells, incubate at 4°C for 20 min, add 180 µL 1× TBS (straight TBS, without BSA), then ran the assay on a Guava flow cytometer. The Guava Express Plus GRN histogram statistics provide the count, cells/mL, mean signal intensity, and %CV for each population within a marker. Additionally, the % of total shows the percentage of the data displayed in that plot. Here, we use the data of mean signal intensity and % of total and surface expression.

### Statistical analysis

 The data was analyzed by the software of Statistical Product and Service Solution (SPSS) which can be used to do correlation analysis and cluster analysis. Through the K-S test by SPSS, most of the indexes indicated the expression levels in this study conform to a skewed distribution. The expression data distribution was analyzed by GraphPad Prism.

## Electronic supplementary material

Below is the link to the electronic supplementary material.

Supplementary material 1 (PDF 1131 kb)
