# Supplementary material for: In vitro expression and analysis of the 826 human G protein-coupled receptors
Source: Protein Cell. 2016 Apr 16;7(5):325–37. doi: 10.1007/s13238-016-0263-8 (PMC4853319; doi:10.1007/s13238-016-0263-8)
Supplement: Supplementary file 1 — Supplementary material 1 (PDF 1131 kb) [file 13238_2016_263_MOESM1_ESM.pdf]

Table S1. Comprehensive Protein Expression Data for 826 GPCRs

Family	Gene_names	Protein_names	Target name	WT-aa-count	Nt_BRIL constructs surface(%)	Nt_BRIL constructs surface density	Nt_BRIL constructs total(%)	Nt_BRIL constructs total density	ICL3_BRIL constructs surface(%)	ICL3_BRIL constructs surface density	ICL3_BRIL constructs total(%)	ICL3_BRIL constructs total density
Rho-NonO	HTR1A ADRB2RL	5-hydroxytryptamine receptor 1	5HT1A	422	53.85	50.98	96.15	288.23	80.65	64.56	96.30	504.39
**Rho-NonO**	**HTR1B HTR1DB**	**5-hydroxytryptamine receptor 1**	**5HT1B**	**390**	**64.65**	**64.60**	**94.45**	**256.41**	**67.25**	**29.44**	**80.75**	**253.35**
Rho-NonO	HTR1D HTR1DA	5-hydroxytryptamine receptor 1	5HT1D	377	54.05	41.95	93.75	242.30	72.55	38.74	79.90	273.22
Rho-NonO	HTR1E	5-hydroxytryptamine receptor 1	5HT1E	365	64.20	49.92	96.95	319.57	74.95	51.43	95.15	408.59
Rho-NonO	HTR1F HTR1EL	5-hydroxytryptamine receptor 1	5HT1F	366	43.90	67.69	84.45	93.13	46.15	63.77	94.30	489.48
Rho-NonO	HTR2A HTR2	5-hydroxytryptamine receptor 2	5HT2A	471	74.85	65.35	96.75	493.59	68.55	36.80	92.90	433.54
**Rho-NonO**	**HTR2B**	**5-hydroxytryptamine receptor 2**	**5HT2B**	**481**	**66.10**	**46.45**	**95.15**	**375.80**	**74.20**	**41.40**	**96.80**	**406.14**
Rho-NonO	HTR2C HTR1C	5-hydroxytryptamine receptor 2	5HT2C	458	62.70	26.22	96.85	171.23	96.65	152.69	94.15	703.46
Rho-NonO	HTR4	5-hydroxytryptamine receptor 4	5HT4R	388	93.50	78.29	89.70	65.34	94.10	59.69	96.70	455.89
Rho-NonO	HTR5A	5-hydroxytryptamine receptor 5	5HT5A	357	41.70	73.53	40.90	58.56	79.30	50.04	94.95	356.96
Rho-NonO	HTR6	5-hydroxytryptamine receptor 6	5HT6R	440	49.60	41.86	43.10	47.81	83.60	42.14	94.25	384.66
Rho-NonO	HTR7	5-hydroxytryptamine receptor 7	5HT7R	479	37.00	41.60	21.35	37.61	84.45	45.66	96.45	474.48
Rho-NonO	ADORA1	Adenosine receptor A1	AA1R	326	30.00	140.77	91.40	446.73	31.80	43.78	86.75	265.93
**Rho-NonO**	**ADORA2A ADO**	**Adenosine receptor A2a**	**AA2AR**	**412**	**96.20**	**184.82**	**81.20**	**211.41**	**91.45**	**142.46**	**77.90**	**286.44**
Rho-NonO	ADORA2B	Adenosine receptor A2b	AA2BR	332	31.70	115.30	41.15	483.40	23.80	30.81	79.15	110.84
Rho-NonO	ADORA3 UNQ19	Adenosine receptor A3	AA3R	318	37.95	109.19	92.15	411.35	61.45	55.75	82.75	303.75
Rho-NonO	CHRM1	Muscarinic acetylcholine recepto	ACM1	460	15.40	89.98	23.80	460.58	83.05	43.12	23.50	227.02
**Rho-NonO**	**CHRM2**	**Muscarinic acetylcholine recept**	**ACM2**	**466**	**62.05**	**51.88**	**90.90**	**539.14**	**72.95**	**38.05**	**75.60**	**268.31**
**Rho-NonO**	**CHRM3**	**Muscarinic acetylcholine recept**	**ACM3**	**590**	**50.65**	**56.72**	**76.35**	**611.62**	**46.50**	**45.90**	**78.45**	**303.67**
Rho-NonO	CHRM4	Muscarinic acetylcholine recepto	ACM4	479	34.05	47.62	87.50	534.25	34.70	95.04	79.80	352.52
Rho-NonO	CHRM5	Muscarinic acetylcholine recepto	ACM5	532	39.45	55.07	73.95	495.26	88.05	74.31	79.00	402.79
Rho-NonO	MC2R ACTHR	Adrenocorticotropic hormone re	ACTHR	297	32.90	163.80	90.00	450.20	37.30	84.49	81.35	467.05
Rho-NonO	ADRA1A ADRA1	Alpha-1A adrenergic receptor (Al	ADA1A	466	71.60	52.31	97.25	404.13	83.75	43.50	94.70	462.76
Rho-NonO	ADRA1B	Alpha-1B adrenergic receptor (Al	ADA1B	520	21.95	56.96	12.85	59.27	57.85	40.12	94.25	481.30
Rho-NonO	ADRA1D ADRA1	Alpha-1D adrenergic receptor (Al	ADA1D	572	69.45	33.20	97.05	191.87	79.80	29.33	96.95	378.08
Rho-NonO	ADRA2A ADRA2	Alpha-2A adrenergic receptor (Al	ADA2A	450	35.45	37.21	95.50	455.67	66.85	68.74	90.25	474.20
Rho-NonO	ADRA2B ADRA2	Alpha-2B adrenergic receptor (Al	ADA2B	450	89.65	151.49	84.40	440.48	37.15	48.63	90.05	518.12
Rho-NonO	ADRA2C ADRA2	Alpha-2C adrenergic receptor (Al	ADA2C	462	24.55	54.51	91.45	488.87	45.85	54.87	92.60	536.48
**Rho-NonO**	**ADRB1 ADRB1R**	**Beta-1 adrenergic receptor (Beta**	**ADRB1**	**477**	**79.35**	**55.61**	**97.95**	**465.69**	**93.30**	**70.93**	**94.90**	**299.99**
**Rho-NonO**	**ADRB2 ADRB2R**	**Beta-2 adrenergic receptor (Beta**	**ADRB2**	**413**	**42.50**	**199.68**	**97.45**	**480.55**	**71.10**	**47.83**	**95.35**	**380.84**
Rho-NonO	ADRB3 ADRB3R	Beta-3 adrenergic receptor (Beta	ADRB3	408	40.20	54.57	56.60	58.21	80.60	50.44	94.50	515.67
**Rho-NonO**	**AGTR1 AGTR1A**	**Type-1 angiotensin II receptor (**	**AGTR1**	**359**	**75.50**	**67.27**	**95.20**	**103.98**	**84.45**	**51.22**	**98.50**	**331.40**
Rho-NonO	AGTR2	Type-2 angiotensin II receptor (A	AGTR2	363	73.85	65.36	96.00	209.27	53.85	29.51	68.65	51.38
Rho-NonO	APLNR AGTRL1	Apelin receptor (Angiotensin rec	APJ	380	84.55	124.83	93.50	249.72	88.30	88.01	84.30	421.49
Rho-NonO	BDKRB1 BRADY	B1 bradykinin receptor (B1R) (BK	BKRB1	353	59.25	76.34	97.50	478.45	58.30	59.26	94.20	356.84
Rho-NonO	BDKRB2 BKR2	B2 bradykinin receptor (B2R) (BK	BKRB2	391	33.55	75.40	41.05	67.31	43.00	52.25	88.70	195.53
Rho-NonO	BRS3	Bombesin receptor subtype-3 (B	BRS3	399	48.35	21.48	6.70	37.36	95.90	64.81	95.65	664.52
Rho-NonO	C3AR1 AZ3B C3	C3a anaphylatoxin chemotactic r	C3AR	482	51.65	68.09	58.45	68.19	16.05	45.66	88.40	176.82
Rho-NonO	C5AR1 C5AR C5	C5a anaphylatoxin chemotactic r	C5AR	350	49.45	204.92	86.15	173.86	82.05	76.25	94.15	423.94

Table S1. Comprehensive Protein Expression Data for 826 GPCRs

					Nt_BRIL constructs				ICL3_BRIL constructs			
Family	Gene_names	Protein_names	Target name	WT-aa-count	surface(%)	surface density	total(%)	total density	surface(%)	surface density	total(%)	total density
Rho-NonO	GPR77 C5L2	C5a anaphylatoxin chemotactic r	C5ARL	337	50.80	41.87	59.70	48.54	43.05	106.14	79.50	131.22
Rho-NonO	CCBP2 CCR10 CM	Chemokine-binding protein 2 (C-	CCBP2	384	52.10	27.03	79.60	59.44	37.00	32.97	90.55	199.36
Rho-NonO	CCKAR CCKRA	Cholecystokinin receptor type A	CCKAR	428	47.20	30.61	54.40	39.31	64.65	44.63	93.25	669.55
Rho-NonO	CCR1 CMKBR1 C	C-C chemokine receptor type 1 (	CCR1	355	49.95	152.79	96.15	948.77	36.30	62.03	91.75	661.19
Rho-NonO	CCR10 GPR2	C-C chemokine receptor type 10	CCR10	362	39.75	94.89	91.75	497.43	66.60	143.68	92.35	1,457.21
Rho-NonO	CCR2 CMKBR2	C-C chemokine receptor type 2 (	CCR2	374	57.50	98.02	92.90	183.37	29.30	55.70	92.40	523.45
Rho-NonO	CCR3 CMKBR3	C-C chemokine receptor type 3 (	CCR3	355	36.25	99.01	91.65	423.88	31.85	56.55	91.95	378.11
Rho-NonO	CCR4 CMKBR4	C-C chemokine receptor type 4 (	CCR4	360	38.55	73.60	78.40	178.57	25.90	57.37	83.45	269.39
**Rho-NonO**	**CCR5 CMKBR5**	**C-C chemokine receptor type 5 (**	**CCR5**	**352**	**53.80**	**39.57**	**87.10**	**206.88**	**54.80**	**42.41**	**90.60**	**719.78**
Rho-NonO	CCR6 CKRL3 CM	C-C chemokine receptor type 6 (	CCR6	374	50.65	58.37	96.95	266.20	35.25	66.08	90.10	338.70
Rho-NonO	CCR7 CMKBR7 E	C-C chemokine receptor type 7 (	CCR7	378	59.45	131.40	95.30	358.20	80.15	72.53	85.70	577.91
Rho-NonO	CCR8 CKRL1 CM	C-C chemokine receptor type 8 (	CCR8	355	62.80	68.07	88.15	163.24	68.65	32.26	63.50	166.59
Rho-NonO	CCR9 GPR28	C-C chemokine receptor type 9 (	CCR9	369	29.70	165.30	86.95	383.35	60.80	45.84	73.30	419.05
Rho-NonO	CCRL1 CCBP2 CC	C-C chemokine receptor type 11	CCRL1	350	65.50	30.10	98.75	428.67	75.20	20.36	95.05	280.37
Rho-NonO	CCRL2 CCR11 CC	C-C chemokine receptor-like 2 (C	CCRL2	344	64.15	66.81	98.20	678.50	67.95	39.97	95.75	420.05
Rho-NonO	CYSLTR1 CYSLT1	Cysteinyl leukotriene receptor 1	CLTR1	337	58.50	80.71	97.50	531.50	38.10	54.89	90.30	446.04
Rho-NonO	CYSLTR2 CYSLT2	Cysteinyl leukotriene receptor 2	CLTR2	346	42.30	35.94	65.25	45.82	76.65	28.05	92.10	224.49
Rho-NonO	CMKLR1 CHEMR	Chemokine-like receptor 1 (G-pro	CML1	373	82.80	50.80	93.25	88.44	37.35	32.94	45.35	34.94
Rho-NonO	CNR1 CNR	Cannabinoid receptor 1 (CB-R) (C	CNR1	472	20.00	73.39	53.05	242.89	63.15	46.63	87.60	240.68
Rho-NonO	CNR2	Cannabinoid receptor 2 (CB-2) (C	CNR2	360	47.15	57.47	88.45	230.52	45.85	39.53	78.05	237.97
Rho-NonO	CX3CR1 CMKBRI	CX3C chemokine receptor 1 (C-X3	CX3C1	355	45.70	38.40	76.30	75.44	44.25	43.72	94.50	258.51
Rho-NonO	CXCR1 CMKAR1	C-X-C chemokine receptor type 1	CXCR1	350	53.75	32.20	74.20	51.06	82.10	36.07	94.85	311.31
Rho-NonO	CXCR2 IL8RB	C-X-C chemokine receptor type 2	CXCR2	360	63.10	50.45	96.65	575.27	82.50	39.81	94.70	367.79
Rho-NonO	CXCR3 GPR9	C-X-C chemokine receptor type 3	CXCR3	368	39.85	58.11	89.45	101.61	75.60	50.20	95.65	426.26
**Rho-NonO**	**CXCR4**	**C-X-C chemokine receptor type 4**	**CXCR4**	**352**	**50.55**	**52.06**	**79.20**	**86.18**	**44.35**	**151.25**	**95.75**	**639.41**
Rho-NonO	CXCR5 BLR1 MD	C-X-C chemokine receptor type 5	CXCR5	372	43.00	30.28	67.80	50.86	48.30	82.98	96.30	623.18
Rho-NonO	CXCR6 BONZO S	C-X-C chemokine receptor type 6	CXCR6	342	50.75	26.02	61.00	44.82	52.05	144.11	88.35	562.63
Rho-NonO	CXCR7 CMKOR1	C-X-C chemokine receptor type 7	CXCR7	362	45.80	27.72	61.15	52.46	38.50	38.99	90.65	279.88
Rho-NonO	DRD1	D(1A) dopamine receptor (Dopar	DRD1	446	41.70	75.19	71.45	265.64	37.95	44.02	83.55	376.90
Rho-NonO	DRD2	D(2) dopamine receptor (Dopam	DRD2	443	31.60	40.77	68.50	203.86	75.05	66.53	69.60	345.61
**Rho-NonO**	**DRD3**	**D(3) dopamine receptor (Dopam**	**DRD3**	**400**	**66.70**	**49.86**	**83.65**	**311.63**	**63.50**	**48.89**	**72.45**	**254.33**
Rho-NonO	DRD4	D(4) dopamine receptor (D(2C) d	DRD4	467	76.10	86.21	80.95	378.23	79.60	187.29	81.65	405.73
Rho-NonO	DRD5 DRD1B DR	D(1B) dopamine receptor (D(5) d	DRD5	477	15.50	175.16	52.90	216.06	19.95	74.24	79.70	367.41
Rho-NonO	DARC FY GPD	Duffy antigen/chemokine recept	DUFFY	336	68.50	70.52	96.90	338.30	92.25	259.14	86.55	1,051.32
Rho-NonO	EDNRA ETA ETR/	Endothelin-1 receptor (Endotheli	EDNRA	427	82.50	143.31	97.90	487.91	56.35	63.07	73.60	88.60
Rho-NonO	EDNRB ETRB	Endothelin B receptor (ET-B) (ET-	EDNRB	442	71.75	30.67	78.30	54.86	96.00	200.37	93.65	602.24
Rho-NonO	GPR37L1 ETBRLI	Endothelin B receptor-like protei	ETBR2	481	92.90	68.07	59.70	53.97	76.30	32.98	96.10	104.00
**Rho-NonO**	**FFAR1 GPR40**	**Free fatty acid receptor 1 (G-pro**	**FFAR1**	**300**	**68.65**	**175.22**	**97.55**	**1,034.22**	**27.20**	**202.63**	**40.55**	**98.88**
Rho-NonO	FFAR2 GPR43	Free fatty acid receptor 2 (G-prot	FFAR2	330	52.10	55.18	68.00	75.53	26.00	27.23	20.80	25.58
Rho-NonO	FFAR3 GPR41	Free fatty acid receptor 3 (G-prot	FFAR3	346	71.70	215.18	96.35	246.62	20.80	47.08	34.85	34.01

Table S1. Comprehensive Protein Expression Data for 826 GPCRs

						Nt_BRIL constructs					ICL3_BRIL constructs			
Family	Gene_names	Protein_names	Target name	WT-aa-count		surface(%)	surface density	total(%)	total density		surface(%)	surface density	total(%)	total density
Rho-NonO	FFAR4 GPR120 (	Free fatty acid receptor 4 (G-prot	FFAR4	377		52.90	120.67	94.00	237.25		25.40	36.72	36.80	34.90
Rho-NonO	FPR1	fMet-Leu-Phe receptor (fMLP rec	FPR1	350		65.40	86.28	92.35	172.46		72.65	46.84	97.95	263.59
Rho-NonO	FPR2 FPRH1 FPR	N-formyl peptide receptor 2 (FM	FPR2	351		65.95	76.60	94.40	111.69		47.60	37.96	75.70	60.65
Rho-NonO	FPR3 FPRH1 FPR	N-formyl peptide receptor 3 (FM	FPR3	353		40.25	75.14	74.80	89.82		63.25	44.58	91.90	387.28
Rho-NonO	FSHR LGR1	Follicle-stimulating hormone rec	FSHR	695		33.85	63.67	91.65	287.42		13.60	104.37	21.90	50.08
Rho-NonO	GALR1 GALNR G	Galanin receptor type 1 (GAL1-R)	GALR1	349		53.25	29.72	71.60	52.67		70.40	42.27	92.85	460.29
Rho-NonO	GALR2 GALNR2	Galanin receptor type 2 (GAL2-R)	GALR2	387		57.45	23.61	73.55	59.39		80.80	30.56	97.20	233.94
Rho-NonO	GALR3 GALNR3	Galanin receptor type 3 (GAL3-R)	GALR3	368		49.90	34.45	56.40	42.90		78.50	38.57	95.95	245.76
Rho-NonO	CCKBR CCKRB	Gastrin/cholecystokinin type B re	GASR	447		70.65	72.48	94.45	114.33		65.45	53.77	80.10	85.06
Rho-NonO	GHSR	Growth hormone secretagogue r	GHSR	366		43.90	113.14	96.90	616.74		68.45	33.92	92.75	189.21
Rho-NonO	GNRHR GRHR	Gonadotropin-releasing hormon	GNRHR	328		32.65	54.20	35.90	51.82		53.65	66.68	86.05	312.53
Rho-NonO	GNRHR2	Putative gonadotropin-releasing	GNRR2	292		1.85	26.50	20.60	29.47		50.10	97.92	93.25	1,039.43
Rho-NonO	GPR101	Probable G-protein coupled rece	GP101	508		83.05	84.24	87.20	88.21		37.75	61.95	61.75	55.42
Rho-NonO	GPR119	Glucose-dependent insulinotropi	GP119	335		37.20	65.82	69.35	538.83		48.50	26.55	54.90	112.80
Rho-NonO	GPR132 G2A	Probable G-protein coupled rece	GP132	380		17.95	84.19	46.35	93.07		19.85	47.91	92.95	130.14
Rho-NonO	GPR135	Probable G-protein coupled rece	GP135	494		65.80	77.97	94.70	284.17		83.30	284.71	93.35	1,084.53
Rho-NonO	GPR139 GPRG1	Probable G-protein coupled rece	GP139	353		31.20	48.06	44.05	48.11		18.35	23.87	64.05	89.41
Rho-NonO	GPR141 PGR13	Probable G-protein coupled rece	GP141	305		52.70	47.59	83.00	79.58		56.75	63.20	95.75	633.83
Rho-NonO	GPR142 PGR2	Probable G-protein coupled rece	GP142	462		45.15	47.33	97.10	198.29		63.20	73.94	71.25	56.21
Rho-NonO	GPR143 OA1	G-protein coupled receptor 143 (	GP143	404		48.75	135.76	85.15	166.42		50.20	272.68	93.65	578.86
Rho-NonO	GPR146 PGR8	Probable G-protein coupled rece	GP146	333		46.25	201.66	52.65	130.15		38.15	67.63	89.60	313.37
Rho-NonO	GPR148 BTR PGI	Probable G-protein coupled rece	GP148	347		45.60	89.45	49.00	84.96		43.55	58.51	95.60	394.09
Rho-NonO	GPR149 PGR10	Probable G-protein coupled rece	GP149	731		37.95	124.36	94.65	384.70		72.80	65.34	95.20	843.96
Rho-NonO	GPR150	Probable G-protein coupled rece	GP150	434		44.35	90.98	96.10	380.82		59.15	68.61	93.45	279.06
Rho-NonO	GPR151 PGR7	Probable G-protein coupled rece	GP151	419		58.10	44.65	89.85	113.53		49.50	46.66	81.20	97.60
Rho-NonO	GPR152 PGR5	Probable G-protein coupled rece	GP152	470		51.00	191.76	61.60	137.07		52.25	41.82	85.55	103.82
Rho-NonO	GPR153 PGR1	Probable G-protein coupled rece	GP153	609		56.80	84.62	88.30	212.56		85.80	38.57	86.65	231.40
Rho-NonO	GPR160 GPCR15	Probable G-protein coupled rece	GP160	338		41.60	156.26	46.20	83.99		61.45	47.64	93.05	510.41
Rho-NonO	GPR161	G-protein coupled receptor 161 (	GP161	529		79.40	77.67	96.65	312.19		55.10	57.38	94.85	304.97
Rho-NonO	GPR162 GRCA	Probable G-protein coupled rece	GP162	588		46.80	48.14	86.95	164.01		81.20	37.48	91.20	749.73
Rho-NonO	GPR171 H963	Probable G-protein coupled rece	GP171	319		44.35	115.96	67.50	123.89		45.45	46.29	95.25	536.53
Rho-NonO	GPR173 SREB3	Probable G-protein coupled rece	GP173	373		55.45	64.88	44.90	39.00		81.95	38.60	93.75	283.42
Rho-NonO	GPR174 FKSG79	Probable G-protein coupled rece	GP174	333		42.80	110.95	79.25	121.37		53.40	55.34	93.95	241.15
Rho-NonO	GPR176	Probable G-protein coupled rece	GP176	515		28.25	85.06	82.20	536.56		33.79	38.04	10.07	72.26
Rho-NonO	GPR182 ADMR	G-protein coupled receptor 182	GP182	404		57.80	132.08	98.15	611.55		42.15	47.08	87.55	183.79
Rho-NonO	GPR183 EBI2	G-protein coupled receptor 183 (	GP183	361		46.80	32.72	52.95	35.12		42.40	48.18	93.55	351.97
Rho-NonO	GPBAR1 TGR5	G-protein coupled bile acid recep	GPBAR	330		57.75	84.57	91.30	290.50		88.15	74.76	83.75	321.23
Rho-NonO		Putative G-protein coupled recep	GPC39	272		81.55	84.91	97.75	536.43		53.55	58.69	77.10	85.46
Rho-NonO	GPER CEPR CMK	G-protein coupled estrogen rece	GPER	375		41.25	36.62	56.50	47.87		46.70	126.32	97.10	601.99
Rho-NonO	GPR1	G-protein coupled receptor 1	GPR1	355		21.60	93.71	63.25	240.47		52.75	41.51	73.05	73.39

Table S1. Comprehensive Protein Expression Data for 826 GPCRs

						Nt_BRIL constructs					ICL3_BRIL constructs			
Family	Gene_names	Protein_names	Target name	WT-aa-count		surface(%)	surface density	total(%)	total density		surface(%)	surface density	total(%)	total density
Rho-NonO	GPR12	G-protein coupled receptor 12	GPR12	334		45.40	26.87	56.95	35.00		22.45	181.06	61.55	432.79
Rho-NonO	GPR15	G-protein coupled receptor 15 (E	GPR15	360		70.90	70.26	86.35	80.80		70.85	59.38	95.10	434.11
Rho-NonO	GPR17	Uracil nucleotide/cysteinyl leuko	GPR17	367		50.95	41.65	82.90	71.86		60.50	45.84	68.95	53.87
Rho-NonO	GPR18 GPCRW	N-arachidonyl glycine receptor (N	GPR18	331		67.85	67.07	96.70	495.52		67.05	32.28	95.65	445.66
Rho-NonO	GPR19	Probable G-protein coupled rece	GPR19	415		70.65	78.25	93.60	97.69		46.10	49.72	95.80	377.15
Rho-NonO	GPR20	G-protein coupled receptor 20	GPR20	358		46.95	43.48	71.45	54.59		49.70	54.43	96.00	550.54
Rho-NonO	GPR21	Probable G-protein coupled rece	GPR21	349		63.40	89.69	81.80	441.82		34.25	23.91	57.40	48.31
Rho-NonO	GPR22	Probable G-protein coupled rece	GPR22	433		61.20	30.06	57.45	35.13		75.15	31.83	87.30	241.44
Rho-NonO	GPR25	Probable G-protein coupled rece	GPR25	361		31.85	81.30	56.70	94.02		53.85	42.73	93.55	489.07
Rho-NonO	GPR26	G-protein coupled receptor 26	GPR26	337		83.65	85.55	97.70	414.86		46.05	50.44	51.05	63.55
Rho-NonO	GPR27 SREB1	Probable G-protein coupled rece	GPR27	375		66.05	39.78	95.15	244.86		71.15	33.82	96.45	167.40
Rho-NonO	GPR3 ACCA	G-protein coupled receptor 3 (AC	GPR3	330		78.30	122.75	97.90	700.87		8.05	71.91	3.05	33.61
Rho-NonO	GPR31	12-(S)-hydroxy-5,8,10,14-eicosat	GPR31	319		68.90	57.98	92.45	72.12		52.45	111.26	98.45	439.11
Rho-NonO	GPR32	Probable G-protein coupled rece	GPR32	356		89.55	59.69	94.85	117.80		30.75	38.28	55.35	56.59
Rho-NonO	GPR33	Probable G-protein coupled rece	GPR33	333		76.90	38.22	97.85	274.40		69.40	129.90	83.15	176.40
Rho-NonO	GPR34	Probable G-protein coupled rece	GPR34	381		39.35	83.31	54.80	96.08		68.65	33.10	94.55	560.54
Rho-NonO	GPR35	G-protein coupled receptor 35 (K	GPR35	309		55.50	41.41	78.15	400.54		51.60	52.43	93.85	501.27
Rho-NonO	GPR37	Probable G-protein coupled rece	GPR37	613		66.45	112.63	98.10	909.40		31.75	19.87	75.95	152.88
Rho-NonO	GPR39	G-protein coupled receptor 39	GPR39	453		68.15	93.87	73.85	283.96		57.65	45.66	92.80	513.02
Rho-NonO	GPR4 GPR19	G-protein coupled receptor 4 (G-	GPR4	362		47.85	184.14	96.85	642.40		29.70	43.97	91.85	249.51
Rho-NonO	GPR42 GPR42P	G-protein coupled receptor 42	GPR42	346		33.75	108.75	61.10	137.01		30.85	46.76	88.90	311.73
Rho-NonO	GPR45	Probable G-protein coupled rece	GPR45	372		23.80	60.69	60.70	85.77		30.45	32.76	90.45	160.66
Rho-NonO	GPR52	Probable G-protein coupled rece	GPR52	361		78.65	95.46	98.00	526.15		46.95	50.75	97.95	215.58
Rho-NonO	GPR55	G-protein coupled receptor 55	GPR55	319		43.55	116.97	97.70	831.15		38.65	33.57	94.25	424.46
Rho-NonO	GPR6	G-protein coupled receptor 6 (Sp	GPR6	362		17.49	97.84	59.85	125.31		43.80	179.85	82.05	813.91
Rho-NonO	GPR61 BALGR	Probable G-protein coupled rece	GPR61	451		15.85	47.26	25.25	45.17		50.60	30.53	92.70	256.64
Rho-NonO	GPR62	Probable G-protein coupled rece	GPR62	368		61.55	40.14	96.80	403.00		49.10	126.65	94.95	974.51
Rho-NonO	GPR63 PSP24B	Probable G-protein coupled rece	GPR63	419		67.50	58.04	93.15	460.95		59.15	33.00	92.80	216.27
Rho-NonO	GPR75	Probable G-protein coupled rece	GPR75	540		19.20	85.92	21.35	53.69		60.65	38.11	95.05	395.18
Rho-NonO	GPR78 UNQ592	G-protein coupled receptor 78	GPR78	363		53.00	67.20	58.55	44.94		53.95	14.54	38.30	26.94
Rho-NonO	GPR82	Probable G-protein coupled rece	GPR82	336		82.75	91.45	96.60	382.24		26.90	51.58	52.20	83.33
Rho-NonO	GPR83 GPR72 KI	Probable G-protein coupled rece	GPR83	423		58.90	31.72	77.10	38.94		83.05	36.32	95.25	221.01
Rho-NonO	GPR84 EX33	G-protein coupled receptor 84 (I	GPR84	396		53.00	63.67	61.45	52.67		51.55	44.87	51.85	39.75
Rho-NonO	GPR85 SREB2	Probable G-protein coupled rece	GPR85	370		90.20	65.28	97.65	529.95		75.70	46.62	84.55	289.26
Rho-NonO	GPR87 GPR95 FI	G-protein coupled receptor 87 (G	GPR87	358		62.00	20.31	62.55	38.09		86.20	29.20	94.70	549.53
Rho-NonO	GPR88 STRG	Probable G-protein coupled rece	GPR88	384		66.25	53.39	96.10	700.64		75.40	145.85	91.50	583.08
Rho-NonO	GRPR	Gastrin-releasing peptide recept	GRPR	384		45.85	87.18	51.30	78.03		75.40	97.10	95.00	440.07
Rho-NonO	HCAR1 GPR104 (	Hydroxycarboxylic acid receptor	HCAR1	346		43.75	97.67	83.70	83.70		24.20	61.55	89.35	370.94
Rho-NonO	HCAR2 GPR109A	Hydroxycarboxylic acid receptor	HCAR2	363		57.50	72.64	78.25	78.25		57.25	59.26	94.50	371.98
Rho-NonO	HCAR3 GPR109B	Hydroxycarboxylic acid receptor	HCAR3	387		41.15	57.43	67.80	67.80		63.65	84.79	95.85	587.19

Table S1. Comprehensive Protein Expression Data for 826 GPCRs

					Nt_BRIL constructs				ICL3_BRIL constructs			
Family	Gene_names	Protein_names	Target name	WT-aa-count	surface(%)	surface density	total(%)	total density	surface(%)	surface density	total(%)	total density
Rho-NonO	HRH1	Histamine H1 receptor (H1R) (HI	HRH1	487	43.00	20.11	73.60	56.82	82.00	33.82	95.05	399.93
Rho-NonO	HRH2	Histamine H2 receptor (H2R) (HH	HRH2	359	49.60	32.51	72.75	60.00	39.40	67.02	94.90	597.47
Rho-NonO	HRH3 GPCR97	Histamine H3 receptor (H3R) (HH	HRH3	445	61.10	61.01	95.75	381.19	54.55	61.73	90.00	292.55
Rho-NonO	HRH4 GPCR105	Histamine H4 receptor (H4R) (HH	HRH4	390	42.35	84.17	80.05	80.05	84.30	48.45	90.75	257.30
Rho-NonO	KISS1R AXOR12	KiSS-1 receptor (KiSS-1R) (G-prot	KISSR	398	42.75	46.33	84.75	64.22	56.15	57.70	94.45	458.35
Rho-NonO	LGR4 GPR48	Leucine-rich repeat-containing G	LGR4	951	93.70	234.89	85.20	434.50	37.05	55.40	83.25	733.21
Rho-NonO	LGR5 GPR49 GPI	Leucine-rich repeat-containing G	LGR5	907	50.95	115.34	91.10	251.50	33.05	66.82	73.95	487.35
Rho-NonO	LGR6 UNQ6427/	Leucine-rich repeat-containing G	LGR6	967	79.90	142.22	58.20	463.09	31.65	31.97	73.10	337.13
Rho-NonO	LPAR1 EDG2 LPA	Lysophosphatidic acid receptor 1	LPAR1	364	9.40	18.51	15.02	18.82	87.05	57.72	84.10	284.37
Rho-NonO	LPAR2 EDG4 LPA	Lysophosphatidic acid receptor 2	LPAR2	351	40.75	133.75	93.35	521.12	37.50	44.62	98.20	1,194.63
Rho-NonO	LPAR3 EDG7 LPA	Lysophosphatidic acid receptor 3	LPAR3	353	53.85	30.05	87.50	271.47	63.00	37.70	93.15	396.08
Rho-NonO	LPAR4 GPR23 LP	Lysophosphatidic acid receptor 4	LPAR4	370	36.05	67.86	69.85	87.02	40.55	60.18	95.25	778.37
Rho-NonO	LPAR5 GPR92 GI	Lysophosphatidic acid receptor 5	LPAR5	372	46.20	51.83	76.70	81.46	53.10	72.23	95.70	461.89
Rho-NonO	LPAR6 P2RY5	Lysophosphatidic acid receptor 6	LPAR6	344	35.05	45.03	68.65	73.90	32.40	26.99	92.70	295.76
Rho-NonO	LHCGR LCGR LGI	Lutropin-choriogonadotropic hor	LSHR	699	12.30	60.71	20.70	55.89	44.95	61.93	82.70	88.82
Rho-NonO	LTB4R BLT BLT1	Leukotriene B4 receptor 1 (LTB4-	LT4R1	352	68.00	38.78	84.90	47.52	63.95	26.82	80.10	61.01
Rho-NonO	LTB4R2 BLT2R B	Leukotriene B4 receptor 2 (LTB4-	LT4R2	389	77.35	123.30	97.00	555.21	62.40	79.06	76.10	106.05
Rho-NonO	MAS1 MAS	Proto-oncogene Mas	MAS	325	46.65	103.46	92.65	394.28	68.70	37.96	94.90	277.41
Rho-NonO	MAS1L MRG	Mas-related G-protein coupled r	MAS1L	378	37.20	57.35	41.00	45.77	57.15	62.82	93.30	327.09
Rho-NonO	MC3R	Melanocortin receptor 3 (MC3-R	MC3R	323	18.00	30.54	74.15	267.94	9.55	44.62	29.00	51.60
Rho-NonO	MC4R	Melanocortin receptor 4 (MC4-R	MC4R	332	55.60	41.92	60.30	116.13	18.00	30.32	80.45	233.86
Rho-NonO	MC5R	Melanocortin receptor 5 (MC5-R	MC5R	325	71.25	55.39	88.40	144.24	36.70	56.80	88.00	419.03
Rho-NonO	MCHR1 GPR24 S	Melanin-concentrating hormone	MCHR1	422	50.95	88.05	76.90	112.78	62.00	36.18	96.30	347.40
Rho-NonO	MCHR2 GPR145	Melanin-concentrating hormone	MCHR2	340	36.80	84.87	73.55	97.67	63.70	51.71	93.45	437.31
Rho-NonO	MRGPRD MRGD	Mas-related G-protein coupled r	MRGRD	321	83.10	55.31	97.85	448.57	75.25	41.20	93.85	300.54
Rho-NonO	MRGPRE GPR16	Mas-related G-protein coupled r	MRGRE	311	84.10	87.87	71.20	67.19	88.55	107.69	82.05	304.58
Rho-NonO	MRGPRF GPR14	Mas-related G-protein coupled r	MRGRF	343	82.55	60.82	98.40	303.59	62.85	67.39	92.40	269.03
Rho-NonO	MRGPRG GPR16	Mas-related G-protein coupled r	MRGRG	289	52.35	43.13	84.60	106.10	49.50	15.63	84.70	32.31
Rho-NonO	MRGPRX1 MRG	Mas-related G-protein coupled r	MRGX1	322	46.15	49.48	90.85	145.52	77.10	42.11	92.40	305.58
Rho-NonO	MRGPRX2 MRG	Mas-related G-protein coupled r	MRGX2	330	52.60	53.13	94.40	379.54	77.40	30.42	91.50	232.06
Rho-NonO	MRGPRX3 MRG	Mas-related G-protein coupled r	MRGX3	322	72.75	45.23	97.05	269.31	77.90	36.96	94.45	517.21
Rho-NonO	MRGPRX4 MRG	Mas-related G-protein coupled r	MRGX4	322	66.25	123.95	72.35	67.34	44.35	91.82	75.30	81.96
Rho-NonO	MC1R MSHR	Melanocyte-stimulating hormon	MSHR	317	41.65	115.44	89.80	312.94	51.75	58.34	70.70	211.88
Rho-NonO	MLNR GPR38 M	Motilin receptor (G-protein coup	MTLR	412	50.35	44.44	81.10	62.39	73.00	35.48	89.95	237.06
Rho-NonO	MTNR1A	Melatonin receptor type 1A (Mel	MTR1A	350	36.50	40.95	80.05	64.61	69.40	42.12	96.65	442.21
Rho-NonO	MTNR1B	Melatonin receptor type 1B (Mel	MTR1B	362	34.40	68.01	75.50	88.47	71.50	45.52	95.15	435.66
Rho-NonO	GPR50	Melatonin-related receptor (G pr	MTR1L	617	47.50	48.28	92.35	86.79	75.35	25.72	82.20	227.90
Rho-NonO	TACR1 NK1R TA(	Substance-P receptor (SPR) (NK-1	NK1R	407	76.15	53.93	94.85	111.81	53.00	25.70	74.95	56.13
Rho-NonO	TACR2 NK2R NK	Substance-K receptor (SKR) (NK-2	NK2R	398	71.35	81.47	94.40	140.94	48.15	51.39	76.60	108.29
Rho-NonO	TACR3 NK3R TA(	Neuromedin-K receptor (NKR) (N	NK3R	465	52.70	51.33	95.65	173.03	76.35	29.84	92.60	223.04

Table S1. Comprehensive Protein Expression Data for 826 GPCRs

Family	Gene_names	Protein_names	Target name	WT-aa-count		Nt_BRIL constructs surface(%)	Nt_BRIL surface density	Nt_BRIL total(%)	Nt_BRIL total density		ICL3_BRIL constructs surface(%)	ICL3_BRIL surface density	ICL3_BRIL total(%)	ICL3_BRIL total density
Rho-NonO	NMBR	Neuromedin-B receptor (NMB-R)	NMBR	390		29.00	53.98	68.30	67.76		77.30	46.03	95.00	485.58
Rho-NonO	NMUR1 GPR66	Neuromedin-U receptor 1 (NMU	NMUR1	426		74.30	20.65	52.65	25.14		29.65	63.90	88.40	364.52
Rho-NonO	NMUR2 NMU2R	Neuromedin-U receptor 2 (NMU	NMUR2	415		53.20	49.75	86.10	85.48		43.65	24.92	81.00	31.04
Rho-NonO	NPBWR1 GPR7	Neuropeptides B/W receptor typ	NPBW1	328		50.70	163.37	97.45	578.37		49.50	114.52	92.00	424.59
Rho-NonO	NPBWR2 GPR8	Neuropeptides B/W receptor typ	NPBW2	333		42.45	89.37	96.30	430.84		30.90	47.06	90.45	156.73
Rho-NonO	NPFFR1 GPR147	Neuropeptide FF receptor 1 (G-p	NPFF1	430		40.65	75.27	82.50	101.50		48.75	34.37	94.15	497.46
Rho-NonO	NPFFR2 GPR74 N	Neuropeptide FF receptor 2 (G-p	NPFF2	522		51.60	96.94	98.15	314.74		28.50	43.53	87.35	245.40
Rho-NonO	NPSR1 GPR154 (	Neuropeptide S receptor (G-prot	NPSR1	371		75.25	113.39	97.25	484.33		44.85	47.55	71.35	82.47
Rho-NonO	NPY1R NPYR NP	Neuropeptide Y receptor type 1 (	NPY1R	384		29.30	76.03	83.60	144.32		89.45	64.89	89.90	309.37
Rho-NonO	NPY2R	Neuropeptide Y receptor type 2 (	NPY2R	381		12.30	63.24	43.45	92.15		20.20	29.82	79.90	270.60
Rho-NonO	PPYR1 NPY4R	Neuropeptide Y receptor type 4 (	NPY4R	375		32.35	118.66	75.75	111.71		31.40	44.04	89.85	233.94
Rho-NonO	NPY5R NPYR5	Neuropeptide Y receptor type 5 (	NPY5R	445		25.00	123.71	40.15	71.70		65.45	52.30	97.35	496.37
Rho-NonO	NPY6R NPY1RL \	Putative neuropeptide Y recepto	NPY6R	290		66.20	75.54	94.00	200.50		55.65	24.33	97.45	141.70
**Rho-NonO**	**NTSR1 NTRR**	**Neurotensin receptor type 1 (NT**	**NTR1**	**418**		**53.30**	**189.24**	**96.65**	**618.41**		**66.60**	**172.20**	**94.75**	**793.11**
Rho-NonO	NTSR2	Neurotensin receptor type 2 (NT	NTR2	410		46.10	113.57	96.50	468.83		58.85	71.53	92.55	484.27
Rho-NonO	GPR68 OGR1	Ovarian cancer G-protein couple	OGR1	365		60.10	95.67	96.65	530.74		47.30	36.10	92.30	281.99
Rho-NonO	OPN3 ECPN	Opsin-3 (Encephalopsin) (Panops	OPN3	402		53.35	76.06	93.85	276.53		74.35	146.08	94.45	730.90
Rho-NonO	OPN4 MOP	Melanopsin (Opsin-4)	OPN4	478		45.20	82.98	84.80	123.83		71.15	107.04	91.10	456.15
Rho-NonO	OPN5 GPR136 P	Opsin-5 (G-protein coupled recep	OPN5	354		52.30	103.04	95.60	404.17		64.35	126.42	94.35	774.32
**Rho-NonO**	**OPRD1 OPRD**	**Delta-type opioid receptor (D-O**	**OPRD**	**372**		**37.75**	**44.89**	**66.05**	**68.44**		**27.10**	**86.55**	**90.25**	**249.79**
**Rho-NonO**	**OPRK1 OPRK**	**Kappa-type opioid receptor (K-C**	**OPRK**	**380**		**19.15**	**48.10**	**72.45**	**91.54**		**39.35**	**26.44**	**74.15**	**229.09**
**Rho-NonO**	**OPRM1 MOR1**	**Mu-type opioid receptor (M-OR**	**OPRM**	**400**		**35.70**	**44.88**	**79.85**	**139.98**		**48.20**	**30.27**	**55.95**	**262.07**
**Rho-NonO**	**OPRL1 OOR ORI**	**Nociceptin receptor (Kappa-type**	**OPRX**	**370**		**50.35**	**63.50**	**96.65**	**273.03**		**51.35**	**32.81**	**90.25**	**281.40**
Rho-NonO	OPN1SW BCP	Short-wave-sensitive opsin 1 (Blu	OPSB	348		89.85	92.19	93.30	800.47		91.45	55.30	86.05	1,035.60
**Rho-NonO**	**RHO OPN2**	**Rhodopsin (Opsin-2)**	**OPSD**	**348**		**97.00**	**396.31**	**61.15**	**216.61**		**94.45**	**190.03**	**39.95**	**315.71**
Rho-NonO	OPN1MW GCP;	Medium-wave-sensitive opsin 1 (	OPSG	364		93.10	97.20	95.75	177.12		95.70	227.89	84.05	308.09
Rho-NonO	OPN1LW RCP	Long-wave-sensitive opsin 1 (Rec	OPSR	364		60.60	77.81	93.00	151.03		93.90	141.82	86.80	417.43
Rho-NonO	RRH	Visual pigment-like receptor pero	OPSX	337		71.50	75.97	93.85	179.45		42.25	36.90	64.45	79.31
Rho-NonO	HCRTR1	Orexin receptor type 1 (Ox-1-R) (	OX1R	425		74.65	48.05	96.70	626.99		74.85	27.55	94.75	411.98
**Rho-NonO**	**HCRTR2**	**Orexin receptor type 2 (Ox-2-R)**	**OX2R**	**444**		**54.55**	**78.79**	**81.35**	**90.45**		**50.80**	**26.76**	**87.00**	**255.64**
Rho-NonO	OXER1 GPR170 T	Oxoeicosanoid receptor 1 (5-oxo	OXER1	423		66.95	100.37	98.10	654.73		77.65	52.04	96.45	351.30
Rho-NonO	OXGR1 GPR80 G	2-oxoglutarate receptor 1 (Alpha	OXGR1	337		37.20	80.45	37.50	73.18		69.20	29.61	94.55	303.00
Rho-NonO	OXTR	Oxytocin receptor (OT-R)	OXYR	389		79.15	80.10	98.30	414.24		74.70	36.65	83.60	56.58
**Rho-NonO**	**P2RY1**	**P2Y purinoceptor 1 (P2Y1) (ATP**	**P2RY1**	**373**		**43.85**	**52.19**	**70.75**	**76.83**		**74.40**	**26.52**	**93.25**	**190.27**
Rho-NonO	P2RY2 P2RU1	P2Y purinoceptor 2 (P2Y2) (ATP r	P2RY2	377		50.80	40.28	82.25	81.84		81.30	41.28	97.05	330.88
Rho-NonO	P2RY4 NRU	P2Y purinoceptor 4 (P2Y4) (P2P)	P2RY4	365		52.40	50.64	79.45	85.49		79.55	44.67	97.05	295.01
Rho-NonO	P2RY6 PP2891	P2Y purinoceptor 6 (P2Y6)	P2RY6	328		67.70	70.83	96.55	449.18		68.65	56.12	96.50	266.45
Rho-NonO	P2RY8	P2Y purinoceptor 8 (P2Y8)	P2RY8	359		64.60	88.67	97.95	501.51		64.25	33.29	93.15	198.99
Rho-NonO	P2RY10	Putative P2Y purinoceptor 10 (P2	P2Y10	339		14.20	117.38	38.40	136.57		60.70	24.05	95.25	303.20
Rho-NonO	P2RY11	P2Y purinoceptor 11 (P2Y11)	P2Y11	374		78.55	111.37	97.70	587.06		68.40	35.86	84.65	74.01

Table S1. Comprehensive Protein Expression Data for 826 GPCRs

Family	Gene_names	Protein_names	Target name	WT-aa-count		Nt_BRIL constructs					ICL3_BRIL constructs			
						surface(%)	surface density	total(%)	total density		surface(%)	surface density	total(%)	total density
**Rho-NonO**	**P2RY12 HORK3**	**P2Y purinoceptor 12 (P2Y12) (AI**	**P2Y12**	**342**		**40.80**	**71.45**	**89.00**	**401.70**		**51.75**	**179.70**	**88.95**	**770.32**
Rho-NonO	P2RY13 GPR86 (	P2Y purinoceptor 13 (P2Y13) (G-|	P2Y13	354		20.43	58.42	49.15	27.72		79.65	27.37	96.10	537.48
Rho-NonO	P2RY14 GPR105	P2Y purinoceptor 14 (P2Y14) (G-|	P2Y14	338		58.45	70.07	75.55	72.61		77.05	96.87	95.50	731.41
**Rho-NonO**	**F2R CF2R PAR1**	**Proteinase-activated receptor 1**	**PAR1**	**425**		**84.40**	**53.04**	**88.25**	**102.36**		**92.85**	**47.53**	**86.25**	**101.03**
Rho-NonO	F2RL1 GPR11 PA	Proteinase-activated receptor 2 (	PAR2	397		73.35	77.42	98.05	271.61		62.30	23.53	95.20	238.78
Rho-NonO	F2RL2 PAR3	Proteinase-activated receptor 3 (	PAR3	374		86.30	101.69	98.15	434.04		76.45	24.05	94.80	151.66
Rho-NonO	F2RL3 PAR4	Proteinase-activated receptor 4 (	PAR4	385		27.25	198.29	60.15	178.65		75.60	48.13	95.55	285.13
Rho-NonO	PTGDR	Prostaglandin D2 receptor (PGD	PD2R	359		77.95	72.90	90.25	80.85		74.10	41.01	95.55	651.73
Rho-NonO	PTGDR2 CRTH2	Prostaglandin D2 receptor 2 (Che	PD2R2	395		42.60	62.95	48.70	69.91		86.30	88.87	98.05	690.59
Rho-NonO	PTGER1	Prostaglandin E2 receptor EP1 su	PE2R1	402		65.75	56.68	97.40	266.64		62.50	81.21	96.35	905.06
Rho-NonO	PTGER2	Prostaglandin E2 receptor EP2 su	PE2R2	358		40.80	115.00	60.40	122.48		63.05	231.11	96.25	923.72
Rho-NonO	PTGER3	Prostaglandin E2 receptor EP3 su	PE2R3	390		38.40	34.56	23.70	32.48		77.70	45.55	95.10	469.19
Rho-NonO	PTGER4 PTGER2	Prostaglandin E2 receptor EP4 su	PE2R4	488		71.20	128.93	96.70	565.99		53.05	66.41	92.35	678.18
Rho-NonO	PTGFR	Prostaglandin F2-alpha receptor	PF2R	359		40.65	41.93	37.95	44.63		54.75	35.63	79.95	394.18
Rho-NonO	PTGIR PRIPR	Prostacyclin receptor (Prostaglar	PI2R	386		77.00	143.18	97.90	436.95		61.85	134.47	93.65	667.38
Rho-NonO	PROKR1 GPR73	Prokineticin receptor 1 (PK-R1) ((	PKR1	393		41.65	115.44	89.80	312.94		30.95	71.41	95.75	218.21
Rho-NonO	PROKR2 GPR73L	Prokineticin receptor 2 (PK-R2) ((	PKR2	384		64.20	69.33	92.35	138.97		46.55	49.85	60.95	59.04
Rho-NonO	PRLHR GPR10 G	Prolactin-releasing peptide recep	PRLHR	370		42.20	46.73	85.55	85.36		45.10	25.34	95.60	485.01
Rho-NonO	GPR65 TDAG8	Psychosine receptor (G-protein c	PSYR	337		9.20	127.49	47.85	148.03		49.15	19.80	93.95	141.85
Rho-NonO	PTAFR PAFR	Platelet-activating factor recepto	PTAFR	342		27.25	109.38	50.70	112.80		51.95	24.15	91.85	300.18
Rho-NonO	QRFPR GPR103	Pyroglutamylated RFamide pepti	QRFPR	431		28.55	115.61	48.10	118.01		55.40	25.54	94.05	228.88
Rho-NonO	RGR	RPE-retinal G protein-coupled re	RGR	291		83.30	277.40	97.75	924.27		37.45	61.75	45.10	63.87
Rho-NonO	RXFP3 RLN3R1 S	Relaxin-3 receptor 1 (RLN3 recep	RL3R1	469		47.20	99.67	68.40	111.80		84.55	60.30	95.80	548.73
Rho-NonO	RXFP4 GPR100 F	Relaxin-3 receptor 2 (RLN3 recep	RL3R2	374		29.00	90.19	46.05	98.52		71.35	81.47	94.40	140.94
Rho-NonO	RXFP1 LGR7	Relaxin receptor 1 (Leucine-rich r	RXFP1	757		25.95	93.95	36.20	70.67		50.90	55.33	76.05	267.84
Rho-NonO	RXFP2 GPR106 (	Relaxin receptor 2 (G-protein cou	RXFP2	754		57.60	103.32	70.90	73.77		61.25	67.33	87.75	149.91
**Rho-NonO**	**S1PR1 CHEDG1**	**Sphingosine 1-phosphate recept**	**S1PR1**	**382**		**81.50**	**46.72**	**90.35**	**249.48**		**94.50**	**133.40**	**62.30**	**209.51**
Rho-NonO	S1PR2 EDG5	Sphingosine 1-phosphate recepto	S1PR2	353		36.40	72.22	77.70	227.05		31.85	48.90	76.90	429.97
Rho-NonO	S1PR3 EDG3	Sphingosine 1-phosphate recepto	S1PR3	378		40.95	70.50	42.85	46.28		63.25	37.68	92.85	237.91
Rho-NonO	S1PR4 EDG6	Sphingosine 1-phosphate recepto	S1PR4	384		34.95	35.59	69.30	292.29		65.25	47.21	85.30	675.09
Rho-NonO	S1PR5 EDG8	Sphingosine 1-phosphate recepto	S1PR5	398		69.15	55.86	63.45	47.05		89.10	48.42	88.40	258.49
Rho-NonO	SSTR1	Somatostatin receptor type 1 (SS	SSR1	391		73.70	80.49	96.00	608.24		46.45	46.67	62.40	47.78
Rho-NonO	SSTR2	Somatostatin receptor type 2 (SS	SSR2	369		53.60	49.95	74.80	170.50		79.95	47.91	53.00	245.07
Rho-NonO	SSTR3	Somatostatin receptor type 3 (SS	SSR3	418		79.55	43.55	93.05	229.36		38.40	38.16	53.30	45.14
Rho-NonO	SSTR4	Somatostatin receptor type 4 (SS	SSR4	388		71.10	81.83	96.45	605.11		58.15	48.54	73.45	51.07
Rho-NonO	SSTR5	Somatostatin receptor type 5 (SS	SSR5	364		56.75	39.86	89.05	324.78		34.45	53.10	53.50	52.42
Rho-NonO	SUCNR1 GPR91	Succinate receptor 1 (G-protein c	SUCR1	334		69.90	80.32	92.85	129.48		38.90	35.94	62.50	64.90
Rho-NonO	TBXA2R	Thromboxane A2 receptor (TXA2	TA2R	343		34.90	35.63	72.20	191.75		58.75	118.23	92.40	749.28
Rho-NonO	TAAR1 TA1 TAR1	Trace amine-associated receptor	TAAR1	339		53.50	57.78	89.95	91.37		27.20	27.89	46.50	30.31
Rho-NonO	TAAR2 GPR58	Trace amine-associated receptor	TAAR2	351		61.95	64.51	84.95	80.45		44.50	29.72	60.55	35.66

Table S1. Comprehensive Protein Expression Data for 826 GPCRs

					Nt_BRIL constructs				ICL3_BRIL constructs			
Family	Gene_names	Protein_names	Target name	WT-aa-count	surface(%)	surface density	total(%)	total density	surface(%)	surface density	total(%)	total density
Rho-NonO	TAAR3 GPR57	Putative trace amine-associated	TAAR3	343	21.95	175.75	51.60	216.37	48.30	35.00	91.85	188.30
Rho-NonO	TAAR5 PNR	Trace amine-associated receptor	TAAR5	337	70.50	60.83	94.15	108.11	75.05	44.31	98.05	309.58
Rho-NonO	TAAR6 TA4 TAR4	Trace amine-associated receptor	TAAR6	345	60.80	89.25	89.65	136.25	33.75	27.92	39.50	28.19
Rho-NonO	TAAR8 GPR102 T	Trace amine-associated receptor	TAAR8	342	59.70	80.27	87.60	139.91	42.25	31.63	58.95	44.19
Rho-NonO	TAAR9 TA3 TAR3	Trace amine-associated receptor	TAAR9	348	47.15	75.77	81.10	121.29	39.80	25.23	40.85	39.79
Rho-NonO	TRHR	Thyrotropin-releasing hormone r	TRFR	398	87.70	59.48	98.20	402.50	88.85	72.71	97.35	296.30
Rho-NonO	TSHR LGR3	Thyrotropin receptor (Thyroid-st	TSHR	764	18.40	49.48	27.30	56.04	68.25	33.01	89.95	391.72
Rho-NonO	UTS2R GPR14	Urotensin-2 receptor (UR-2-R) (G	UR2R	389	71.35	44.42	93.80	73.47	83.60	135.67	90.40	156.75
Rho-NonO	AVPR1A AVPR1	Vasopressin V1a receptor (V1aR)	V1AR	418	62.35	112.68	87.35	135.29	74.05	78.58	82.75	107.34
Rho-NonO	AVPR1B AVPR3 V	Vasopressin V1b receptor (V1bR)	V1BR	424	75.90	182.08	97.65	711.95	28.35	93.54	96.00	521.59
Rho-NonO	AVPR2 ADHR DII	Vasopressin V2 receptor (V2R) (A	V2R	371	76.50	161.99	97.05	580.85	55.50	67.43	79.95	121.16
Rho-NonO	VN1R2 V1RL2	Vomeronasal type-1 receptor 2 (	VN1R2	395	42.15	62.49	82.20	382.79	64.20	86.60	94.95	560.50
Rho-NonO	VN1R3 V1RL3 FK	Vomeronasal type-1 receptor 3 (	VN1R3	311	63.85	95.42	95.60	546.82	56.05	22.02	93.25	89.19
Rho-NonO	VN1R4 V1RL4	Vomeronasal type-1 receptor 4 (	VN1R4	301	45.80	70.87	45.15	46.46	67.55	19.60	93.10	79.06
Rho-NonO	VN1R5 V1RL5	Vomeronasal type-1 receptor 5 (	VN1R5	357	34.25	79.84	36.85	60.10	55.10	20.94	81.60	51.80
Rho-NonO	VN1R17P VNRL4	Putative vomeronasal receptor-li	VNRL4	208	63.35	130.96	97.15	447.86	55.90	58.71	90.65	132.61
Rho-NonO	XCR1 CCXCR1 GP	Chemokine XC receptor 1 (G-prot	XCR1	333	34.40	25.65	58.90	45.17	57.20	44.61	95.60	354.10
Rho-NonO	VN1R1 V1RL1 VN	Vomeronasal type-1 receptor 1 (	VN1R1	353	37.10	67.15	93.15	327.02	60.30	38.18	96.70	299.60
Rho-Olf	OR10A2 OR10A2	Olfactory receptor 10A2 (HP4) (C	O10A2	303	59.95	138.47	96.40	617.80	39.90	28.65	93.25	99.06
Rho-Olf	OR10A3	Olfactory receptor 10A3 (HTPCRX	O10A3	314	77.30	128.03	97.65	937.93	5.30	30.03	11.25	47.05
Rho-Olf	OR10A4 OR10A4	Olfactory receptor 10A4 (HP2) (C	O10A4	315	17.65	187.54	49.25	97.41	16.80	20.12	45.10	23.24
Rho-Olf	OR10A5 OR10A1	Olfactory receptor 10A5 (HP3) (C	O10A5	317	41.30	86.86	58.70	84.75	7.05	42.63	12.70	19.82
Rho-Olf	OR10A6	Olfactory receptor 10A6 (Olfacto	O10A6	314	53.75	83.76	97.45	691.59	63.50	21.52	76.85	49.89
Rho-Olf	OR10A7	Olfactory receptor 10A7 (Olfacto	O10A7	316	34.25	53.64	91.55	690.76	39.20	19.02	76.85	24.10
Rho-Olf	OR10AC1P OR10	Putative olfactory receptor 10AC	O10AC	325	41.00	70.89	53.75	63.47	18.20	28.22	47.10	74.13
Rho-Olf	OR10AD1 OR10A	Olfactory receptor 10AD1 (Olfact	O10AD	317	60.10	175.57	94.35	397.82	21.90	20.15	93.00	119.88
Rho-Olf	OR10AG1	Olfactory receptor 10AG1 (Olfact	O10AG	301	40.55	81.13	53.50	64.70	49.40	28.22	93.50	64.72
Rho-Olf	OR10C1 OR10C2	Olfactory receptor 10C1 (Hs6M1-	O10C1	312	28.10	170.49	63.20	127.51	30.65	24.32	95.20	54.45
Rho-Olf	OR10D3 OR10D3	Putative olfactory receptor 10D3	O10D3	312	16.70	101.72	30.85	95.86	10.55	18.36	21.25	16.25
Rho-Olf	OR10D4P OR10D	Putative olfactory receptor 10D4	O10D4	298	64.85	137.82	97.00	611.71	39.85	18.91	88.25	54.96
Rho-Olf	OR10G2	Olfactory receptor 10G2	O10G2	310	42.70	116.46	93.90	230.36	13.95	29.80	64.15	76.97
Rho-Olf	OR10G3	Olfactory receptor 10G3 (Olfacto	O10G3	313	52.00	123.97	91.05	292.92	25.20	43.49	60.90	48.17
Rho-Olf	OR10G4	Olfactory receptor 10G4 (Olfacto	O10G4	311	18.85	160.48	54.85	102.37	24.25	30.98	66.25	33.76
Rho-Olf	OR10G6 OR10G6	Olfactory receptor 10G6 (Olfacto	O10G6	332	21.45	88.50	35.15	68.54	45.60	17.52	64.60	16.98
Rho-Olf	OR10G7	Olfactory receptor 10G7 (Olfacto	O10G7	311	17.70	135.30	55.50	101.47	15.00	17.34	17.70	20.17
Rho-Olf	OR10G8	Olfactory receptor 10G8 (Olfacto	O10G8	311	65.35	75.33	97.45	796.14	21.35	34.08	70.25	34.87
Rho-Olf	OR10G9 OR10G	Olfactory receptor 10G9 (Olfacto	O10G9	311	28.15	88.80	58.65	91.52	22.50	52.46	89.75	70.41
Rho-Olf	OR10H1	Olfactory receptor 10H1 (Olfacto	O10H1	318	48.45	159.05	71.40	113.83	23.70	25.29	52.35	35.25
Rho-Olf	OR10H2	Olfactory receptor 10H2 (Olfacto	O10H2	315	66.55	177.94	76.15	209.70	42.95	43.62	73.45	222.45

Table S1. Comprehensive Protein Expression Data for 826 GPCRs

						Nt_BRIL constructs					ICL3_BRIL constructs			
Family	Gene_names	Protein_names	Target name	WT-aa-count		surface(%)	surface density	total(%)	total density		surface(%)	surface density	total(%)	total density
Rho-Olf	OR10H3	Olfactory receptor 10H3 (Olfacto	O10H3	316		56.40	131.56	93.25	205.47		60.20	52.95	92.15	85.31
Rho-Olf	OR10H4	Olfactory receptor 10H4 (Olfacto	O10H4	316		21.30	76.09	42.05	106.71		4.95	47.84	6.05	32.56
Rho-Olf	OR10H5	Olfactory receptor 10H5 (Olfacto	O10H5	315		78.00	129.47	96.50	397.10		52.40	37.82	91.45	339.15
Rho-Olf	OR10J1	Olfactory receptor 10J1 (Olfactor	O10J1	320		63.70	55.87	97.20	656.48		13.45	46.11	85.45	158.82
Rho-Olf	OR10J3 OR10J3I	Olfactory receptor 10J3	O10J3	329		21.70	50.85	76.95	119.82		38.15	17.50	88.10	27.45
Rho-Olf	OR10J4 OR10J4I	Olfactory receptor 10J4	O10J4	311		9.80	160.25	23.20	82.91		19.70	22.38	58.50	24.86
Rho-Olf	OR10J5	Olfactory receptor 10J5 (Olfactor	O10J5	309		74.75	74.34	89.50	1,671.49		9.75	36.27	67.75	69.07
Rho-Olf	OR10J6P OR10J6	Putative olfactory receptor 10J6	O10J6	276		54.20	131.36	95.30	526.27		25.75	19.87	88.25	51.10
Rho-Olf	OR10K1	Olfactory receptor 10K1 (Olfacto	O10K1	313		47.15	77.18	94.05	394.08		25.05	42.88	92.90	146.59
Rho-Olf	OR10K2	Olfactory receptor 10K2 (Olfacto	O10K2	312		51.10	45.33	95.55	484.22		8.90	24.99	91.85	40.43
Rho-Olf	OR10P1 OR10P1	Olfactory receptor 10P1 (Olfacto	O10P1	313		31.70	122.36	46.25	77.38		19.45	31.79	52.15	40.29
Rho-Olf	OR10Q1	Olfactory receptor 10Q1 (Olfacto	O10Q1	319		47.60	336.62	57.25	206.74		9.40	20.40	63.60	38.51
Rho-Olf	OR10R2	Olfactory receptor 10R2 (Olfacto	O10R2	335		42.85	67.13	91.55	483.58		42.50	16.76	87.50	40.22
Rho-Olf	OR10S1	Olfactory receptor 10S1 (Olfacto	O10S1	331		19.40	70.33	41.90	86.71		37.85	25.45	94.35	64.50
Rho-Olf	OR10T2	Olfactory receptor 10T2 (Olfacto	O10T2	314		46.10	56.11	96.00	502.81		29.70	14.27	83.15	28.12
Rho-Olf	OR10V1	Olfactory receptor 10V1 (Olfacto	O10V1	309		69.55	82.24	85.45	1,010.96		17.25	19.45	49.25	39.97
Rho-Olf	OR10W1 OR10W	Olfactory receptor 10W1 (Olfacto	O10W1	305		72.05	156.49	97.85	802.78		21.70	22.88	57.55	29.50
Rho-Olf	OR10X1 OR10X1	Olfactory receptor 10X1 (Olfacto	O10X1	326		94.35	137.14	94.20	403.51		34.45	15.50	83.60	98.89
Rho-Olf	OR10Z1	Olfactory receptor 10Z1 (Olfacto	O10Z1	313		34.25	68.59	90.95	365.62		7.75	52.79	57.00	19.46
Rho-Olf	OR11A1 OR11A	Olfactory receptor 11A1 (Hs6M1	O11A1	315		75.20	127.67	97.65	942.10		25.95	31.87	97.55	109.05
Rho-Olf	OR11G2	Olfactory receptor 11G2 (Olfacto	O11G2	345		37.80	78.54	42.85	58.52		7.10	16.80	10.05	16.74
Rho-Olf	OR11H1	Olfactory receptor 11H1 (Olfacto	O11H1	326		74.85	126.80	96.85	906.18		41.00	13.90	54.40	19.63
Rho-Olf	OR11H2 C14orf1	Olfactory receptor 11H2 (Olfacto	O11H2	326		76.20	133.12	97.25	909.89		23.41	21.66	61.75	31.60
Rho-Olf	OR11H4	Olfactory receptor 11H4 (Olfacto	O11H4	324		34.75	85.48	47.20	64.00		37.25	23.78	94.10	76.61
Rho-Olf	OR11H6	Olfactory receptor 11H6 (Olfacto	O11H6	330		75.05	175.22	97.95	727.86		8.50	39.07	17.00	29.38
Rho-Olf	OR11H7 OR11H	Olfactory receptor 11H7 (Olfacto	O11H7	314		35.30	92.73	92.45	420.30		17.90	28.13	95.40	62.83
Rho-Olf	OR11H12	Olfactory receptor 11H12	O11HC	326		30.75	51.94	80.30	134.55		45.30	13.82	49.30	23.34
Rho-Olf	OR11L1	Olfactory receptor 11L1	O11L1	322		43.35	69.38	91.25	377.57		18.40	34.50	92.00	95.44
Rho-Olf	OR12D2	Olfactory receptor 12D2 (Hs6M1	O12D2	307		42.80	82.20	92.05	188.28		25.30	22.99	60.00	55.98
Rho-Olf	OR12D3	Olfactory receptor 12D3 (Hs6M1	O12D3	316		35.40	71.67	44.20	75.23		32.35	21.84	53.95	18.96
Rho-Olf	OR13A1	Olfactory receptor 13A1 (Olfacto	O13A1	328		46.00	76.55	93.25	735.16		18.25	24.07	63.55	62.39
Rho-Olf	OR13C2	Olfactory receptor 13C2 (Olfacto	O13C2	318		40.35	53.49	59.40	50.48		15.00	26.32	88.15	65.57
Rho-Olf	OR13C3	Olfactory receptor 13C3 (Olfacto	O13C3	347		14.60	109.57	25.60	70.07		46.85	25.09	72.65	72.10
Rho-Olf	OR13C4	Olfactory receptor 13C4 (Olfacto	O13C4	318		40.90	134.14	82.95	174.07		14.20	22.26	75.75	33.53
Rho-Olf	OR13C5	Olfactory receptor 13C5 (Olfacto	O13C5	318		31.65	146.32	94.50	617.64		38.05	28.85	96.05	73.38
Rho-Olf	OR13C8	Olfactory receptor 13C8	O13C8	320		10.10	58.37	19.25	52.43		22.40	89.60	75.70	62.21
Rho-Olf	OR13C9	Olfactory receptor 13C9 (Olfacto	O13C9	318		22.35	58.02	90.10	518.76		37.15	18.33	88.50	97.83
Rho-Olf	OR13D1	Olfactory receptor 13D1 (Olfacto	O13D1	346		24.45	44.47	87.25	672.43		23.25	15.00	48.95	27.15
Rho-Olf	OR13F1	Olfactory receptor 13F1 (Olfacto	O13F1	319		25.20	62.73	52.80	58.56		7.60	79.36	8.80	16.79
Rho-Olf	OR13G1	Olfactory receptor 13G1 (Olfacto	O13G1	307		33.85	79.62	69.60	84.03		21.75	31.08	76.70	54.84

Table S1. Comprehensive Protein Expression Data for 826 GPCRs

						Nt_BRIL constructs					ICL3_BRIL constructs			
Family	Gene_names	Protein_names	Target name	WT-aa-count		surface(%)	surface density	total(%)	total density		surface(%)	surface density	total(%)	total density
Rho-Olf	OR13H1	Olfactory receptor 13H1 (Olfacto	O13H1	308		64.05	153.34	88.45	148.07		14.70	29.69	46.80	16.76
Rho-Olf	OR13J1	Olfactory receptor 13J1 (Olfactor	O13J1	312		59.25	229.33	95.95	520.31		44.90	58.07	95.80	98.43
Rho-Olf	OR14A2 OR5AX1	Olfactory receptor 14A2 (Olfacto	O14A2	314		50.80	192.61	88.15	172.85		15.70	32.86	58.20	62.65
Rho-Olf	OR14A16 OR5AT	Olfactory receptor 14A16 (Olfact	O14AG	309		43.90	113.95	81.35	577.76		47.15	23.53	81.75	82.02
Rho-Olf	OR14C36 OR5BF	Olfactory receptor 14C36 (Olfact	O14CZ	312		57.90	52.47	97.25	815.43		52.20	33.50	92.40	230.30
Rho-Olf	OR14I1 OR5BU1	Olfactory receptor 14I1 (Olfactor	O14I1	311		55.20	82.72	96.95	522.35		42.45	41.51	82.40	134.23
Rho-Olf	OR14J1 OR5U1	Olfactory receptor 14J1 (Hs6M1-	O14J1	321		30.20	95.49	54.90	83.15		16.80	49.46	62.85	88.02
Rho-Olf	OR14K1 OR5AY1	Olfactory receptor 14K1 (Olfacto	O14K1	314		40.85	168.22	92.55	548.97		38.85	22.19	82.60	87.55
Rho-Olf	OR14L1P OR5AV	Putative olfactory receptor 14L1	O14L1	308		12.15	72.92	35.70	57.09		9.70	41.55	35.40	17.58
Rho-Olf	OR2A12 OR2A12	Olfactory receptor 2A12 (Olfacto	O2A12	310		44.25	62.88	84.35	160.32		21.75	33.16	31.85	34.34
Rho-Olf	OR2A14 OR2A14	Olfactory receptor 2A14 (OST182	O2A14	310		69.35	111.05	89.75	510.42		23.78	511.65	49.38	356.63
Rho-Olf	OR2A25 OR2A25	Olfactory receptor 2A25 (Olfacto	O2A25	310		24.20	184.84	49.75	139.64		47.20	22.65	93.25	60.17
Rho-Olf	OR2AE1 OR2AE2	Olfactory receptor 2AE1 (Olfacto	O2AE1	323		55.20	74.59	93.65	249.01		35.45	28.52	83.05	81.72
Rho-Olf	OR2AG1 OR2AG	Olfactory receptor 2AG1 (HT3) (C	O2AG1	316		21.30	120.62	41.35	108.02		19.40	22.32	93.20	37.38
Rho-Olf	OR2AG2 OR2AG	Olfactory receptor 2AG2	O2AG2	316		19.80	132.94	32.65	77.13		10.45	35.49	87.10	32.45
Rho-Olf	OR2AJ1 OR2AJ1	Olfactory receptor 2AJ1	O2AJ1	328		35.75	62.36	81.85	181.74		45.55	13.49	50.50	14.37
Rho-Olf	OR2AK2 OR2AK1	Olfactory receptor 2AK2 (Olfacto	O2AK2	335		70.10	208.45	78.55	162.78		10.90	30.65	87.20	41.69
Rho-Olf	OR2AP1 OR2AP1	Olfactory receptor 2AP1 (Olfacto	O2AP1	309		28.05	73.11	43.45	57.62		20.15	24.72	75.55	55.26
Rho-Olf	OR2AT4	Olfactory receptor 2AT4 (Olfacto	O2AT4	320		25.75	289.60	46.95	115.83		15.95	20.56	63.75	25.07
Rho-Olf	OR2T10	Olfactory receptor 2T10 (Olfacto	O2T10	312		16.85	91.33	45.35	66.09		10.60	37.27	27.30	37.34
Rho-Olf	OR2T11	Olfactory receptor 2T11 (Olfacto	O2T11	316		37.95	56.71	91.60	663.22		33.20	20.90	75.55	58.19
Rho-Olf	OR2T12	Olfactory receptor 2T12 (Olfacto	O2T12	320		70.95	92.95	90.30	1,363.52		20.65	21.83	81.75	80.88
Rho-Olf	OR2T27	Olfactory receptor 2T27 (Olfacto	O2T27	317		50.15	320.15	94.45	979.57		26.60	30.11	91.80	58.49
Rho-Olf	OR2T29	Olfactory receptor 2T29	O2T29	315		60.55	138.48	96.50	377.46		36.80	11.77	44.10	13.80
Rho-Olf	OR2T33	Olfactory receptor 2T33 (Olfacto	O2T33	320		38.95	68.72	47.58	56.27		22.55	26.03	49.10	28.09
Rho-Olf	OR2T34	Olfactory receptor 2T34 (Olfacto	O2T34	318		45.80	184.30	59.45	113.23		54.45	95.25	96.30	414.02
Rho-Olf	OR2T35	Olfactory receptor 2T35 (Olfacto	O2T35	323		34.40	75.59	54.75	55.16		55.15	25.71	86.90	171.61
Rho-Olf	OR4A15	Olfactory receptor 4A15 (Olfacto	O4A15	344		43.85	97.17	59.95	80.07		42.45	22.28	94.15	44.83
Rho-Olf	OR4A16	Olfactory receptor 4A16 (Olfacto	O4A16	328		23.55	74.77	77.55	154.63		14.80	20.90	63.05	33.66
Rho-Olf	OR4A47	Olfactory receptor 4A47 (Olfacto	O4A47	309		26.65	95.06	44.15	81.89		54.25	32.29	80.80	92.28
Rho-Olf	OR4C45	Olfactory receptor 4C45	O4C45	311		64.60	110.22	92.85	383.50		26.65	25.52	65.90	31.14
Rho-Olf	OR4C46	Olfactory receptor 4C46	O4C46	309		32.55	54.78	86.15	235.87		15.10	28.46	82.10	74.77
Rho-Olf	OR4F15	Olfactory receptor 4F15 (Olfacto	O4F15	312		38.60	156.21	47.20	89.79		22.90	30.21	63.35	58.96
Rho-Olf	OR4F17 OR4F11	Olfactory receptor 4F17 (Olfacto	O4F17	305		46.60	171.82	61.45	182.51		5.20	60.28	43.30	33.26
Rho-Olf	OR4F21 OR4F21	Olfactory receptor 4F21	O4F21	312		27.10	109.90	46.25	72.85		33.45	22.84	43.75	21.40
Rho-Olf	OR51A2	Olfactory receptor 51A2	O51A2	313		34.10	57.11	80.35	150.01		32.00	33.26	89.40	39.08
Rho-Olf	OR51A4	Olfactory receptor 51A4	O51A4	313		57.75	83.12	95.85	451.79		13.50	19.93	43.15	24.63
Rho-Olf	OR51A7	Olfactory receptor 51A7 (Olfacto	O51A7	312		66.10	117.18	90.80	179.70		11.40	18.36	37.80	28.52
Rho-Olf	OR51B2 OR51B1	Olfactory receptor 51B2 (Odoran	O51B2	312		77.75	118.85	95.70	335.42		50.80	20.25	68.00	30.86
Rho-Olf	OR51B4	Olfactory receptor 51B4 (Odoran	O51B4	310		64.60	116.56	97.90	549.11		20.40	23.15	95.10	71.07

Table S1. Comprehensive Protein Expression Data for 826 GPCRs

						Nt_BRIL constructs					ICL3_BRIL constructs			
Family	Gene_names	Protein_names	Target name	WT-aa-count		surface(%)	surface density	total(%)	total density		surface(%)	surface density	total(%)	total density
Rho-Olf	OR51B5	Olfactory receptor 51B5 (Odoran	O51B5	312		53.55	100.04	93.95	397.63		15.55	43.60	12.95	30.77
Rho-Olf	OR51B6	Olfactory receptor 51B6 (Odoran	O51B6	312		59.25	91.41	94.80	305.11		36.40	42.02	93.80	155.56
Rho-Olf	OR51D1	Olfactory receptor 51D1 (Olfacto	O51D1	324		38.95	123.39	44.20	76.98		58.15	33.89	83.00	151.93
Rho-Olf	OR51E1 GPR164	Olfactory receptor 51E1 (D-GPCR	O51E1	317		38.25	70.86	90.55	317.18		24.65	17.76	35.20	14.03
Rho-Olf	OR51E2 PSGR	Olfactory receptor 51E2 (HPRAJ)	O51E2	320		54.85	113.97	90.75	178.51		12.45	48.61	36.35	21.95
Rho-Olf	OR51F1 OR51F1	Olfactory receptor 51F1	O51F1	319		38.70	140.31	69.50	105.05		12.55	19.82	61.90	42.50
Rho-Olf	OR51F2	Olfactory receptor 51F2 (Olfacto	O51F2	342		13.15	114.34	26.60	91.89		5.55	19.69	38.60	25.55
Rho-Olf	OR51G1 OR51G	Olfactory receptor 51G1 (Olfacto	O51G1	321		79.90	35.41	97.35	257.84		40.15	28.92	37.60	27.75
Rho-Olf	OR51G2	Olfactory receptor 51G2 (Olfacto	O51G2	314		61.00	171.58	95.90	719.47		43.80	33.15	94.80	54.97
Rho-Olf	OR51H1P OR51H	Putative olfactory receptor 51H1	O51H1	302		52.60	69.24	94.85	259.91		39.50	47.00	87.25	123.05
Rho-Olf	OR51I1	Olfactory receptor 51I1 (Odorant	O51I1	314		54.75	125.90	58.55	77.24		51.40	23.49	71.55	110.87
Rho-Olf	OR51I2	Olfactory receptor 51I2 (Odorant	O51I2	312		42.70	203.69	92.00	476.96		46.55	17.50	85.35	50.88
Rho-Olf	OR51J1 OR51J1P	Olfactory receptor 51J1 (Odorant	O51J1	316		41.40	76.43	96.85	839.51		23.00	14.79	51.65	15.34
Rho-Olf	OR51L1	Olfactory receptor 51L1 (Olfacto	O51L1	315		35.70	71.41	41.45	67.11		20.30	26.76	95.75	62.46
Rho-Olf	OR51M1	Olfactory receptor 51M1 (Odora	O51M1	315		33.95	72.16	95.40	242.76		8.00	66.58	52.00	29.36
Rho-Olf	OR51Q1	Olfactory receptor 51Q1	O51Q1	317		62.10	117.09	90.00	176.66		22.60	29.65	53.50	23.52
Rho-Olf	OR51S1	Olfactory receptor 51S1 (Olfacto	O51S1	323		36.15	171.42	82.45	218.53		19.95	37.26	56.20	29.89
Rho-Olf	OR51T1	Olfactory receptor 51T1 (Olfacto	O51T1	327		31.80	100.61	51.85	69.05		36.15	28.33	66.55	27.92
Rho-Olf	OR51V1 OR51A1	Olfactory receptor 51V1 (Odoran	O51V1	321		40.30	124.96	95.00	403.31		54.30	24.47	93.45	149.80
Rho-Olf	OR52A1	Olfactory receptor 52A1 (HPFH1(	O52A1	312		35.50	66.68	91.65	364.98		15.95	37.11	56.70	48.76
Rho-Olf	OR52A4	Putative olfactory receptor 52A4	O52A4	304		18.30	114.75	93.95	412.07		29.65	14.25	79.75	28.22
Rho-Olf	OR52A5	Olfactory receptor 52A5 (Odoran	O52A5	316		26.30	78.15	36.85	64.26		30.45	37.24	91.85	34.17
Rho-Olf	OR52B2	Olfactory receptor 52B2 (Olfacto	O52B2	323		61.40	226.45	71.65	177.44		11.05	19.88	73.00	99.05
Rho-Olf	OR52B4	Olfactory receptor 52B4 (Olfacto	O52B4	314		36.00	87.10	35.90	73.99		32.80	20.86	81.70	91.95
Rho-Olf	OR52B6	Olfactory receptor 52B6 (Olfacto	O52B6	335		31.90	74.51	45.75	73.10		28.10	46.90	67.75	66.58
Rho-Olf	OR52D1	Olfactory receptor 52D1 (Odoran	O52D1	318		35.25	166.68	60.75	106.10		33.25	170.00	94.50	472.72
Rho-Olf	OR52E1 OR52E1	Olfactory receptor 52E1	O52E1	308		58.25	85.72	95.50	726.75		22.50	17.80	81.55	25.33
Rho-Olf	OR52E2	Olfactory receptor 52E2	O52E2	325		24.05	51.71	48.55	61.18		16.05	29.52	42.35	21.38
Rho-Olf	OR52E4	Olfactory receptor 52E4 (Olfacto	O52E4	312		43.75	69.89	96.55	399.48		33.40	31.43	84.05	112.00
Rho-Olf	OR52E5	Olfactory receptor 52E5	O52E5	315		21.40	145.66	31.65	144.28		13.20	34.12	52.00	40.30
Rho-Olf	OR52E6	Olfactory receptor 52E6 (Olfacto	O52E6	313		30.40	83.35	46.35	78.10		13.10	19.21	30.45	17.84
Rho-Olf	OR52E8	Olfactory receptor 52E8 (Olfacto	O52E8	317		57.60	84.22	87.70	150.24		31.95	26.17	54.60	18.52
Rho-Olf	OR52H1	Olfactory receptor 52H1 (Olfacto	O52H1	320		23.50	103.57	42.30	90.27		20.05	46.36	92.10	44.17
Rho-Olf	OR52I1	Olfactory receptor 52I1 (Olfacto	O52I1	324		43.70	80.17	95.95	509.63		16.10	22.09	63.10	33.42
Rho-Olf	OR52I2	Olfactory receptor 52I2 (Olfacto	O52I2	350		14.50	146.78	50.50	58.76		15.45	20.42	58.35	31.12
Rho-Olf	OR52J3	Olfactory receptor 52J3 (Olfacto	O52J3	311		25.85	62.22	49.75	139.64		4.65	16.52	8.60	15.17
Rho-Olf	OR52K1	Olfactory receptor 52K1 (Olfacto	O52K1	314		56.25	134.72	93.55	664.63		43.55	20.05	56.00	18.21
Rho-Olf	OR52K2	Olfactory receptor 52K2 (Olfacto	O52K2	314		46.50	81.87	95.85	498.76		8.90	17.52	5.60	19.42
Rho-Olf	OR52L1	Olfactory receptor 52L1 (Olfacto	O52L1	329		38.85	140.84	87.25	340.39		21.75	18.13	81.05	107.03
Rho-Olf	OR52L2P OR52L	Putative olfactory receptor 52L2	O52L2	319		65.00	181.67	97.50	880.05		45.80	27.33	91.95	63.27

Table S1. Comprehensive Protein Expression Data for 826 GPCRs

						Nt_BRIL constructs					ICL3_BRIL constructs			
Family	Gene_names	Protein_names	Target name	WT-aa-count		surface(%)	surface density	total(%)	total density		surface(%)	surface density	total(%)	total density
Rho-Olf	OR52M1 OR52M	Olfactory receptor 52M1 (Olfacto	O52M1	317		56.75	112.71	89.25	174.13		8.20	46.18	30.45	24.52
Rho-Olf	OR52N1	Olfactory receptor 52N1 (Olfacto	O52N1	320		8.75	50.90	61.20	156.48		45.05	18.56	88.10	58.48
Rho-Olf	OR52N2	Olfactory receptor 52N2 (Olfacto	O52N2	321		22.70	66.39	44.55	74.56		16.40	28.88	42.10	20.16
Rho-Olf	OR52N4	Olfactory receptor 52N4 (Olfacto	O52N4	321		32.90	49.42	52.85	71.69		17.75	39.85	91.20	106.22
Rho-Olf	OR52N5	Olfactory receptor 52N5 (Olfacto	O52N5	324		38.70	117.44	53.20	77.54		27.85	24.84	61.95	52.67
Rho-Olf	OR52P1P OR52P	Putative olfactory receptor 52P1	O52P1	321		54.15	47.01	97.80	533.83		20.65	25.44	76.45	26.16
Rho-Olf	OR52R1	Olfactory receptor 52R1 (Olfacto	O52R1	315		26.50	112.42	86.25	316.16		29.30	21.23	75.90	20.31
Rho-Olf	OR52W1 OR52W	Olfactory receptor 52W1 (Olfacto	O52W1	320		27.85	230.81	41.75	127.84		25.95	25.64	57.35	21.35
Rho-Olf	OR52Z1 OR52Z1	Olfactory receptor 52Z1	O52Z1	297		17.05	109.21	32.70	103.80		26.15	28.47	90.30	86.15
Rho-Olf	OR56A1	Olfactory receptor 56A1 (Olfacto	O56A1	318		54.60	64.17	46.85	69.43		48.80	21.73	93.45	38.67
Rho-Olf	OR56A3 OR56A3	Olfactory receptor 56A3 (Olfacto	O56A3	315		55.05	76.31	3.15	24.55		36.15	50.45	24.60	23.53
Rho-Olf	OR56A4	Olfactory receptor 56A4 (Olfacto	O56A4	313		80.30	155.94	97.90	801.58		52.70	14.24	56.60	14.48
Rho-Olf	OR56A5 OR56A5	Olfactory receptor 56A5	O56A5	313		60.00	74.78	92.25	252.51		36.10	14.53	75.35	20.95
Rho-Olf	OR56B1 OR56B1	Olfactory receptor 56B1 (Olfacto	O56B1	324		29.25	93.91	48.20	85.10		21.70	19.81	65.80	39.57
Rho-Olf	OR56B2P	Putative olfactory receptor 56B2	O56B2	322		15.45	39.54	20.00	35.28		21.35	20.01	31.40	16.17
Rho-Olf	OR56B4	Olfactory receptor 56B4 (Olfacto	O56B4	319		54.00	240.02	96.90	883.37		38.40	17.97	88.65	48.50
Rho-Olf	OR5AC1 OR5AC1	Olfactory receptor 5AC1 (Olfacto	O5AC1	307		71.70	96.63	91.95	133.22		49.60	72.70	70.35	43.60
Rho-Olf	OR5AC2	Olfactory receptor 5AC2 (HSA1)	O5AC2	309		37.00	68.72	45.70	66.92		58.40	32.39	97.25	147.73
Rho-Olf	OR5AK2	Olfactory receptor 5AK2	O5AK2	309		31.40	43.25	81.95	145.48		31.65	14.38	42.10	18.85
Rho-Olf	OR5AK3P OR5AI	Putative olfactory receptor 5AK3	O5AK3	298		21.80	54.55	83.15	354.14		8.35	15.68	74.35	32.59
Rho-Olf	OR5AL1 OR5AL1	Olfactory receptor 5AL1 (Olfacto	O5AL1	328		21.90	66.16	34.00	55.38		28.33	16.09	58.70	30.00
Rho-Olf	OR5AN1	Olfactory receptor 5AN1 (Olfacto	O5AN1	311		59.60	101.99	96.60	880.81		27.70	28.63	70.20	23.57
Rho-Olf	OR5AP2	Olfactory receptor 5AP2	O5AP2	316		53.80	57.98	93.90	454.51		10.80	32.55	36.65	18.27
Rho-Olf	OR5AR1	Olfactory receptor 5AR1 (Olfacto	O5AR1	310		11.20	83.21	24.45	79.93		10.95	44.53	88.65	34.72
Rho-Olf	OR5AS1	Olfactory receptor 5AS1 (Olfacto	O5AS1	324		58.50	125.09	92.75	325.43		14.10	34.00	49.80	32.51
Rho-Olf	OR5AU1	Olfactory receptor 5AU1 (Olfacto	O5AU1	362		24.05	158.08	39.95	58.76		17.80	58.21	95.25	108.11
Rho-Olf	OR5H14	Olfactory receptor 5H14	O5H14	310		59.00	112.55	93.80	607.97		30.80	31.28	81.50	189.79
Rho-Olf	OR5H15	Olfactory receptor 5H15	O5H15	313		64.45	165.93	93.10	217.81		26.55	27.06	57.85	22.21
Rho-Olf	OR6C65	Olfactory receptor 6C65	O6C65	312		39.50	63.51	87.85	180.35		22.50	26.84	92.60	40.44
Rho-Olf	OR6C68	Olfactory receptor 6C68	O6C68	312		38.55	89.25	93.60	296.43		27.75	28.88	94.30	77.48
Rho-Olf	OR6C70	Olfactory receptor 6C70	O6C70	312		59.55	143.06	87.75	1,573.84		10.46	220.63	24.54	150.52
Rho-Olf	OR6C74	Olfactory receptor 6C74	O6C74	312		43.20	143.00	67.90	125.17		21.15	34.71	90.40	40.04
Rho-Olf	OR6C75	Olfactory receptor 6C75	O6C75	312		38.90	109.32	49.65	87.07		21.20	23.29	33.20	30.77
Rho-Olf	OR6C76	Olfactory receptor 6C76	O6C76	312		37.45	49.61	88.60	275.09		14.30	16.09	77.65	54.55
Rho-Olf	OR7E24 OR7E24	Olfactory receptor 7E24 (Olfacto	O7E24	339		12.15	94.14	87.10	852.24		32.40	17.33	64.75	52.99
Rho-Olf	OR1A1	Olfactory receptor 1A1 (Olfactor	OR1A1	309		35.40	141.95	86.95	1,358.96		54.80	20.78	73.85	78.76
Rho-Olf	OR1A2	Olfactory receptor 1A2 (Olfactor	OR1A2	309		29.10	53.39	73.70	521.63		9.85	20.39	55.95	35.40
Rho-Olf	OR1B1	Olfactory receptor 1B1 (Olfactor	OR1B1	318		23.75	135.80	79.05	197.78		16.20	42.29	20.75	37.82
Rho-Olf	OR1C1	Olfactory receptor 1C1 (Olfactor	OR1C1	314		37.40	61.75	86.15	151.11		47.05	14.61	58.30	18.66
Rho-Olf	OR1D2 OLFR1	Olfactory receptor 1D2 (Olfactor	OR1D2	312		30.60	137.45	49.75	70.44		44.75	40.25	97.50	166.71

Table S1. Comprehensive Protein Expression Data for 826 GPCRs

					Nt_BRIL constructs				ICL3_BRIL constructs			
Family	Gene_names	Protein_names	Target name	WT-aa-count	surface(%)	surface density	total(%)	total density	surface(%)	surface density	total(%)	total density
Rho-Olf	OR1D4	Olfactory receptor 1D4 (Olfactor	OR1D4	311	32.60	150.96	92.60	558.87	39.90	28.65	93.25	99.06
Rho-Olf	OR1D5	Olfactory receptor 1D5 (Olfactor	OR1D5	312	74.30	199.47	97.00	523.82	25.60	76.46	79.50	246.09
Rho-Olf	OR1E1 OR1E5 O	Olfactory receptor 1E1 (Olfactory	OR1E1	314	41.70	113.09	56.95	103.20	16.30	70.94	27.00	29.24
Rho-Olf	OR1E2 OR1E4	Olfactory receptor 1E2 (Olfactory	OR1E2	323	63.30	371.73	96.35	696.62	36.50	18.20	80.35	50.16
Rho-Olf	OR1E3 OR1E3P	Olfactory receptor 1E3 (Olfactory	OR1E3	343	33.80	93.80	45.80	75.53	50.90	20.16	92.35	63.48
Rho-Olf	OR1F1 OLFMF O	Olfactory receptor 1F1 (Olfactory	OR1F1	312	14.60	103.20	29.45	71.06	19.80	26.87	54.65	29.77
Rho-Olf	OR1F2P OR1F2	Putative olfactory receptor 1F2 (	OR1F2	312	42.05	353.19	56.95	213.24	12.10	19.26	66.30	66.70
Rho-Olf	OR1F12 OR1F12	Olfactory receptor 1F12 (Hs6M1-	OR1FC	337	14.35	264.80	31.65	99.33	12.70	20.42	40.80	34.35
Rho-Olf	OR1G1 OR1G2	Olfactory receptor 1G1 (Olfactor	OR1G1	313	18.25	87.01	37.65	96.27	16.45	25.43	91.10	97.49
Rho-Olf	OR1I1	Olfactory receptor 1I1 (Olfactory	OR1I1	355	16.95	75.18	32.45	74.42	22.20	67.33	90.55	88.41
Rho-Olf	OR1J1	Olfactory receptor 1J1 (Olfactory	OR1J1	322	73.80	177.42	97.75	973.39	11.00	47.58	15.95	43.77
Rho-Olf	OR1J2 OR1J3 OR	Olfactory receptor 1J2 (HSA5) (H	OR1J2	313	22.05	208.95	85.65	245.96	24.85	22.29	58.95	30.03
Rho-Olf	OR1J4	Olfactory receptor 1J4 (HTPCRX0	OR1J4	313	49.65	256.03	87.70	1,812.69	9.50	336.79	34.32	224.29
Rho-Olf	OR1K1	Olfactory receptor 1K1	OR1K1	316	17.50	174.52	33.15	106.53	16.85	15.13	79.70	25.25
Rho-Olf	OR1L1 OR1L2	Olfactory receptor 1L1 (Olfactory	OR1L1	360	56.40	213.27	72.90	190.66	11.05	35.02	92.60	40.40
Rho-Olf	OR1L3	Olfactory receptor 1L3 (Olfactory	OR1L3	324	30.75	98.17	42.45	103.78	50.85	25.88	95.65	60.23
Rho-Olf	OR1L4 OR1L5	Olfactory receptor 1L4 (OST046)	OR1L4	311	38.60	86.84	49.20	86.07	10.30	33.65	66.90	21.45
Rho-Olf	OR1L6 OR1L7	Olfactory receptor 1L6 (Olfactory	OR1L6	347	65.45	184.97	93.75	362.82	23.60	28.02	64.80	35.94
Rho-Olf	OR1L8	Olfactory receptor 1L8 (Olfactory	OR1L8	309	78.65	148.55	96.50	635.06	31.85	24.45	91.60	44.58
Rho-Olf	OR1M1	Olfactory receptor 1M1 (Olfactor	OR1M1	313	27.40	102.26	39.20	72.74	32.60	29.88	69.65	36.49
Rho-Olf	OR1N1 OR1N3	Olfactory receptor 1N1 (Olfactor	OR1N1	311	27.50	95.87	88.75	250.00	41.95	18.12	88.10	84.93
Rho-Olf	OR1N2	Olfactory receptor 1N2 (Olfactor	OR1N2	330	36.85	101.86	50.65	81.65	19.60	36.58	94.40	77.03
Rho-Olf	OR1P1 OR1P1P	Olfactory receptor 1P1 (Olfactory	OR1P1	330	35.70	142.92	57.40	96.91	53.20	15.26	42.35	20.36
Rho-Olf	OR1Q1 OR1Q2 (	Olfactory receptor 1Q1 (OST226)	OR1Q1	314	49.65	120.33	96.30	581.49	12.60	31.27	68.35	21.17
Rho-Olf	OR1S1	Olfactory receptor 1S1 (Olfactory	OR1S1	325	42.45	87.00	82.80	109.22	39.95	23.06	71.30	41.68
Rho-Olf	OR1S2	Olfactory receptor 1S2 (Olfactory	OR1S2	325	48.35	114.51	66.00	120.80	31.20	34.38	73.80	33.50
Rho-Olf	OR2A1; OR2A42	Olfactory receptor 2A1/2A42 (Ol	OR2A1	310	32.60	101.90	91.80	306.26	9.10	20.01	30.95	17.50
Rho-Olf	OR2A2 OR2A17	Olfactory receptor 2A2 (Olfactor	OR2A2	318	69.75	90.56	97.35	451.08	43.45	24.36	95.30	72.54
Rho-Olf	OR2A4 OR2A10	Olfactory receptor 2A4 (Olfactor	OR2A4	310	52.00	47.90	94.30	218.38	54.85	21.62	86.75	50.45
Rho-Olf	OR2A5 OR2A26	Olfactory receptor 2A5 (Olfactor	OR2A5	311	79.65	143.74	98.45	538.18	58.80	39.43	95.05	245.27
Rho-Olf	OR2A7	Olfactory receptor 2A7 (Olfactor	OR2A7	310	69.70	152.93	97.40	603.36	49.30	21.46	78.60	51.00
Rho-Olf	OR2B2 OR2B9	Olfactory receptor 2B2 (Hs6M1-1	OR2B2	357	22.75	70.41	43.20	57.28	18.75	26.81	72.50	35.65
Rho-Olf	OR2B3 OR2B3P	Putative olfactory receptor 2B3 (	OR2B3	313	57.00	148.02	73.00	101.99	20.95	24.03	48.35	26.44
Rho-Olf	OR2B6 OR2B1 O	Olfactory receptor 2B6 (Hs6M1-3	OR2B6	313	14.95	55.39	32.25	57.39	12.55	19.55	23.20	16.64
Rho-Olf	OR2B8P OR2B8	Putative olfactory receptor 2B8 (	OR2B8	312	32.80	62.97	71.40	542.45	9.95	31.86	38.60	37.92
Rho-Olf	OR2B11	Olfactory receptor 2B11	OR2BB	317	51.00	70.31	61.45	66.87	50.50	19.83	84.55	29.09
Rho-Olf	OR2C1 OR2C2P	Olfactory receptor 2C1 (OLFmf3)	OR2C1	312	53.35	81.20	92.15	330.57	43.00	18.45	78.25	58.21
Rho-Olf	OR2C3 OR2C4 O	Olfactory receptor 2C3 (Olfactory	OR2C3	320	34.40	62.33	96.00	471.23	23.15	33.71	87.15	26.82
Rho-Olf	OR2D2 OR2D1	Olfactory receptor 2D2 (HB2) (Ol	OR2D2	308	34.85	102.93	61.35	86.44	20.55	25.78	48.45	17.29
Rho-Olf	OR2D3	Olfactory receptor 2D3 (Olfactor	OR2D3	330	8.70	40.67	43.20	171.93	26.45	35.66	68.00	80.88

Table S1. Comprehensive Protein Expression Data for 826 GPCRs

						Nt_BRIL constructs					ICL3_BRIL constructs			
Family	Gene_names	Protein_names	Target name	WT-aa-count		surface(%)	surface density	total(%)	total density		surface(%)	surface density	total(%)	total density
Rho-Olf	OR2F1 OLF3 OR2	Olfactory receptor 2F1 (Olfactory	OR2F1	317		69.50	253.80	79.10	182.53		33.40	37.56	75.45	103.69
Rho-Olf	OR2F2	Olfactory receptor 2F2 (Olfactory	OR2F2	317		45.65	86.11	60.50	71.77		41.80	20.31	78.40	43.96
Rho-Olf	OR2G2	Olfactory receptor 2G2 (Olfactory	OR2G2	317		71.65	60.70	92.75	223.37		42.00	17.85	70.15	41.42
Rho-Olf	OR2G3	Olfactory receptor 2G3 (Olfactory	OR2G3	309		18.70	69.76	46.25	56.59		25.70	30.13	90.20	41.31
Rho-Olf	OR2G6	Olfactory receptor 2G6	OR2G6	316		15.80	87.16	31.45	61.00		16.75	32.95	88.20	40.86
Rho-Olf	OR2H1 OR2H6 C	Olfactory receptor 2H1 (Hs6M1-1	OR2H1	316		33.35	70.76	92.40	547.08		40.25	17.66	86.35	63.35
Rho-Olf	OR2H2 FAT11 O	Olfactory receptor 2H2 (Hs6M1-1	OR2H2	312		57.15	134.03	95.45	650.82		12.10	40.59	62.85	31.84
Rho-Olf	OR2I1P OR2I2 O	Putative olfactory receptor 2I1 (P	OR2I1	316		73.40	126.15	97.60	566.18		25.20	16.59	70.20	33.22
Rho-Olf	OR2J1 OR2J1P	Olfactory receptor 2J1 (Hs6M1-4	OR2J1	312		40.70	94.60	95.60	502.87		37.95	27.64	87.65	29.29
Rho-Olf	OR2J2	Olfactory receptor 2J2 (Hs6M1-6	OR2J2	312		25.85	109.02	36.45	66.69		43.85	12.72	23.65	17.77
Rho-Olf	OR2J3	Olfactory receptor 2J3 (Hs6M1-3	OR2J3	311		33.60	158.91	78.80	189.92		24.75	39.88	60.20	33.21
Rho-Olf	OR2K2 OR2AR1P	Olfactory receptor 2K2 (HTPCRH0	OR2K2	345		31.05	57.95	57.95	68.81		6.75	31.86	26.60	21.40
Rho-Olf	OR2L2 OR2L12 (	Olfactory receptor 2L2 (HTPCRH0	OR2L2	312		27.70	63.61	39.25	60.65		32.50	17.56	63.95	35.93
Rho-Olf	OR2L3	Olfactory receptor 2L3	OR2L3	312		35.85	56.42	94.40	571.96		18.80	18.09	84.55	28.60
Rho-Olf	OR2L5 OR2L11	Olfactory receptor 2L5 (Olfactory	OR2L5	312		30.85	71.41	88.15	307.51		16.60	28.45	80.50	38.20
Rho-Olf	OR2L8	Olfactory receptor 2L8 (Olfactory	OR2L8	312		30.30	107.25	44.00	69.27		17.25	31.68	77.95	46.15
Rho-Olf	OR2L13 OR2L14	Olfactory receptor 2L13 (Olfactor	OR2LD	312		36.35	121.83	45.85	109.06		16.50	19.13	70.50	72.55
Rho-Olf	OR2M2	Olfactory receptor 2M2 (OST423	OR2M2	347		23.55	51.85	39.10	59.73		43.75	15.59	39.90	15.11
Rho-Olf	OR2M3 OR2M3P	Olfactory receptor 2M3 (Olfactor	OR2M3	312		78.65	155.49	98.20	957.74		14.40	25.10	27.85	22.02
Rho-Olf	OR2M4	Olfactory receptor 2M4 (HTPCRX	OR2M4	311		36.70	76.25	51.90	55.18		52.70	19.63	94.45	34.04
Rho-Olf	OR2M5 OR2M5P	Olfactory receptor 2M5	OR2M5	312		67.15	160.33	96.30	498.56		17.95	19.63	52.60	17.81
Rho-Olf	OR2M7	Olfactory receptor 2M7 (Olfactor	OR2M7	312		25.35	150.98	82.75	257.71		18.40	33.50	96.30	55.58
Rho-Olf	OR2S2	Olfactory receptor 2S2 (Olfactory	OR2S1	319		48.10	94.29	97.45	550.73		46.05	32.24	96.30	155.78
Rho-Olf	OR2T1	Olfactory receptor 2T1 (Olfactory	OR2T1	369		33.10	75.58	46.80	70.31		22.00	36.61	72.80	146.09
Rho-Olf	OR2T2 OR2T2P	Olfactory receptor 2T2 (Olfactory	OR2T2	324		53.40	174.98	94.45	464.52		19.55	34.74	93.30	105.59
Rho-Olf	OR2T3	Olfactory receptor 2T3	OR2T3	318		38.45	236.51	59.75	131.93		48.75	98.84	98.00	543.57
Rho-Olf	OR2T4	Olfactory receptor 2T4 (Olfactory	OR2T4	348		12.95	133.75	30.55	90.52		10.80	29.64	26.35	22.74
Rho-Olf	OR2T5	Olfactory receptor 2T5 (Olfactory	OR2T5	315		71.95	120.02	98.10	549.76		25.30	29.91	84.30	38.76
Rho-Olf	OR2T6 OR2T6P (	Olfactory receptor 2T6 (OST703)	OR2T6	308		35.00	140.80	56.75	106.18		38.25	21.13	67.70	28.33
Rho-Olf	OR2T7 OR2T7P	Olfactory receptor 2T7 (OST723)	OR2T7	308		31.85	81.36	56.40	78.02		35.70	22.55	92.65	56.59
Rho-Olf	OR2T8 OR2T8P	Olfactory receptor 2T8	OR2T8	312		32.10	72.64	53.45	67.74		38.45	29.77	89.50	26.27
Rho-Olf	OR2V1	Olfactory receptor 2V1	OR2V1	315		69.60	107.62	97.55	614.16		32.05	26.20	92.85	33.78
Rho-Olf	OR2V2 OR2V3	Olfactory receptor 2V2 (Olfactory	OR2V2	315		27.05	91.88	40.15	74.08		14.60	20.15	39.85	21.59
Rho-Olf	OR2W1	Olfactory receptor 2W1 (Hs6M1-	OR2W1	320		42.35	93.86	93.70	401.43		41.95	22.09	93.00	64.66
Rho-Olf	OR2W3 OR2W3	Olfactory receptor 2W3 (Olfactor	OR2W3	314		31.55	76.23	67.65	73.37		20.35	30.91	74.20	38.45
Rho-Olf	OR2W5 OR2W5	Putative olfactory receptor 2W5	OR2W5	320		25.40	220.38	91.55	512.87		16.00	28.04	88.40	115.09
Rho-Olf	OR2W6P OR2W	Putative olfactory receptor 2W6	OR2W6	318		18.15	106.34	69.75	677.44		33.10	16.92	96.15	48.73
Rho-Olf	OR2Y1	Olfactory receptor 2Y1 (Olfactory	OR2Y1	311		26.50	78.09	39.70	69.97		37.05	23.38	89.30	82.88
Rho-Olf	OR2Z1 OR2Z2	Olfactory receptor 2Z1 (Olfactory	OR2Z1	314		57.80	74.67	95.25	1,002.21		27.15	24.12	86.25	45.54
Rho-Olf	OR3A1 OLFRA03	Olfactory receptor 3A1 (Olfactory	OR3A1	315		42.85	72.90	93.40	783.51		15.90	23.77	62.05	25.25

Table S1. Comprehensive Protein Expression Data for 826 GPCRs

						Nt_BRIL constructs					ICL3_BRIL constructs			
**Family**	**Gene_names**	**Protein_names**	**Target name**	**WT-aa-count**		**surface(%)**	**surface density**	**total(%)**	**total density**		**surface(%)**	**surface density**	**total(%)**	**total density**
Rho-Olf	OR3A2 OLFRA04	Olfactory receptor 3A2 (Olfactor	OR3A2	321		22.45	130.02	92.00	818.21		17.15	27.92	42.60	18.93
Rho-Olf	OR3A3 OR3A6 C	Olfactory receptor 3A3 (Olfactor	OR3A3	321		53.00	67.55	95.05	1,092.87		20.75	23.63	68.25	26.75
Rho-Olf	OR3A4P OR3A4	Putative olfactory receptor 3A4 (	OR3A4	348		17.35	229.15	88.80	649.98		33.20	17.84	88.90	70.44
Rho-Olf	OR4A4P	Putative olfactory receptor 4A4 (	OR4A4	299		43.50	83.58	4.30	60.88		10.55	19.44	50.10	28.39
Rho-Olf	OR4A5	Olfactory receptor 4A5 (Olfactor	OR4A5	315		21.25	65.90	48.30	61.14		11.35	19.25	16.35	16.36
Rho-Olf	OR4A8P OR4A8	Putative olfactory receptor 4A8 (	OR4A8	315		29.35	144.74	91.75	399.45		34.05	25.93	87.20	37.00
Rho-Olf	OR4B1	Olfactory receptor 4B1 (OST208)	OR4B1	309		30.35	62.87	56.00	64.98		34.00	22.34	93.80	63.49
Rho-Olf	OR4C3	Olfactory receptor 4C3 (Olfactor	OR4C3	302		51.00	151.08	92.75	283.71		38.15	23.68	73.95	155.35
Rho-Olf	OR4C5	Olfactory receptor 4C5 (Olfactor	OR4C5	326		32.65	121.12	80.15	137.97		39.00	35.99	71.15	59.50
Rho-Olf	OR4C6	Olfactory receptor 4C6 (Olfactor	OR4C6	309		34.75	74.68	87.40	447.69		40.55	17.32	80.65	32.08
Rho-Olf	OR4C11 OR4C11	Olfactory receptor 4C11 (Olfacto	OR4CB	310		59.95	69.14	96.50	515.12		20.65	31.32	86.40	45.47
Rho-Olf	OR4C12	Olfactory receptor 4C12 (Olfacto	OR4CC	309		63.20	206.11	69.85	122.97		16.45	18.09	84.85	40.91
Rho-Olf	OR4C13	Olfactory receptor 4C13 (Olfacto	OR4CD	309		62.65	162.24	96.40	782.55		18.50	32.21	79.75	36.99
Rho-Olf	OR4C15	Olfactory receptor 4C15 (Olfacto	OR4CF	316		67.55	144.50	95.25	388.41		13.40	39.05	60.40	43.78
Rho-Olf	OR4C16	Olfactory receptor 4C16 (Olfacto	OR4CG	310		35.30	104.18	50.60	86.10		46.10	31.83	74.75	74.56
Rho-Olf	OR4D1 OR4D3	Olfactory receptor 4D1 (Olfactor	OR4D1	310		72.45	113.10	93.85	973.12		13.80	37.09	61.50	58.26
Rho-Olf	OR4D2	Olfactory receptor 4D2 (B-lymph	OR4D2	307		61.30	302.29	72.35	231.51		17.70	24.03	95.80	71.94
Rho-Olf	OR4D5	Olfactory receptor 4D5 (Olfactor	OR4D5	318		28.95	108.44	35.20	111.16		15.15	33.24	95.75	80.70
Rho-Olf	OR4D6	Olfactory receptor 4D6 (Olfactor	OR4D6	314		9.45	122.85	24.65	93.05		57.05	48.79	78.15	307.06
Rho-Olf	OR4D9	Olfactory receptor 4D9 (Olfactor	OR4D9	314		27.70	129.47	57.35	86.16		17.25	30.74	54.25	20.52
Rho-Olf	OR4D10 OR4D1	Olfactory receptor 4D10 (Olfacto	OR4DA	311		29.10	55.92	52.50	48.59		42.25	26.17	90.05	51.51
Rho-Olf	OR4D11 OR4D1	Olfactory receptor 4D11	OR4DB	311		26.25	93.52	61.35	93.84		47.40	13.02	28.00	35.99
Rho-Olf	OR4E1 OR4E1P	Olfactory receptor 4E1 (Olfactor	OR4E1	316		24.90	100.76	92.80	389.63		36.55	19.03	82.50	68.31
Rho-Olf	OR4E2	Olfactory receptor 4E2 (Olfactor	OR4E2	313		28.50	57.79	51.10	56.02		13.25	24.79	5.40	67.96
Rho-Olf	OR4F3; OR4F16;	Olfactory receptor 4F3/4F16/4F2	OR4F3	312		19.65	50.01	39.00	59.80		39.65	37.33	50.05	31.45
Rho-Olf	OR4F4	Olfactory receptor 4F4 (HS14a-1-	OR4F4	305		10.65	80.15	27.60	70.65		8.75	75.71	45.05	31.34
Rho-Olf	OR4F5	Olfactory receptor 4F5	OR4F5	305		23.45	59.54	46.75	70.30		19.00	30.37	12.25	35.43
Rho-Olf	OR4F6 OR4F12	Olfactory receptor 4F6 (Olfactor	OR4F6	312		73.90	99.63	97.50	413.30		16.70	43.65	10.60	21.57
Rho-Olf		Putative olfactory receptor GPCR	OR4FL	273		44.30	116.95	94.50	634.97		32.50	35.35	70.55	40.69
Rho-Olf	OR4K1	Olfactory receptor 4K1 (Olfactor	OR4K1	311		21.30	45.22	33.70	45.81		14.85	28.43	96.40	58.85
Rho-Olf	OR4K2	Olfactory receptor 4K2 (Olfactor	OR4K2	314		24.50	179.18	55.10	105.62		9.45	16.87	13.40	17.02
Rho-Olf	OR4K3 OR4K3P	Olfactory receptor 4K3 (Olfactor	OR4K3	315		25.60	79.48	31.25	73.26		16.40	33.19	63.35	72.29
Rho-Olf	OR4K5	Olfactory receptor 4K5 (Olfactor	OR4K5	323		46.15	107.44	58.20	75.70		56.85	25.16	96.70	67.96
Rho-Olf	OR4K13	Olfactory receptor 4K13 (Olfacto	OR4KD	304		30.40	71.58	46.05	67.37		23.15	23.73	91.00	47.59
Rho-Olf	OR4K14	Olfactory receptor 4K14 (Olfacto	OR4KE	310		44.75	90.11	95.65	426.24		25.10	26.02	91.00	63.03
Rho-Olf	OR4K15	Olfactory receptor 4K15 (Olfacto	OR4KF	348		27.05	137.92	78.15	167.93		6.45	55.36	30.70	21.79
Rho-Olf	OR4K17	Olfactory receptor 4K17 (Olfacto	OR4KH	315		71.90	136.78	95.95	430.18		46.75	14.30	52.70	19.86
Rho-Olf	OR4L1 OR4L2P	Olfactory receptor 4L1 (Olfactor	OR4L1	312		62.90	194.60	97.65	785.73		43.00	15.77	64.05	17.94
Rho-Olf	OR4M1	Olfactory receptor 4M1 (Olfactor	OR4M1	313		13.15	65.51	28.15	53.63		17.30	48.07	47.40	32.61
Rho-Olf	OR4M2	Olfactory receptor 4M2 (Olfactor	OR4M2	313		49.85	110.24	95.95	671.89		21.45	22.39	91.70	35.27

Table S1. Comprehensive Protein Expression Data for 826 GPCRs

						Nt_BRIL constructs					ICL3_BRIL constructs			
Family	Gene_names	Protein_names	Target name	WT-aa-count		surface(%)	surface density	total(%)	total density		surface(%)	surface density	total(%)	total density
Rho-Olf	OR4N2	Olfactory receptor 4N2 (Olfactor	OR4N2	307		49.35	63.48	92.40	584.73		49.35	63.48	92.40	584.73
Rho-Olf	OR4N4	Olfactory receptor 4N4 (Olfactor	OR4N4	316		17.50	97.15	33.90	59.30		50.05	19.62	96.20	43.28
Rho-Olf	OR4N5	Olfactory receptor 4N5 (Olfactor	OR4N5	308		39.00	48.55	91.35	131.95		22.65	15.56	62.30	25.45
Rho-Olf	OR4P4 OR4P3P	Olfactory receptor 4P4 (Olfactory	OR4P4	312		59.95	99.52	94.20	327.80		16.55	14.88	4.50	16.10
Rho-Olf	OR4Q2 OR4Q2P	Olfactory receptor 4Q2 (olfactory	OR4Q2	313		13.75	67.13	30.15	63.33		14.50	36.12	93.70	84.58
Rho-Olf	OR4Q3 C14orf1	Olfactory receptor 4Q3 (Olfactor	OR4Q3	313		56.10	119.96	96.35	518.72		27.70	29.73	61.00	29.95
Rho-Olf	OR4S1	Olfactory receptor 4S1 (Olfactory	OR4S1	309		22.35	105.24	38.40	75.82		30.60	34.82	57.95	30.03
Rho-Olf	OR4S2 OR4S2P	Olfactory receptor 4S2 (Olfactory	OR4S2	311		62.85	239.17	78.75	197.77		15.95	24.44	67.50	74.36
Rho-Olf	OR4X1	Olfactory receptor 4X1 (Olfactory	OR4X1	305		45.45	71.90	96.15	387.55		8.15	40.52	56.25	62.31
Rho-Olf	OR4X2	Olfactory receptor 4X2 (Olfactory	OR4X2	303		59.05	157.63	65.90	144.33		11.45	17.75	55.70	39.17
Rho-Olf	OR5A1 OR5A1P	Olfactory receptor 5A1 (OST181)	OR5A1	315		66.60	124.51	97.25	768.19		40.30	83.51	86.80	127.18
Rho-Olf	OR5A2	Olfactory receptor 5A2 (Olfactory	OR5A2	324		67.40	79.71	96.20	949.26		27.20	21.04	72.70	69.69
Rho-Olf	OR5B2	Olfactory receptor 5B2 (OST073)	OR5B2	309		37.20	124.72	91.85	346.94		28.60	31.76	36.40	19.60
Rho-Olf	OR5B3 OR5B13	Olfactory receptor 5B3 (Olfactory	OR5B3	314		39.90	66.55	96.75	479.46		4.05	38.85	83.60	26.78
Rho-Olf	OR5B12 OR5B12	Olfactory receptor 5B12 (Olfacto	OR5BC	314		34.50	62.74	92.45	754.39		33.45	16.90	77.10	42.79
Rho-Olf	OR5B17 OR5B2	Olfactory receptor 5B17 (Olfacto	OR5BH	314		44.05	99.50	56.70	90.62		18.10	18.73	58.15	43.41
Rho-Olf	OR5B21	Olfactory receptor 5B21	OR5BL	309		17.85	167.59	91.45	581.28		7.45	21.33	19.25	27.15
Rho-Olf	OR5C1 OR5C2P	Olfactory receptor 5C1 (Olfactory	OR5C1	320		35.65	159.40	95.75	895.73		33.00	25.45	79.55	44.79
Rho-Olf	OR5D13	Olfactory receptor 5D13 (Olfacto	OR5DD	314		30.35	113.84	89.10	481.51		19.75	21.73	28.90	17.87
Rho-Olf	OR5D14	Olfactory receptor 5D14 (Olfacto	OR5DE	314		65.80	136.77	97.95	576.19		40.15	24.55	91.85	60.67
Rho-Olf	OR5D16	Olfactory receptor 5D16 (Olfacto	OR5DG	328		16.05	100.50	88.85	370.21		57.05	33.40	93.05	391.78
Rho-Olf	OR5D18	Olfactory receptor 5D18 (Olfacto	OR5DI	313		45.70	56.48	94.95	705.39		28.45	20.11	59.90	18.16
Rho-Olf	OR5F1	Olfactory receptor 5F1 (Olfactory	OR5F1	314		34.70	188.99	50.75	112.36		50.55	17.58	85.50	33.88
Rho-Olf	OR5G3 OR5G3P	Olfactory receptor 5G3 (Olfactor	OR5G3	314		50.90	102.60	61.45	72.30		53.85	16.14	90.05	38.55
Rho-Olf	OR5H1	Olfactory receptor 5H1 (HTPCRX1	OR5H1	313		18.55	82.29	40.05	64.57		36.15	29.76	44.65	30.11
Rho-Olf	OR5H2	Olfactory receptor 5H2 (Olfactor	OR5H2	314		26.20	93.16	40.65	79.66		34.10	34.63	95.80	133.14
Rho-Olf	OR5H6	Olfactory receptor 5H6 (Olfactor	OR5H6	325		33.60	105.98	93.40	306.68		31.55	31.83	86.90	89.85
Rho-Olf	OR5I1 OLF1	Olfactory receptor 5I1 (Olfactory	OR5I1	314		30.40	103.13	55.60	88.22		10.00	27.83	81.20	37.77
Rho-Olf	OR5J2	Olfactory receptor 5J2 (Olfactory	OR5J2	312		33.45	57.99	74.05	147.95		22.75	49.42	79.15	38.17
Rho-Olf	OR5K1	Olfactory receptor 5K1 (HTPCRX1	OR5K1	308		55.65	206.58	59.80	139.85		19.80	43.50	71.10	184.24
Rho-Olf	OR5K2	Olfactory receptor 5K2 (Olfactory	OR5K2	316		60.10	64.05	96.90	537.33		25.85	22.28	74.65	48.75
Rho-Olf	OR5K3	Olfactory receptor 5K3	OR5K3	321		14.15	68.36	71.80	625.84		9.55	48.93	49.40	42.26
Rho-Olf	OR5K4	Olfactory receptor 5K4	OR5K4	321		23.65	149.31	36.10	99.53		29.25	19.31	50.05	17.81
Rho-Olf	OR5L1	Olfactory receptor 5L1 (OST262)	OR5L1	311		42.20	288.37	97.10	896.42		43.55	20.27	80.30	40.89
Rho-Olf	OR5L2	Olfactory receptor 5L2 (HTPCRX1	OR5L2	311		44.90	152.30	94.90	676.72		45.90	39.30	90.05	206.82
Rho-Olf	OR5M1	Olfactory receptor 5M1 (OST050	OR5M1	315		32.80	79.29	89.60	421.25		11.60	20.92	48.30	19.73
Rho-Olf	OR5M3	Olfactory receptor 5M3 (Olfactor	OR5M3	307		63.15	72.59	97.10	677.85		28.70	27.10	82.05	42.73
Rho-Olf	OR5M8	Olfactory receptor 5M8 (Olfactor	OR5M8	311		59.15	84.90	97.55	547.01		34.90	26.17	83.75	47.49
Rho-Olf	OR5M9	Olfactory receptor 5M9 (Olfactor	OR5M9	310		33.35	46.44	90.75	326.77		23.30	17.10	78.75	53.79
Rho-Olf	OR5M10	Olfactory receptor 5M10 (Olfacto	OR5MA	315		24.10	162.17	41.15	93.45		11.15	37.93	61.25	55.03

Table S1. Comprehensive Protein Expression Data for 826 GPCRs

						Nt_BRIL constructs					ICL3_BRIL constructs			
Family	Gene_names	Protein_names	Target name	WT-aa-count		surface(%)	surface density	total(%)	total density		surface(%)	surface density	total(%)	total density
Rho-Olf	OR5M11	Olfactory receptor 5M11	OR5MB	305		56.90	76.82	97.25	522.79		10.60	22.67	42.70	43.69
Rho-Olf	OR5P2	Olfactory receptor 5P2 (Olfactory	OR5P2	322		35.40	103.21	96.25	600.37		7.30	44.03	54.00	34.23
Rho-Olf	OR5P3	Olfactory receptor 5P3 (Olfactory	OR5P3	311		53.30	45.66	96.45	503.56		11.65	47.18	84.05	72.10
Rho-Olf	OR5R1 OR5R1P	Olfactory receptor 5R1 (Olfactory	OR5R1	324		28.50	186.98	95.60	653.12		8.75	38.84	74.95	57.05
Rho-Olf	OR5T1 OR5T1P	Olfactory receptor 5T1 (Olfactory	OR5T1	326		47.90	67.88	95.75	533.08		15.35	26.20	56.25	18.64
Rho-Olf	OR5T2	Olfactory receptor 5T2 (Olfactory	OR5T2	359		45.60	73.93	95.70	670.31		21.00	14.55	83.10	32.38
Rho-Olf	OR5T3	Olfactory receptor 5T3 (Olfactory	OR5T3	340		31.90	52.10	84.40	136.46		6.50	23.63	72.40	21.36
Rho-Olf	OR5V1	Olfactory receptor 5V1 (Hs6M1-2	OR5V1	321		21.30	43.09	75.85	126.49		58.45	29.01	91.75	97.98
Rho-Olf	OR5W2 OR5W2	Olfactory receptor 5W2 (Olfactor	OR5W2	310		33.65	51.81	88.25	421.46		8.65	33.33	71.10	25.77
Rho-Olf	OR6A2 OR6A1 C	Olfactory receptor 6A2 (Olfactory	OR6A2	327		44.65	110.44	93.60	631.20		34.55	19.32	87.75	65.68
Rho-Olf	OR6B1	Olfactory receptor 6B1 (Olfactory	OR6B1	311		18.95	195.19	35.35	77.05		42.25	34.53	88.10	55.08
Rho-Olf	OR6B2 OR6B2P	Olfactory receptor 6B2 (Olfactory	OR6B2	312		42.55	140.64	90.40	439.42		17.65	18.54	83.50	24.86
Rho-Olf	OR6B3 OR6B3P	Olfactory receptor 6B3 (Olfactory	OR6B3	331		33.10	61.39	87.85	267.64		19.70	24.67	61.15	23.13
Rho-Olf	OR6C1	Olfactory receptor 6C1 (OST267)	OR6C1	312		50.20	73.29	95.90	345.48		10.25	36.80	33.25	24.37
Rho-Olf	OR6C2	Olfactory receptor 6C2 (HSA3)	OR6C2	312		28.85	71.77	53.80	53.67		10.00	18.70	69.45	48.22
Rho-Olf	OR6C3	Olfactory receptor 6C3 (HSA8)	OR6C3	311		48.15	161.70	94.25	463.55		39.10	11.83	52.75	13.81
Rho-Olf	OR6C4	Olfactory receptor 6C4 (Olfactory	OR6C4	309		43.70	66.02	91.40	217.88		24.90	31.30	89.20	52.79
Rho-Olf	OR6C6	Olfactory receptor 6C6	OR6C6	314		9.50	107.60	25.30	83.59		14.90	30.13	91.00	39.32
Rho-Olf	OR6F1	Olfactory receptor 6F1 (Olfactory	OR6F1	308		49.35	251.12	60.20	166.79		15.10	18.81	58.10	47.92
Rho-Olf	OR6J1 OR6J2	Olfactory receptor 6J1 (Olfactory	OR6J1	347		26.75	67.29	57.55	58.67		16.65	26.52	72.05	91.09
Rho-Olf	OR6K2	Olfactory receptor 6K2 (Olfactory	OR6K2	324		33.45	70.43	47.95	76.23		37.95	25.52	95.20	96.38
Rho-Olf	OR6K3	Olfactory receptor 6K3 (Olfactory	OR6K3	331		52.30	74.39	97.35	488.63		5.60	27.47	82.85	36.30
Rho-Olf	OR6K6	Olfactory receptor 6K6 (Olfactory	OR6K6	343		27.85	127.74	47.20	86.37		23.55	19.47	46.65	19.10
Rho-Olf	OR6M1	Olfactory receptor 6M1 (Olfactor	OR6M1	313		21.30	43.09	75.85	126.49		33.85	20.68	83.05	34.80
Rho-Olf	OR6N1	Olfactory receptor 6N1	OR6N1	312		75.10	122.65	97.45	888.91		5.90	28.86	14.60	27.78
Rho-Olf	OR6N2	Olfactory receptor 6N2 (Olfactor	OR6N2	317		55.05	47.96	95.75	193.97		48.00	24.50	59.15	25.61
Rho-Olf	OR6P1	Olfactory receptor 6P1 (Olfactory	OR6P1	317		51.30	174.86	68.95	131.72		22.30	35.34	61.75	29.35
Rho-Olf	OR6Q1	Olfactory receptor 6Q1 (Olfactor	OR6Q1	317		57.35	95.44	66.65	94.33		28.45	21.71	91.40	40.88
Rho-Olf	OR6S1	Olfactory receptor 6S1 (Olfactory	OR6S1	331		65.20	80.90	97.45	463.00		23.65	47.49	60.05	50.60
Rho-Olf	OR6T1	Olfactory receptor 6T1 (Olfactory	OR6T1	323		41.65	81.12	95.50	456.60		34.50	24.46	89.65	55.73
Rho-Olf	OR6V1	Olfactory receptor 6V1 (Olfactory	OR6V1	313		19.40	97.96	42.90	85.82		11.90	18.64	75.50	22.68
Rho-Olf	OR6X1	Olfactory receptor 6X1 (Olfactory	OR6X1	312		33.55	61.92	56.95	75.26		24.50	23.39	38.20	19.37
Rho-Olf	OR6Y1 OR6Y2	Olfactory receptor 6Y1 (Olfactory	OR6Y1	325		40.85	163.88	95.95	548.60		40.50	25.48	91.75	43.96
Rho-Olf	OR7A2P OR7A2	Putative olfactory receptor 7A2 (	OR7A2	310		65.90	67.18	96.70	969.74		29.55	21.26	75.20	73.35
Rho-Olf	OR7A5	Olfactory receptor 7A5 (Olfactory	OR7A5	319		24.50	218.91	90.45	920.38		21.00	25.65	58.85	56.64
Rho-Olf	OR7A10	Olfactory receptor 7A10 (OST027	OR7AA	309		48.80	96.67	96.50	590.19		14.60	31.80	82.80	69.23
Rho-Olf	OR7A17	Olfactory receptor 7A17	OR7AH	309		31.20	150.94	92.55	365.71		18.35	32.43	18.65	89.98
Rho-Olf	OR7C1 OR7C4	Olfactory receptor 7C1 (Olfactory	OR7C1	320		16.65	92.67	35.75	70.23		13.25	41.57	45.50	26.09
Rho-Olf	OR7C2 OR7C3	Olfactory receptor 7C2 (Olfactory	OR7C2	319		23.60	146.87	41.65	120.37		12.60	22.29	69.35	67.89
Rho-Olf	OR7D2	Olfactory receptor 7D2 (HTPCRH	OR7D2	312		45.25	160.46	66.05	130.69		20.45	30.31	81.35	37.94

Table S1. Comprehensive Protein Expression Data for 826 GPCRs

Family	Gene_names	Protein_names	Target name	WT-aa-count		Nt_BRIL constructs					ICL3_BRIL constructs			
						surface(%)	surface density	total(%)	total density		surface(%)	surface density	total(%)	total density
Rho-Olf	OR7D4 OR7D4P	Olfactory receptor 7D4 (OR19-B)	OR7D4	312		25.35	155.98	78.15	359.27		37.90	16.86	76.85	34.65
Rho-Olf	OR7G1 OR7G1P	Olfactory receptor 7G1 (Olfactor	OR7G1	311		15.35	60.85	26.85	54.24		24.40	23.20	61.85	29.32
Rho-Olf	OR7G2	Olfactory receptor 7G2 (OST260)	OR7G2	324		31.85	65.76	52.95	85.34		35.35	24.83	95.60	82.88
Rho-Olf	OR7G3	Olfactory receptor 7G3 (OST085)	OR7G3	312		52.10	171.17	89.25	238.04		29.40	19.50	4.10	24.83
Rho-Olf	OR8A1	Olfactory receptor 8A1 (OST025)	OR8A1	326		52.10	110.54	92.70	248.19		26.00	32.37	72.05	43.66
Rho-Olf	OR8B2	Olfactory receptor 8B2 (Olfactory	OR8B2	313		45.20	66.10	88.70	137.45		38.30	25.57	72.15	60.59
Rho-Olf	OR8B3	Olfactory receptor 8B3 (Olfactory	OR8B3	313		68.75	129.71	98.15	906.35		34.65	63.24	87.65	92.58
Rho-Olf	OR8B4 OR8B4P	Olfactory receptor 8B4 (Olfactory	OR8B4	309		52.70	132.00	94.95	290.52		12.50	23.20	75.00	66.12
Rho-Olf	OR8B8	Olfactory receptor 8B8 (Olfactory	OR8B8	311		45.75	82.30	93.55	380.91		34.70	32.96	87.95	85.89
Rho-Olf	OR8B12	Olfactory receptor 8B12 (Olfacto	OR8BC	310		66.50	87.02	96.90	740.40		41.90	28.96	84.65	72.79
Rho-Olf	OR8D1 OR8D3	Olfactory receptor 8D1 (OST004)	OR8D1	308		51.20	107.45	66.90	72.05		57.45	33.70	96.90	169.79
Rho-Olf	OR8D2	Olfactory receptor 8D2 (Olfactor	OR8D2	311		26.45	135.37	91.40	888.74		56.10	24.66	89.25	77.64
Rho-Olf	OR8D4	Olfactory receptor 8D4 (Olfactor	OR8D4	314		52.60	114.92	97.30	581.10		36.05	20.40	79.10	94.03
Rho-Olf	OR8G1 OR8G1P	Olfactory receptor 8G1 (Olfactor	OR8G1	311		61.95	126.13	96.50	460.34		28.05	35.15	61.65	31.74
Rho-Olf	OR8G2 OR8G2P	Olfactory receptor 8G2 (Olfactor	OR8G2	310		21.30	43.09	75.85	126.49		20.40	68.43	94.70	125.71
Rho-Olf	OR8G5 OR8G5P	Olfactory receptor 8G5 (Olfactor	OR8G5	311		33.65	141.68	49.05	111.42		24.15	34.09	73.90	56.00
Rho-Olf	OR8H1	Olfactory receptor 8H1 (Olfactor	OR8H1	311		12.80	88.28	22.20	77.75		19.85	35.17	93.60	285.55
Rho-Olf	OR8H2	Olfactory receptor 8H2 (Olfactor	OR8H2	312		21.90	41.75	37.75	55.34		13.25	35.51	92.10	172.80
Rho-Olf	OR8H3	Olfactory receptor 8H3 (Olfactor	OR8H3	312		9.30	76.40	72.40	218.67		48.80	23.67	78.85	210.14
Rho-Olf	OR8I2	Olfactory receptor 8I2 (Olfactory	OR8I2	310		23.55	114.50	34.15	125.43		16.65	18.03	86.80	28.99
Rho-Olf	OR8J1	Olfactory receptor 8J1 (Olfactory	OR8J1	316		45.10	56.28	94.20	159.89		59.65	45.78	94.10	201.73
Rho-Olf	OR8J2 OR8J2P	Olfactory receptor 8J2	OR8J2	315		23.50	102.56	36.35	87.74		20.40	48.72	96.85	112.58
Rho-Olf	OR8J3	Olfactory receptor 8J3 (Olfactory	OR8J3	315		75.50	146.12	97.25	984.62		46.95	62.79	91.90	159.40
Rho-Olf	OR8K1	Olfactory receptor 8K1 (Olfactory	OR8K1	319		64.75	227.07	95.55	722.62		21.55	46.91	91.90	85.04
Rho-Olf	OR8K3	Olfactory receptor 8K3 (Olfactory	OR8K3	312		25.25	91.43	91.45	810.45		55.50	19.42	91.20	110.08
Rho-Olf	OR8K5	Olfactory receptor 8K5 (Olfactory	OR8K5	307		17.00	177.29	39.85	113.56		20.70	23.78	73.90	26.50
Rho-Olf	OR8S1	Olfactory receptor 8S1	OR8S1	359		55.60	74.14	89.95	183.95		40.70	27.58	84.65	57.63
Rho-Olf	OR8U1	Olfactory receptor 8U1	OR8U1	309		57.30	270.35	91.35	517.63		15.50	35.68	93.50	75.02
Rho-Olf	OR8U8	Olfactory receptor 8U8	OR8U8	319		17.00	165.68	33.20	94.14		18.40	21.07	57.70	23.40
Rho-Olf	OR8U9	Olfactory receptor 8U9	OR8U9	309		24.00	133.78	50.40	172.89		46.10	24.63	96.10	71.76
Rho-Olf	OR9A1P OR9A1	Putative olfactory receptor 9A1 (	OR9A1	263		31.95	168.98	51.75	100.23		39.20	15.62	85.35	104.56
Rho-Olf	OR9A2	Olfactory receptor 9A2 (Olfactor	OR9A2	310		62.85	175.71	93.35	236.74		33.20	23.63	84.05	38.47
Rho-Olf	OR9A4	Olfactory receptor 9A4 (Olfactor	OR9A4	314		30.05	101.68	41.50	109.48		8.50	87.08	73.30	47.31
Rho-Olf	OR9G1 OR9G5	Olfactory receptor 9G1 (Olfactor	OR9G1	305		32.60	148.30	91.90	494.88		22.50	19.90	43.70	27.67
Rho-Olf	OR9G4	Olfactory receptor 9G4 (Olfactor	OR9G4	327		57.55	166.06	92.25	228.77		17.20	32.51	39.80	21.22
Rho-Olf	OR9G9	Olfactory receptor 9G9	OR9G9	305		25.45	66.71	50.90	78.00		4.70	58.18	6.00	16.70
Rho-Olf	OR9I1	Olfactory receptor 9I1 (Olfactory	OR9I1	314		38.60	65.67	46.05	47.62		34.60	16.45	71.75	21.29
Rho-Olf	OR9K2	Olfactory receptor 9K2 (Olfactory	OR9K2	335		60.20	129.41	91.15	205.03		45.55	44.90	87.25	117.56
Rho-Olf	OR9Q1	Olfactory receptor 9Q1	OR9Q1	310		54.85	114.39	94.70	969.12		30.80	20.43	50.00	17.10
Rho-Olf	OR9Q2 OR9Q2P	Olfactory receptor 9Q2	OR9Q2	314		26.70	76.31	57.90	72.94		15.25	21.70	24.25	26.94

Table S1. Comprehensive Protein Expression Data for 826 GPCRs

					Nt_BRIL constructs				ICL3_BRIL constructs			
Family	Gene_names	Protein_names	Target name	WT-aa-count	surface(%)	surface density	total(%)	total density	surface(%)	surface density	total(%)	total density
Rho-Olf	YI035_HUMAN	Putative olfactory receptor ENSP	YI035	320	33.55	170.35	46.50	90.16	29.80	31.17	69.85	214.36
Secretin	CALCR	Calcitonin receptor (CT-R)	CALCR	490	48.00	33.83	69.75	71.75	44.60	56.69	90.90	272.63
Secretin	CALCRL CGRPR	Calcitonin gene-related peptide t	CALRL	461	51.70	38.85	83.95	109.05	47.30	65.12	86.35	285.92
**Secretin**	**CRHR1 CRFR CR**	**Corticotropin-releasing factor re**	**CRFR1**	**444**	**66.15**	**26.22**	**63.55**	**41.54**	**72.75**	**64.23**	**88.50**	**379.74**
Secretin	CRHR2 CRF2R CI	Corticotropin-releasing factor rec	CRFR2	411	64.20	37.67	71.30	72.97	74.40	63.34	91.15	366.92
Secretin	GHRHR	Growth hormone-releasing horm	GHRHR	423	46.55	28.68	60.10	50.66	92.95	87.37	93.50	240.85
Secretin	GIPR	Gastric inhibitory polypeptide re	GIPR	466	62.85	52.74	67.05	73.81	69.15	91.77	93.65	552.05
Secretin	GLP1R	Glucagon-like peptide 1 receptor	GLP1R	463	48.15	191.47	96.05	526.39	61.90	36.12	94.70	390.21
Secretin	GLP2R	Glucagon-like peptide 2 receptor	GLP2R	553	83.45	56.86	98.05	289.13	79.95	55.91	96.50	454.73
**Secretin**	**GCGR**	**Glucagon receptor (GL-R)**	**GLR**	**477**	**61.70**	**129.20**	**97.95**	**812.95**	**76.45**	**87.20**	**92.95**	**364.48**
Secretin	GPR157	Probable G-protein coupled rece	GP157	335	49.00	193.13	96.40	403.56	83.50	214.09	98.00	1,008.88
Secretin	ADCYAP1R1	Pituitary adenylate cyclase-activa	PACR	468	43.55	94.25	86.65	210.96	68.80	33.53	86.75	170.08
Secretin	PTH1R PTHR PTI	Parathyroid hormone/parathyroi	PTH1R	593	68.25	78.20	96.05	464.32	76.65	47.19	93.35	312.46
Secretin	PTH2R PTHR2	Parathyroid hormone 2 receptor	PTH2R	550	37.95	124.17	71.55	168.17	52.25	23.21	94.70	183.50
Secretin	SCTR	Secretin receptor (SCT-R)	SCTR	440	82.95	109.59	93.25	670.27	86.05	55.39	84.75	538.89
Secretin	VIPR1	Vasoactive intestinal polypeptide	VIPR1	457	76.10	96.22	94.40	189.40	85.20	65.81	93.30	376.71
Secretin	VIPR2 VIP2R	Vasoactive intestinal polypeptide	VIPR2	438	77.95	481.97	69.85	344.24	89.50	88.17	91.70	508.94
Glutamate	CASR GPRC2A P(	Extracellular calcium-sensing rec	CASR	1078	95.55	300.04	83.20	368.95	90.35	76.05	91.80	421.15
Glutamate	GABBR1 GPRC3/	Gamma-aminobutyric acid type E	GABR1	961	15.65	180.84	80.50	1,081.00	16.10	25.00	94.20	177.81
Glutamate	GABBR2 GPR51	Gamma-aminobutyric acid type E	GABR2	941	50.85	95.04	90.55	588.89	22.05	27.27	92.45	1,421.65
Glutamate	GPR156 GABABI	Probable G-protein coupled rece	GP156	814	51.65	73.64	86.35	209.32	71.55	58.40	92.40	547.79
Glutamate	GPR158 KIAA11:	Probable G-protein coupled rece	GP158	1215	95.20	338.52	74.10	200.29	90.20	97.12	84.05	308.92
Glutamate	GPR179 GPR158	Probable G-protein coupled rece	GP179	2367	42.55	131.10	94.40	836.43	36.80	26.48	92.60	1,063.37
Glutamate	GPRC5B RAIG2	G-protein coupled receptor fami	GPC5B	403	94.65	230.89	89.00	451.29	13.35	54.31	80.60	112.08
Glutamate	GPRC5C RAIG3 F	G-protein coupled receptor fami	GPC5C	441	96.40	216.00	83.60	303.72	51.45	34.82	96.60	976.30
Glutamate	GPRC5D	G-protein coupled receptor fami	GPC5D	345	96.35	401.57	86.05	522.25	89.85	74.67	88.45	422.86
Glutamate	GPRC6A	G-protein coupled receptor fami	GPC6A	926	30.50	50.82	70.00	254.77	23.70	42.39	91.45	786.95
**Glutamate**	**GRM1 GPRC1A I**	**Metabotropic glutamate recept**	**GRM1**	**1194**	**54.40**	**84.15**	**91.85**	**444.45**	**54.15**	**46.04**	**89.80**	**677.67**
Glutamate	GRM2 GPRC1B I	Metabotropic glutamate recepto	GRM2	872	92.30	100.26	85.40	313.66	43.70	38.69	91.60	1,404.69
Glutamate	GRM3 GPRC1C I	Metabotropic glutamate recepto	GRM3	879	95.90	118.52	93.95	400.44	67.35	37.71	92.50	1,072.46
Glutamate	GRM4 GPRC1D I	Metabotropic glutamate recepto	GRM4	912	36.05	166.09	92.05	769.16	40.80	63.93	88.90	1,478.02
**Glutamate**	**GRM5 GPRC1E I**	**Metabotropic glutamate recept**	**GRM5**	**1212**	**43.15**	**72.23**	**75.65**	**427.15**	**47.20**	**49.80**	**89.90**	**465.23**
Glutamate	GRM6 GPRC1F I	Metabotropic glutamate recepto	GRM6	877	37.00	110.55	84.90	673.38	41.95	52.22	92.25	1,146.17
Glutamate	GRM7 GPRC1G I	Metabotropic glutamate recepto	GRM7	915	21.10	33.63	1.25	115.24	23.55	84.83	92.00	722.59
Glutamate	GRM8 GPRC1H I	Metabotropic glutamate recepto	GRM8	908	40.85	90.56	83.60	730.17	17.60	97.68	92.60	977.58
Glutamate	GPRC5A GPCR5/	Retinoic acid-induced protein 3 (	RAI3	357	93.95	237.05	84.40	347.20	43.80	40.84	92.95	511.91
Glutamate	TAS1R1 GPR70 T	Taste receptor type 1 member 1	TS1R1	841	43.45	92.31	74.20	711.48	33.20	80.27	59.55	228.06
Glutamate	TAS1R2 GPR71 T	Taste receptor type 1 member 2	TS1R2	839	93.25	161.27	75.30	274.74	41.50	68.40	81.80	619.46

Table S1. Comprehensive Protein Expression Data for 826 GPCRs

Family	Gene_names	Protein_names	Target name	WT-aa-count		Nt_BRIL constructs surface(%)	Nt_BRIL constructs surface density	Nt_BRIL constructs total(%)	Nt_BRIL constructs total density		ICL3_BRIL constructs surface(%)	ICL3_BRIL constructs surface density	ICL3_BRIL constructs total(%)	ICL3_BRIL constructs total density
Glutamate	TAS1R3 T1R3 TR	Taste receptor type 1 member 3	TS1R3	852		65.05	145.52	76.65	233.04		54.80	865.22	62.55	1,199.49
Fz/Tas	FZD1	Frizzled-1 (Fz-1) (hFz1) (FzE1)	FZD1	647		96.05	217.67	93.65	323.06		40.90	119.24	68.40	337.68
Fz/Tas	FZD10	Frizzled-10 (Fz-10) (hFz10) (FzE7)	FZD10	581		77.50	105.19	76.20	331.84		74.20	69.38	86.25	512.30
Fz/Tas	FZD2	Frizzled-2 (Fz-2) (hFz2) (FzE2)	FZD2	565		72.40	75.05	91.55	301.21		44.70	100.57	82.35	446.54
Fz/Tas	FZD3	Frizzled-3 (Fz-3) (hFz3)	FZD3	666		85.50	59.60	91.40	538.70		44.90	39.12	65.70	201.69
Fz/Tas	FZD4	Frizzled-4 (Fz-4) (hFz4) (FzE4) (CD	FZD4	537		91.75	107.72	79.50	535.99		80.85	64.73	77.05	352.90
Fz/Tas	FZD5 C2orf31	Frizzled-5 (Fz-5) (hFz5) (FzE5)	FZD5	585		96.50	239.65	88.65	268.41		61.60	74.53	90.05	466.02
Fz/Tas	FZD6	Frizzled-6 (Fz-6) (hFz6)	FZD6	706		71.55	64.56	28.40	112.08		96.45	257.95	85.75	537.51
Fz/Tas	FZD7	Frizzled-7 (Fz-7) (hFz7) (FzE3)	FZD7	574		53.75	97.53	98.45	588.22		65.05	100.64	83.45	296.89
Fz/Tas	FZD8	Frizzled-8 (Fz-8) (hFz8)	FZD8	694		86.35	77.09	69.05	598.51		67.65	98.99	66.60	322.73
Fz/Tas	FZD9 FZD3	Frizzled-9 (Fz-9) (hFz9) (FzE6) (CD	FZD9	591		94.15	197.92	91.20	592.72		31.35	99.11	71.50	437.76
**Fz/Tas**	**SMO SMOH**	**Smoothened homolog (SMO) (P**	**SMO**	**787**		**81.65**	**112.90**	**83.95**	**402.93**		**85.40**	**91.78**	**66.70**	**1,040.40**
Fz/Tas	TAS2R10	Taste receptor type 2 member 1(	T2R10	307		50.40	104.02	91.80	306.68		41.15	217.28	34.10	244.00
Fz/Tas	TAS2R13	Taste receptor type 2 member 1:	T2R13	303		52.00	85.37	95.25	1,087.27		17.05	33.13	65.25	46.25
Fz/Tas	TAS2R14	Taste receptor type 2 member 1	T2R14	317		60.75	50.59	90.35	122.01		9.15	41.40	25.85	27.71
Fz/Tas	TAS2R16	Taste receptor type 2 member 1(	T2R16	291		61.70	108.38	91.35	725.36		23.50	30.70	90.45	174.43
Fz/Tas	TAS2R19 TAS2R	Taste receptor type 2 member 1!	T2R19	299		71.00	42.39	97.70	133.28		36.35	13.48	46.90	17.73
Fz/Tas	TAS2R20 TAS2R	Taste receptor type 2 member 2(	T2R20	309		73.60	31.60	96.65	135.17		34.65	13.87	58.10	19.41
Fz/Tas	TAS2R30 TAS2R	Taste receptor type 2 member 3(	T2R30	319		64.20	47.12	96.15	353.66		44.20	15.64	87.05	34.74
Fz/Tas	TAS2R31 TAS2R	Taste receptor type 2 member 3	T2R31	309		43.35	51.05	95.15	496.84		37.95	14.16	89.25	62.85
Fz/Tas	TAS2R38 PTC	Taste receptor type 2 member 3	T2R38	333		69.90	99.14	97.70	629.86		78.90	33.41	92.40	206.90
Fz/Tas	TAS2R39	Taste receptor type 2 member 3!	T2R39	338		3.70	80.33	18.05	19.81		61.60	29.98	93.75	343.25
Fz/Tas	TAS2R40 GPR60	Taste receptor type 2 member 4(	T2R40	323		63.00	101.83	90.00	560.34		15.05	147.85	58.25	644.40
Fz/Tas	TAS2R41	Taste receptor type 2 member 4	T2R41	307		60.10	80.50	96.90	188.35		42.55	20.58	59.50	26.90
Fz/Tas	TAS2R42 TAS2R!	Taste receptor type 2 member 4	T2R42	314		63.80	86.61	96.80	569.07		51.85	16.43	78.20	26.49
Fz/Tas	TAS2R43	Taste receptor type 2 member 4:	T2R43	309		38.35	52.22	37.90	42.80		40.85	14.17	75.70	22.05
Fz/Tas	TAS2R45 GPR59	Taste receptor type 2 member 4!	T2R45	299		10.90	51.88	4.55	624.55		15.40	18.40	94.05	81.24
Fz/Tas	TAS2R46	Taste receptor type 2 member 4(	T2R46	309		40.00	71.00	43.85	46.69		49.30	15.42	87.60	28.00
Fz/Tas	TAS2R50	Taste receptor type 2 member 5(	T2R50	299		65.20	46.00	96.95	210.13		39.80	14.14	37.85	17.08
Fz/Tas	TAS2R60	Taste receptor type 2 member 6(	T2R60	318		59.20	68.64	96.25	205.18		72.45	72.16	93.80	449.75
Fz/Tas	TAS2R1	Taste receptor type 2 member 1	TA2R1	299		50.40	45.60	82.65	274.50		3.45	25.07	54.45	32.65
Fz/Tas	TAS2R3	Taste receptor type 2 member 3	TA2R3	316		53.75	30.50	77.40	198.38		26.80	24.01	86.55	124.74
Fz/Tas	TAS2R4	Taste receptor type 2 member 4	TA2R4	299		47.50	74.26	87.10	233.38		4.55	24.82	71.50	75.59
Fz/Tas	TAS2R5	Taste receptor type 2 member 5	TA2R5	299		56.35	63.77	93.90	663.58		41.10	31.03	44.00	34.18
Fz/Tas	TAS2R7	Taste receptor type 2 member 7	TA2R7	318		62.65	82.32	92.50	357.14		22.40	47.87	81.65	119.18
Fz/Tas	TAS2R8	Taste receptor type 2 member 8	TA2R8	309		46.75	75.26	91.05	686.28		26.15	38.57	78.35	128.97
Fz/Tas	TAS2R9	Taste receptor type 2 member 9	TA2R9	312		37.55	97.49	81.60	125.20		46.75	75.26	91.05	686.28
Adhesion	BAI1	Brain-specific angiogenesis inhib	BAI1	1584		45.80	180.36	94.20	697.44		10.80	50.97	9.50	35.10

Table S1. Comprehensive Protein Expression Data for 826 GPCRs

					Nt_BRIL constructs				ICL3_BRIL constructs			
Family	Gene_names	Protein_names	Target name	WT-aa-count	surface(%)	surface density	total(%)	total density	surface(%)	surface density	total(%)	total density
Adhesion	BAI2	Brain-specific angiogenesis inhibi	BAI2	1585	73.60	89.17	95.60	585.33	85.35	89.03	91.45	1,043.03
Adhesion	BAI3 KIAA0550	Brain-specific angiogenesis inhibi	BAI3	1522	92.10	236.86	97.25	843.05	96.05	153.58	96.55	1,296.29
Adhesion	CD97	CD97 antigen (Leukocyte antigen	CD97	835	68.55	121.63	87.90	294.42	20.60	34.29	75.50	250.56
Adhesion	CELSR1 CDHF9 F	Cadherin EGF LAG seven-pass G-	CELR1	3014	56.20	70.94	93.30	654.57	95.15	393.58	88.45	491.09
Adhesion	CELSR2 CDHF10	Cadherin EGF LAG seven-pass G-	CELR2	2923	92.80	243.06	85.05	614.18	91.95	222.29	67.60	550.80
Adhesion	CELSR3 CDHF11	Cadherin EGF LAG seven-pass G-	CELR3	3312	94.40	163.13	92.55	812.34	3.50	137.70	0.50	108.65
Adhesion	ELTD1 ETL UNQ2	EGF, latrophilin and seven transn	ELTD1	690	53.05	51.11	90.95	194.67	62.95	35.33	92.20	303.41
Adhesion	EMR1 TM7LN3	EGF-like module-containing muci	EMR1	886	93.35	189.59	92.85	346.78	84.30	83.86	81.95	139.31
Adhesion	EMR2	EGF-like module-containing muci	EMR2	823	62.15	124.49	93.95	635.02	9.25	47.28	7.20	257.96
Adhesion	EMR3 UNQ683/	EGF-like module-containing muci	EMR3	652	84.40	108.34	93.40	474.44	44.65	47.93	63.20	105.12
Adhesion	EMR4P EMR4 GI	Putative EGF-like module-contair	EMR4	457	61.75	168.53	89.05	164.09	18.55	27.49	11.15	21.02
Adhesion	GPR110 PGR19	Probable G-protein coupled rece	GP110	910	65.95	96.62	93.65	558.81	38.15	45.31	90.25	803.32
Adhesion	GPR111 PGR20	Probable G-protein coupled rece	GP111	708	25.15	148.04	36.65	71.81	22.45	28.66	83.90	170.47
Adhesion	GPR112	Probable G-protein coupled rece	GP112	3080	40.65	78.29	90.60	660.77	49.80	44.58	85.70	815.63
Adhesion	GPR113 PGR23 (	Probable G-protein coupled rece	GP113	1079	51.90	113.25	91.45	729.70	43.95	31.71	93.00	245.44
Adhesion	GPR114 PGR27 (	Probable G-protein coupled rece	GP114	528	87.40	68.26	97.60	476.01	39.05	21.71	86.55	114.56
Adhesion	GPR115 PGR18	Probable G-protein coupled rece	GP115	695	48.85	48.78	91.90	353.43	36.75	16.32	90.10	117.22
Adhesion	GPR116 KIAA07!	Probable G-protein coupled rece	GP116	1346	39.85	143.12	92.00	634.84	48.75	30.71	90.15	779.39
Adhesion	GPR123 KIAA182	Probable G-protein coupled rece	GP123	560	90.90	83.07	96.15	579.28	90.75	64.89	94.05	832.12
Adhesion	GPR124 KIAA15:	G-protein coupled receptor 124 (	GP124	1338	95.15	269.49	87.90	728.11	91.70	142.95	83.40	725.57
Adhesion	GPR125 UNQ55(	Probable G-protein coupled rece	GP125	1321	61.15	66.26	74.30	268.21	56.30	42.38	79.00	443.85
Adhesion	GPR126 DREG V	G-protein coupled receptor 126 (	GP126	1221	57.55	95.18	93.65	622.76	66.05	50.16	95.70	930.53
Adhesion	GPR128	Probable G-protein coupled rece	GP128	797	50.85	30.36	86.35	214.53	56.65	36.38	94.15	953.70
Adhesion	GPR133 PGR25	Probable G-protein coupled rece	GP133	874	52.05	97.47	92.40	671.37	24.85	37.15	90.45	219.42
Adhesion	GPR144 PGR24	Probable G-protein coupled rece	GP144	963	5.70	49.38	2.00	36.00	28.35	25.49	86.30	94.48
Adhesion	GPR56 TM7LN4	G-protein coupled receptor 56 (P	GPR56	693	32.50	99.97	33.60	50.62	40.20	18.33	92.15	128.60
Adhesion	GPR64 HE6 TM7	G-protein coupled receptor 64 (H	GPR64	1017	67.50	58.04	93.15	460.95	43.35	29.02	94.45	455.03
Adhesion	GPR97 PGR26	Probable G-protein coupled rece	GPR97	549	49.20	66.74	85.80	137.30	78.70	22.19	94.30	103.63
Adhesion	GPR98 KIAA068(	G-protein coupled receptor 98 (M	GPR98	6306	95.75	180.08	88.25	511.74	84.45	136.71	82.50	490.85
Adhesion	LPHN1 KIAA082	Latrophilin-1 (Calcium-independe	LPHN1	1474	70.80	127.23	92.30	440.39	22.65	23.70	84.70	199.08
Adhesion	LPHN2 KIAA078(	Latrophilin-2 (Calcium-independe	LPHN2	1459	63.75	104.31	88.85	440.91	29.30	25.09	80.95	154.26
Adhesion	LPHN3 KIAA076	Latrophilin-3 (Calcium-independe	LPHN3	1447	87.10	87.63	91.25	293.44	56.75	56.20	84.20	157.15

Table S2. Loop lengths for receptors with surface percentage >80% (upper) and <30% (lower) for both Nt_BRIL and ICL3_BRIL constructs. Structurally determined receptors are shown in blue.

Surface percentage > 80%		Nt	ICL1	ICL2	ICL3	ECL1	ECL2	ECL3	Ct
Rhodopsin	A2A	6	5	10	12	7	38	9	106
	APJ	25	7	8	17	8	35	4	68
	MRGRE	21	6	10	5	6	12	4	29
	OPSB	31	6	10	16	9	28	7	32
	OPSD	34	6	10	16	7	28	7	29
	OPSG	50	6	11	18	6	28	5	25
	5HT4R	14	7	10	39	6	30	5	64
	PAR1	100	6	11	12	6	29	9	38
	S1P1	42	6	10	24	5	29	6	54
	TRFR	22	6	11	44	6	21	5	60
Secretin	SCTR	139	7	10	12	14	17	1	49
Glutamate	CASR	602	8	19	2	7	25	2	208
	GPC5D	20	12	8	11	4	16	11	78
	GP158	354	10	9	10	5	35	7	543
Frizzled/ Taste2	FZD4	212	10	14	8	30	19	13	42
	SMO	252	11	15	10	58	19	39	251
Adhesion	BAI3	843	23	10	70	23	16	15	332
	CELR2	2373	7	10	8	4	18	1	311
	EMR1	498	19	14	39	14	13	8	5
	GPR98	5844	5	9	5	9	25	4	152
	GPR123	10	5	19	46	6	22	4	237
	GPR124	750	10	18	52	5	13	/	426
**Surface percentage <30%**		**Nt**	**ICL1**	**ICL2**	**ICL3**	**ECL1**	**ECL2**	**ECL3**	**Ct**
Rhodopsin	DR5R	35	7	10	33	4	43	16	106
	MC3R	34	6	17	20	17	8	15	8
	GP132	38	6	10	7	8	15	16	70
	NPY2	49	9	7	9	6	28	7	37
Secretin	GP111	413	22	12	38	31	18	2	/
	GP144	623	22	9	43	20	15	11	23
Glutamate	mGluR7	588	10	16	9	7	29	11	65
	$GABA_{B1}$	589	10	16	10	12	35	8	97
Frizzled/ Taste2	T2R45	1	33	7	30	22	31	9	19

Table S3. Loop lengths for receptors with surface percentage >80% for the Nt_BRIL constructs. Structurally determined receptors are shown in blue.

Surface percentage > 80% for Nt_BRIL		Nt	ICL1	ICL2	ICL3	ECL1	ECL2	ECL3	Ct
Rhodopsin	**5HT4R**	**14**	**4**	**9**	**113**	**6**	**21**	**10**	**64**
	**A2A**	**6**	**5**	**10**	**12**	**7**	**38**	**9**	**106**
	**APJ**	25	7	8	17	8	35	4	68
	**ADA2B**	**8**	**5**	**9**	**6**	**16**	**51**	**6**	**8**
	**CML1**	37	6	8	8	7	39	/	39
	**EDNRA**	76	4	9	12	11	26	11	43
	**ETNB2**	97	5	9	11	6	28	11	52
	**GP101**	26	5	9	170	6	20	7	51
	**GPC39**	26	14	20	41	20	45	18	105
	**GPR26**	1	6	9	30	6	23	/	28
	**GPR82**	10	5	27	10	11	21	9	23
	**GPR85**	16	6	9	16	7	56	4	12
	**LGR4**	535	19	8	6	5	23	6	132
	**MRGRD**	24	4	8	9	6	6	?	27
	**MRGRE**	21	6	10	5	6	12	4	29
	**MRGRF**	41	4	8	8	8	14	?	34
	**OPSB**	31	6	10	16	9	28	7	32
	**OPSD**	34	6	10	16	7	28	7	29
	**OPSG**	50	6	11	18	6	28	5	25
	**PAR1**	**100**	**6**	**11**	**12**	**6**	**29**	**9**	**38**
	**PAR3**	90	5	9	5	7	26	7	12
	**RGR**	15	16	18	23	18	24	7	23
	**S1P1**	**42**	**6**	**10**	**24**	**5**	**29**	**6**	**54**
	**TRFR**	22	5	12	36	5	22	6	60
	**O10X1**	41	8	19	20	24	37	10	21
	**O56A4**	28	8	19	20	24	36	15	16
Secretin	**GLP2R**	175	6	9	7	17	9	7	114
	**SCTR**	139	7	10	12	14	17	1	49
Glutamate	**CASR**	602	8	19	2	7	25	2	208
	**GPC5B**	53	12	8	10	5	12	12	104
	**GPC5C**	47	12	8	10	7	27	11	134
	**GPC5D**	20	12	8	11	4	16	11	76
	**GRM2**	565	10	14	8	5	23	8	51
	**GRM3**	574	10	15	8	5	23	8	50
	**GP158**	354	10	9	10	5	35	7	543
	**RAI3**	33	14	11	15	8	26	14	89
	**TS1R2**	563	10	15	8	5	24	6	20
Frizzled/ Taste2	**FZD1**	312	6	7	7	26	19	21	26
	**FZD3**	195	7	6	7	27	19	14	168
	**FZD4**	212	10	14	8	30	19	13	42
	**FZD5**	168	6	8	7	23	19	8	64
	**FZD8**	270	6	8	9	62	19	8	90
	**FZD9**	224	6	8	7	25	19	17	63
	**SMO**	**252**	**11**	**15**	**10**	**58**	**19**	**39**	**251**
Adhesion	**BAI3**	843	23	10	70	23	16	15	332

**CELR2**	2373	7	10	8	4	18	1	311
**CELR3**	2536	7	9	10	5	10	/	538
**EMR1**	498	19	14	39	14	13	8	5
**EMR3**	325	17	13	36	17	14	6	18
**GPR98**	5844	5	9	5	9	25	4	152
**GP114**	213	21	10	35	19	27	4	0
**LPHN3**	861	5	8	6	5	14	4	343
**GPR123**	10	5	19	46	6	22	4	237
**GPR124**	750	10	18	52	5	13	/	426

Table S4. Loop lengths for receptors with surface percentage >80% for the ICL3_BRIL constructs. Structurally determined receptors are shown in blue.

Surface percentage > 80% for ICL3_BRIL		NT	ICL1	ICL2	ICL3	ECL1	ECL2	ECL3	CT
	**5HT1A**	32	5	9	113	6	21	10	7
	**5HT2C**	53	5	9	60	6	19	7	71
	**5HT4R**	14	7	10	39	6	30	5	64
	**5HT6R**	21	4	9	36	5	21	4	105
	**5HT7R**	77	5	10	51	7	19	12	84
	**A2A**	**6**	**5**	**10**	**12**	**7**	**38**	**9**	**106**
	**APJ**	25	7	8	17	8	35	4	68
	**ACM1**	21	5	9	140	6	22	5	27
	**ACM5**	26	6	9	212	6	22	6	22
	**ADA1A**	21	5	9	57	6	17	6	125
	**ADRB1**	53	5	9	56	6	25	5	86
	**ADRB3**	32	5	9	44	6	26	6	48
	**AGTR1**	25	5	8	13	7	25	6	39
	**BRS3**	43	5	9	15	6	26	7	55
	**C5AR**	34	4	13	6	7	21	5	46
	**CCR7**	52	5	10	6	5	19	10	36
	**CXCR1**	33	7	8	7	5	20	10	29
	**CXCR2**	45	5	12	7	5	21	8	42
	**DUFFY**	63	11	13	16	13	20	22	28
	**EDNRB**	97	5	9	28	6	28	11	52
	**GALR2**	22	5	8	12	7	16	8	79
	**GP135**	100	5	8	5	13	25	9	72
**Rhodopsin**	**GP153**	3	5	9	21	10	55	685	206
	**GP162**	9	5	9	58	11	20	6	254
	**GP173**	17	5	11	25	8	54	4	17
	**GPBAR**	11	6	8	21	7	18	11	30
	**GPR83**	67	5	7	14	6	26	1	63
	**GPR87**	39	6	8	/	5	18	52	40
	**HRH1**	23	4	9	188	6	23	5	3
	**HRH4**	11	5	8	92	5	18	7	16
	**LPAR1**	**46**	**7**	**8**	**14**	**8**	**19**	**8**	**37**
	**MRGRE**	21	6	10	5	6	12	4	29
	**NPY1R**	36	6	8	13	6	29	5	48
	**O5AL1**	44	8	22	12	19	42	13	21
	**OPSB**	31	6	10	16	9	28	7	32
	**OPSD**	34	6	10	16	7	28	7	29
	**OPSG**	50	6	11	18	6	28	5	25
	**OPSR**	49	5	9	15	7	26	7	26
	**PAR1**	100	6	11	12	6	29	9	38
	**P2YR2**	30	5	8	7	8	20	4	67
	**PD2R2**	29	4	8	8	7	33	4	73
	**RL3R1**	78	6	20	27	7	28	6	63
	**S1P1**	**42**	**6**	**10**	**24**	**5**	**29**	**6**	**54**
	**S1P5**	32	5	9	39	9	/	11	76
	**TRFR**	22	5	12	36	5	22	6	60

	**UR2R**	45	5	8	8	6	16	4	59
**Secretin**	**GHRHR**	126	7	9	9	8	9	3	44
	**GP157**	40	0	10	27	5	5	16	33
	**SCTR**	139	7	10	12	14	17	1	49
	**VIPR1**	123	7	9	9	7	8	5	60
	**VIPR2**	139	7	8	9	11	9	4	66
**Glutamate**	**CASR**	602	8	19	2	7	25	2	208
	**GP158**	354	10	9	10	5	35	7	543
	**GPC5D**	20	12	8	11	4	16	11	78
**Frizzled/ Taste2**	**FZD4**	212	10	14	8	30	19	13	42
	**FZD6**	191	6	8	7	27	19	14	212
	**SMO**	**252**	**11**	**15**	**10**	**58**	**19**	**39**	**251**
**Adhesion**	**BAI2**	898	21	8	68	18	15	14	340
	**BAI3**	843	23	10	70	23	16	15	332
	**CELR1**	2465	5	8	9	5	14	/	310
	**CELR2**	2373	7	10	8	4	18	1	311
	**EMR1**	498	19	14	39	14	13	8	5
	**GPR98**	5844	5	9	5	9	25	4	152
	**GPR123**	10	5	19	46	6	22	4	237
	**GPR124**	750	10	18	52	5	13	/	426

```
      1       10        20        30        40        50        60
ADRB1 MGAGVLVLGASEPGNLSSAAPLPDGAATAARLLVPASPPASLLPPASESPEPLSQQWTAG
ADRB3 ........MAPWPHENSSLAPWPD.....LPTLAPNTANTSGLPGVP.........WEAA
ADRB2 .........MGQPGNGSAFLLAPN......GSHAPDHDVTQER..........DEVWVVG

              70        80        90       100       110
ADRB1 MG.LLMALIVLLIVAGNVLVIVAIAKTPRLQTLTNLFIMSLASADLVMGLLVVPFGATIV
ADRB3 LAGALLALAVLATVGGNLLVIVAIAWTPRLQTMTNVFVTSLAAADLVMGLLVVPPAATLA
ADRB2 MG.IVMSLIVLAIVFGNVLVITAIAKFERLQTVTNYFITSLACADLVMGLAVVPFGAAHI

   120       130       140       150       160       170
ADRB1 VWGRWEYGSFFCELWTSVDVLCVTASIETLCVIALDRYLAITSPFRYQSLLTRARARGLV
ADRB3 LTGHWPLGATGCELWTSVDVLCVTASIETLCALAVDRYLAVTNPLRYGALVTKRCARTAV
ADRB2 LMKMWTFGNFWCEFWTSIDVLCVTASIETLCVIAVDRYFAITSPFKYQSLLTKNKARVII

   180       190       200       210       220       230
ADRB1 CTVWAISALVSFLPILMHWWRAESD.EARRCYNDPKCCDFVTNRAYAIASSVVSFYVPLC
ADRB3 VLVWVVSAAVSFAPIMSQWWRVGADAEAQRCHSNPRCCAFASNMPYVLLSSSVSFYLPLL
ADRB2 LMVWIVSGLTSFLPIQMHWYRATHQ.EAINCYANETCCDFFTNQAYAIASSIVSFYVPLV

    240       250       260       270       280       290
ADRB1 IMAFVYLRVFREAQKQVKKIDSCERRFLGGPARPPSPSPSPVPAPAPPPGPPRPAAAAAT
ADRB3 VMLFVYARVFVVATRQLRLLRGELGRFPP.EESPPAPSRSLAPAPVGTCAPPEGVPACG.
ADRB2 IMVFVYSRVFQEAKRQLQKIDKSEGRFHVQNLSQVEQ...............DGRTGHG.

   300       310       320       330       340       350
ADRB1 APLANGRAGKRRPSRLVALREQKALKTLGIIMGVFTLCWLPFFLANVVKAFHR.ELVPDR
ADRB3 ..........RRPARLLPLREHRALCTLGLIMGTFTLCWLPFFLANVLRALGGPSLVPGP
ADRB2 ..........LRRSSKFCLKEHKALKTLGIIMGTFTLCWLPFFIVNIVHVIQD.NLIRKE

    360       370       380       390       400       410
ADRB1 LFVFFNWLGYANSAFNPIIYCRSPDFRKAFQRLLC.CARRAARRRHATHGDRPRASGCLA
ADRB3 AFLALNWLGYANSAFNPLIYCRSPDFRSAFRRLLCRCGRR...........LPPEPCAAA
ADRB2 VYILLNWIGYVNSGFNPLIYCRSPDFRIAFQELLC..LRR...............SSLKA

    420       430       440       450       460       470
ADRB1 RPGPPPSPGAASDDDDDDVVGATPPARLLEPWAGCNGGAAADSDSSLDEPCRPGFASESK
ADRB3 RPALFPSGVPAARS.......SPAQPRLCQRLDGASWGVS....................
ADRB2 YGNGYSSNGNTGEQSGYHVEQEKENKLLCEDLPGTEDFVGHQGTVPSDNIDSQGRNCSTN

ADRB1 V...
ADRB3 ....
ADRB2 DSLL
```

Figure S1 Sequence alignment between adrenergic receptor subfamily

Boxes with white mark represent the high conserved domain and dots represent the gap between two residues.
